# Trends in epidemiological characteristics and etiologies of diarrheal disease in children under five: an ecological study based on Global Burden of Disease study 2021

**DOI:** 10.1016/j.soh.2024.100086

**Published:** 2024-11-01

**Authors:** Chu Chu, Guobing Yang, Jian Yang, Defeng Liang, Ruitao Liu, Guanhua Chen, Jichun Wang, Guisheng Zhou, Hongli Wang

**Affiliations:** a National Key Laboratory on Technologies for Chinese Medicine Pharmaceutical Process Control and Intelligent Manufacture, Nanjing University of Chinese Medicine, Nanjing 210023, Jiangsu, China; b Gansu Provincial Center for Disease Control and Prevention, Lanzhou 730000, Gansu, China; c Chinese Center for Disease Control and Prevention, National Key Laboratory of Intelligent Tracking and Forecasting for Infectious Diseases, Beijing 102206, China; d Guangzhou Women and Children's Medical Center, Guangzhou Medical University, Guangzhou 510623, Guangdong, China

**Keywords:** Diarrheal disease, Children, Global Burden of Disease study 2021, Projections, One Health

## Abstract

**Background:**

Diarrhea remains a significant health threat to children under five years of age. The study aims to systematically elucidate the global burden of diarrhea in children, providing scientific insights for effective prevention and control strategies.

**Methods:**

The data from the Global Burden of Disease (GBD) 2021 study was analyzed to assess the trends in incidence, prevalence, mortality, and disability-adjusted life years (DALYs) of diarrhea in children under five years across the globe, 21 geographical region, and 204 countries and territories, stratified by age group, sex, and socio-demographic index (SDI) levels. The trend of the disease burden for childhood diarrhea from 1990 to 2021 was described and estimated using the average annual percent change (AAPC), and a Bayesian age-period-cohort (BAPC) model was employed to predict the future burden of diarrhea in children.

**Results:**

From 1990 to 2021, there was a significant decline in the global burden of diarrhea among children under five years of age. The AAPC for incidence (−4092.18, 95% confidence interval [*CI*]: −4224.60 to −3959.76), prevalence (−70.98, 95% *CI*: −72.67 to −69.28), mortality (−6.89, 95% *CI*: −6.95 to −6.83), and DALYs rate (−621.79, 95% *CI*: −627.20 to −616.38) of diarrhea in children all showed a marked downward trend. Diarrheal incidence (*r* = −0.782, *P* < 0.001), prevalence (*r* = −0.777, *P* < 0.001), mortality (*r* = −0.908, *P* < 0.001), and DALYs rate (*r* = −0.904, *P* < 0.001) were negatively correlated with the SDI. Between 2022 and 2035, the global incidence, prevalence, and mortality rates of diarrhea in children under five years are projected to continue declining. The leading causes of diarrheal mortality in children include wasting, underweight, and non-exclusive breastfeeding. Rotavirus remains the predominant pathogen associated with diarrhea-related mortality rate and DALY rate.

**Conclusion:**

Although the global burden of diarrhea in children under five has steadily declined, it remains a significant health threat. Rotavirus is the leading pathogen, highlighting the importance of expanding rotavirus vaccination. Additionally, improving nutritional status, increasing exclusive breastfeeding rates, and enhancing access to sanitation and clean drinking water are crucial measures that, when widely implemented, can effectively reduce the health risks posed by diarrhea in children.

## 1 Introduction

Diarrhea is characterized by passing stool more than three times a day, with abnormally loose or watery consistency [[1], [2], [3]]. The causes of diarrhea are diverse and include food allergies, irritable bowel syndrome, gastrointestinal tumors, and infections of the digestive tract [4,5]. Among these, gastrointestinal infections are one of the leading causes. Pathogens responsible for diarrhea include viruses (e.g., rotavirus, norovirus, astrovirus, and adenovirus), bacteria (e.g., enteropathogenic *Escherichia coli*, *Shigella*, *Campylobacter*, non-typhoidal *Salmonella*, and *Clostridium difficile*), and parasites (e.g., *Entamoeba histolytica*, *Cryptosporidium*, *Giardia lamblia,* helminths, worms, etc.) [2,[6], [7], [8], [9]].

Diarrhea poses a significant health threat to children under five years of age. It can cause substantial loss of fluids and electrolytes, leading to dehydration, which may result in shock, organ failure, or even death [5,10]. In addition, diarrhea impairs nutrient absorption, contributing to malnutrition, which not only affects growth and development but can also lead to underweight and stunted growth [5,10]. Recurrent diarrhea weakens the immune system, making children more susceptible to other infections, creating a vicious cycle of immune dysfunction [11]. Certain pathogens, such as *Shigella* or *Campylobacter*, can cause severe complications, including sepsis, intestinal perforation, and acute kidney failure [12]. Diarrhea remains one of the leading causes of child mortality globally, with an estimated 500,000 children under five dying each year from diarrhea and its complications, particularly in low- and middle-income countries, with the highest burden seen in Sub-Saharan Africa and South Asia [3,13,14].

A comprehensive understanding of the burden and epidemiological trends of diarrheal disease among children under five is critical for guiding the development of effective control policies and strategies. Since the 1990s, many studies have aimed to estimate the global burden of diarrheal disease in children. However, systematic and comparative studies of the global burden of childhood diarrhea remain inconsistent. The Global Burden of Disease (GBD) study has provided a comprehensive and systematic approach to evaluate a wide range of global health issues since 1990 [[15], [16], [17], [18], [19]]. Therefore, the data from the GBD was used to analyze the incidence, prevalence, mortality, and disability-adjusted life years (DALYs) of diarrheal disease in children under five years, stratified by sex, socio-demographic index (SDI), and etiology at the range of global, super-region, and 204 countries and territories, from 1990 to 2021 [18,19]. The study aims to provide data on the burden of diarrheal diseases in children under five for international organizations, governments, health agencies, researchers, and clinicians. The findings will help identify the epidemiological characteristics and burden of diarrheal diseases in children, providing evidence for the formulation of scientific prevention and control strategies.

## 2 Methods

### 2.1 Data source

The GBD study 2021 assessed the disease burden of 371 diseases and injuries and 88 risk factors across the globe, super-region, and 204 countries and territories [18,19]. The incidence, prevalence, mortality, and DALYs of each disease for different age groups and regions were model-based estimate. The study utilized the Disease Modeling-Bayesian Meta-Regression (DisMod-MR) tool (version 2.1), employing Bayesian priors, regularization, and multi-dimensional smoothing modeling. Mortality data in GBD 2021 were primarily sourced from national death registration systems, maternal and child health surveillance networks, and population census data. Incidence and prevalence data were derived from disease surveillance systems, national health surveys, and published literature. The burden of disability was estimated using data from case notifications, hospital discharge databases, household surveys, and cohort studies [18,19]. Detailed information on the study design, data collection, and estimation methods can be found elsewhere [18,19].

From the disease in the GBD study 2021, data from the Global Enteric Multicenter Study (GEMS)—a seven-site case–control study on moderate-to-severe diarrhea in children under five years—were utilized to calculate odds ratios for diarrheal pathogens [2,9,10]. The raw data were systematically reanalyzed, ensuring representation of the distribution of cases and controls by age and site, with pathogens detected using quantitative polymerase chain reaction (qPCR) [20]. For data that did not employ qPCR, adjustments were made for sensitivity and specificity prior to modeling to standardize the data across different detection methods. This pre-modeling adjustment improved consistency, particularly for non-qPCR datasets, and provided more accurate control of extreme values, while capturing uncertainty in the model [[18], [19], [20]].

The SDI is a composite measure that reflects the social and economic conditions affecting health outcomes in different locations. It is calculated as the geometric mean of three indicators: total fertility rate among individuals younger than 25 years, mean years of education for those aged 15 years or older, and lag-distributed income per capita [18,19]. In the GBD study 2021, SDI values were multiplied by 100 to provide a scale ranging from 0 to 100 [18,19]. They are grouped into five development levels: low (< 0.46), low-middle (0.46–0.60), middle (0.61–0.69), high-middle (0.70–0.81), and high (> 0.81) [16,18,19].

### 2.2 Data sources

Data were obtained from the Institute for Health Metrics and Evaluation website (https://vizhub.healthdata.org), including the incidence, prevalence, mortality, and DALYs rates and numbers for children under five years with 95% uncertainty intervals (*UI*s), disaggregated by sex, region, super-region, and 204 countries and territories from 1990 to 2021 [18,19]. The classification of diarrheal diseases followed the International Classification of Diseases (ICD) by the World Health Organization (WHO), using ICD-9 codes 001–009.9 and ICD-10 codes A00–A09 [18,19].

### 2.3 Statistical analysis

The percentage changes in the numbers and rates (incidence, prevalence, mortality, and DALYs) between 1990 and 2021 were calculated using the following formula [18,19]:

Percentage change = (value_2021_－value_1990_)/ value_2021_ × 100%

The GBD study 2021 database reports *UI*s rather than precise statistical values. As a result, statistical significance cannot be directly calculated when comparing two numerical values (e.g., numbers, rates, or percentages). Overlapping *UI*s indicate no significant difference (*P* > 0.05), whereas non-overlapping *UI*s suggest a statistically significant difference (*P* < 0.05) [15,16].

In addition, the estimated annual percentage changes (EAPCs) for rates and numbers were calculated to illustrate trends [21]. The EAPC was derived using the formula: EAPC = 100 × (e^*β*^－1), where the 95% confidence intervals (*CI*s) for the EAPC were based on the estimation of *β*. An increasing trend was indicated if both the EAPC estimate and the lower limit of its 95% *CI* were greater than zero. Conversely, a decreasing trend was identified if both the EAPC estimate and the upper limit of its 95% *CI* were below zero. If neither condition was met, the trend was considered stable [22].

Smoothing spline models were employed to assess the association between the burden of diarrheal disease (rates) and the SDI across the globe, five SDI regions, 21 geographical regions, and 204 countries and territories [15,23,24]. Locally weighted scatterplot smoothing was applied, with the degree, number, and location of knots automatically determined based on the data and the span parameter [15,23]. Spearman's rank correlation coefficient was used to confirm the correlation between burden of diarrheal disease (rates) and SDI [15,23]. A *P*-value of <0.05 was considered statistically significant.

The joinpoint regression model provides a detailed linear analysis of the long-term trends in the incidence, prevalence, mortality, and DALYs rates of diarrheal diseases for children under five years [25]. The standard error was calculated using the formula:

Standard error= (upper limit－lower limit) / (1.96 × 2)

The joinpoint regression model divides the trend lines into distinct phases, calculating the average annual percent change (AAPC) to represent the overall change of rate across the entire period, and the annual percent change (APC) to capture rate variations within specific time intervals [26,27]:

$$\text{AP}C_{i}=\left\{\exp\left(\beta_{i}\right)-1\right\}\times 100\%$$

$$\text{AAP}C_{i}=\left\{\exp\left(\frac{\sum W_{i}\beta_{i}}{\sum W_{i}}\right)-1\right\}\times 100\%$$

In this model, the number and position of the joinpoint was determined using the grid search method (GSM) [28]. In the model, *i* is the number of segments, *β*_*i*_ corresponds to the regression coefficients for each linear segment of the data. *W*_*i*_ is the regression coefficient weights represented by the length of each corresponding segment. Model selection was optimized using the Monte Carlo permutation test and the modified Bayesian Information Criterion, with the model that minimized the mean squared error being selected [29]. Both the APC and AAPC are unitless relative measures that indicate the direction and rate of trend changes. A negative AAPC or APC, with the upper limit of the 95% *CI* below zero, suggests a declining trend over time. Conversely, a positive AAPC or APC, with the lower limit of the 95% *CI* above zero, indicates an increasing trend. If neither condition is met, the rate is considered stable, with no significant change over the observed period [30,31].

The Bayesian age-period-cohort (BAPC) model was used to predict the global incidence, prevalence, and mortality of diarrheal disease from 2022 to 2035, with 95% *CI*s calculated [32]. The BAPC model accounts for age, period, and cohort factors. Age is a key risk factor for many diseases, while period and birth cohort serve as proxies for other unmeasured factors. The BAPC model is a log-linear Poisson model that assumes multiplicative effects of age, period, and cohort [15,16,32]:

$$\log\left(\lambda_{ij}\right)=\alpha +\mu_{i}+\beta_{j}+\gamma_{k}$$

In this model, *i* (1 ≤ *i* ≤ *I*) represents the time points, j (1 ≤ j ≤ J) represents the age groups, α denotes the intercept, *μ*_*i*_ indicates the age effect, *β*_*j*_ represents the period effect, *γ*_*k*_ denotes the cohort effect. BAPC model utilized integrated nested laplace approximation (INLA) to approximate the posterior marginal distribution, thereby avoiding issues related to mixing and convergence, and demonstrating relatively low error rates [33]. The prior distribution for age, period, and cohort effects was set as an inverse gamma distribution. A second-order random walk model was applied to adjust for overdispersion. Predictions were made using the R-BAPC and R-INLA packages [15,16].

All statistical analyses were performed using *R* software (version 4.4.1, R Foundation for Statistical Computing, Vienna, Austria; available at https://cran.r-project.org).

## 3 Results

### 3.1 Global

The incidence rate of diarrhea in children under five years decreased from 190,036.82 per 100,000 population in 1990 (95% *UI*: 161,363.74–220,431.93 per 100,000 population) to 59,677.27 per 100,000 population in 2021 (95% *UI*: 149,246.22–70,442.60 per 100,000 population), showing a significant downward trend (AAPC = −4092.18, 95% *CI*: −4224.60 to −3959.76) (Table 1). The number of cases declined from 1.18 billion in 1990 (95% *UI*: 1.00–1.37 billion) to 392.78 million in 2021 (95% *UI*: 324.12–463.63 million), also demonstrating a clear decreasing trend (Table S1).

**Table 1:** The incidence rate of diarrheal disease among children under five years in 1990 and 2021, and the changing trend of incidence rate across different GBD regions.

Location	Incidence rate per 100,000 population (95% *UI*) in 1990	Incidence rate per 100,000 population (95% *UI*) in 2021	Percentage change of incidence rate (95% *UI*) in 1990–2021/%	AAPC of incidence rate (95% *CI*) in 1990–2021/%
Global	190,036.82 (161,363.74–220,431.93)	59,677.27 (49,246.22–70,442.60)	−68.60 (−70.19 to −66.63)	−4092.18 (−4224.60 to −3959.76)
East Asia	104,006.49 (82,013.64–131,205.90)	14,742.90 (10,627.07–19,781.98)	−85.83 (−87.88 to −83.24)	−2960.49 (−3003.42 to −2917.57)
Southeast Asia	248,000.47 (212,584.70–281,001.18)	111,478.52 (91,322.47–135,033.26)	−55.05 (−58.67 to −50.41)	−4164.92 (−4302.15 to −4027.69)
Oceania	187,663.02 (163,852.48–213,114.60)	128,542.07 (107,231.53–150,654.43)	−31.50 (−38.15 to −23.93)	−2167.86 (−2294.18 to −2041.53)
Central Asia	142,467.30 (125,857.87–160,469.60)	18,127.20 (14,904.27–21,608.89)	−87.28 (−88.75 to −85.87)	−3973.18 (−4028.19 to −3918.18)
Central Europe	22,642.57 (17,810.52–28,421.52)	9106.30 (7028.84–11,423.00)	−59.78 (−61.76 to −57.92)	−425.85 (−446.07 to −405.63)
Eastern Europe	116,613.14 (92,852.91–145,267.41)	26,040.53 (19,294.85–33,600.13)	−77.67 (−79.85 to −74.91)	−2939.59 (−2987.84 to −2891.34)
High-income Asia Pacific	67,374.35 (47,636.16–91,142.81)	84,612.72 (60,477.92–112,172.01)	25.59 (17.49–34.20)	561.60 (498.80–624.41)
Australasia	32,930.58 (23,674.52–43,552.97)	11,196.47 (8319.09–14,674.70)	−66.00 (−68.95 to −62.97)	−654.46 (−723.85 to −585.08)
Western Europe	84,540.02 (59,028.64–114,761.61)	73,024.26 (52,433.84–96,060.38)	−13.62 (−17.84 to −9.58)	−567.95 (−687.11 to −448.78)
Southern Latin America	11,2707.95 (92,207.81–136,429.58)	24,808.92 (18,397.90–32,314.12)	−77.99 (−80.47 to −75.21)	−2780.67 (−2901.99 to −2659.35)
High-income North America	34,799.33 (23,898.70–48,779.02)	3133.27 (2437.97–3988.19)	−91.00 (−92.05 to −89.65)	−1134.00 (−1256.87–1011.14)
Caribbean	119,415.30 (101,792.96–137,246.08)	38,349.64 (31,329.31–46,183.04)	−67.89 (−70.71 to −65.02)	−2600.47 (−2650.35 to −2550.59)
Andean Latin America	298,341.14 (274,448.62–320,996.02)	30,127.88 (23,855.90–37,159.56)	−89.90 (−91.64 to −88.05)	−8789.01 (−8902.36 to −8675.65)
Central Latin America	140,741.46 (115,360.75–168,584.09)	32,084.93 (25,506.19–39,269.03)	−77.20 (−79.06 to −75.06)	−3509.99 (−3690.22 to −3329.77)
Tropical Latin America	168,221.48 (134,782.50–204,546.76)	33,785.22 (25,772.38–42,958.65)	−79.92 (−81.54 to −78.28)	−4458.78 (−4559.19 to −4358.36)
North Africa and middle East	207,714.03 (180,852.51–235,848.08)	52,841.49 (43,771.71–62,111.00)	−74.56 (−76.41 to −72.61)	−4995.55 (−5083.05 to −4908.06)
South Asia	245,339.91 (201,409.66–288,523.89)	67,464.36 (55,874.31–80,365.75)	−72.50 (−75.24 to −69.61)	−5795.93 (−5938.69 to −5653.16)
Central Sub-Saharan Africa	284,869.93 (251,509.28–313,343.25)	78,226.09 (65,314.85–91,468.22)	−72.54 (−75.31 to −69.23)	−7202.70 (−7457.16 to −6948.23)
Eastern Sub-Saharan Africa	287,099.13 (253,703.12–315,663.24)	65,906.62 (56,815.54–75,948.77)	−77.04 (−78.88 to −74.92)	−7186.52 (−7278.08 to −7094.96)
Southern Sub-Saharan Africa	276,250.38 (238,171.64–307,177.75)	58,170.76 (48,238.40–69,008.00)	−78.94 (−81.30 to −76.09)	−7184.05 (−7293.31 to −7074.78)
Western Sub-Saharan Africa	302,392.13 (270,575.89–329,265.97)	87,324.99 (73,552.10–102,170.00)	−71.12 (−74.03 to −67.42)	−6978.14 (−7072.77 to −6883.51)
High-middle SDI region	103,449.61 (83,384.50–126,918.36)	28,839.35 (21,847.11–37,098.64)	−72.12 (−74.63 to −69.08)	−2393.91 (−2453.79 to −2334.03)
High SDI region	60,947.41 (44,147.87–81,455.25)	40,284.05 (28,810.45–53,250.29)	−33.90 (−37.97 to −29.85)	−699.24 (−791.83 to −606.64)
Low-middle SDI region	251,624.87 (215,250.71–286,288.87)	69,133.80 (58,047.60–81,026.57)	−72.53 (−74.24 to −70.53)	−6050.90 (−6171.98 to −5929.81)
Low SDI region	279,033.44 (244,801.18–310,490.10)	79,236.37 (66,678.62–92,048.61)	−71.60 (−73.63 to −69.15)	−6591.68 (−6706.93 to −6476.43)
Middle SDI region	176,443.85 (146,844.76–208,421.94)	49,207.02 (40,006.75–59,304.93)	−72.11 (−73.49 to −70.54)	−3972.06 (−4045.51 to −3898.61)

Abbreviations: GBD, Global Burden of Disease; AAPC, average annual percent change; *CI*, confidence interval; *UI*, uncertainty interval; SDI, socio-demographic index.

The prevalence rate of diarrhea among children under five decreased from 3138.81 per 100,000 population in 1990 (95% *UI*: 2749.19–3557.51 per 100,000 population) to 885.07 per 100,000 population in 2021 (95% *UI*: 755.93–1029.39 per 100,000 population), showing a significant downward trend (AAPC = −70.98, 95% *CI*: −72.67 to −69.28) (Table 2). The number of prevalent cases also declined, from 19.46 million in 1990 (95% *UI*: 17.04–22.05 million) to 5.83 million in 2021 (95% *UI*: 4.98–6.78 million), demonstrating a clear decrease (Table S2).

**Table 2:** The prevalence rate of diarrheal disease among children under five years in 1990 and 2021, and the changing trend of prevalence rate across different GBD regions.

Location	Prevalence rate per 100,000 population (95% *UI*) in 1990	Prevalence rate per 100,000 population (95% *UI*) in 2021	Percentage change of prevalence rate (95% *UI*) in 1990–2021/%	AAPC of prevalence rate (95% *CI*) in 1990–2021/%
Global	3138.81 (2749.19–3557.51)	885.07 (755.93–1029.39)	−71.80 (−73.52 to −69.93)	−70.98 (−72.67 to −69.28)
East Asia	1724.34 (1364.10–2142.63)	212.38 (157.97–279.26)	−87.68 (−89.71 to −85.51)	−49.49 (−50.17 to −48.81)
Southeast Asia	4300.84 (3797.58–4793.93)	1727.26 (1465.46–2061.26)	−59.84 (−63.83 to −54.78)	−87.72 (−91.19 to −84.25)
Oceania	3039.81 (2742.84–3358.90)	1942.08 (1684.67–2228.69)	−36.11 (−42.01 to −29.43)	−39.83 (−41.91 to −37.76)
Central Asia	2416.84 (2225.11–2638.90)	259.89 (222.13–301.55)	−89.25 (−90.52 to −87.95)	−68.46 (−69.50 to −67.42)
Central Europe	388.80 (318.60–470.24)	139.56 (112.90–171.80)	−64.10 (−66.60 to −61.91)	−8.00 (−8.30 to −7.71)
Eastern Europe	1899.31 (1553.15–2327.27)	383.97 (295.49–486.54)	−79.78 (−81.81 to −77.67)	−47.23 (−48.20 to −46.25)
High-income Asia Pacific	1040.13 (769.11–1389.91)	1263.44 (952.92–1640.22)	21.47 (10.95–31.13)	7.57 (6.53–8.61)
Australasia	498.27 (375.67–670.19)	171.89 (134.37–223.82)	−65.50 (−68.76 to −62.23)	−9.85 (−10.86 to −8.85)
Western Europe	1264.65 (918.55–1727.32)	1117.42 (825.03–1473.44)	−11.64 (−16.81 to −4.75)	−7.15 (−9.23 to −5.07)
Southern Latin America	1693.70 (1434.36–1983.09)	357.15 (277.40–448.02)	−78.91 (−81.49 to −76.08)	−41.69 (−43.73 to −39.65)
High-income North America	559.59 (388.05–788.05)	52.37 (41.84–65.49)	−90.64 (−92.05 to −88.96)	−18.26 (−20.29 to −16.23)
Caribbean	1801.51 (1612.10–1992.35)	547.47 (466.73–643.56)	−69.61 (−72.13 to −67.00)	−39.81 (−40.79 to −38.83)
Andean Latin America	5405.56 (5039.58–5758.60)	428.94 (353.58–508.88)	−92.06 (−93.29 to −90.55)	−161.67 (−162.87 to −160.48)
Central Latin America	2208.59 (1880.05–2588.08)	474.81 (395.55–565.60)	−78.50 (−80.13 to −76.74)	−56.10 (−58.38 to −53.83)
Tropical Latin America	2814.90 (2335.91–3351.51)	495.70 (388.32–618.03)	−82.39 (−84.07 to −80.85)	−76.90 (−78.57 to −75.23)
North Africa and middle East	3581.24 (3225.41–3957.60)	786.81 (683.37–907.98)	−78.03 (−79.87 to −76.23)	−90.56 (−92.52 to −88.60)
South Asia	3801.35 (3222.38–4445.53)	985.91 (839.65–1163.61)	−74.06 (−76.55 to −71.21)	−91.97 (−94.10 to −89.84)
Central Sub-Saharan Africa	5081.00 (4605.53–5537.31)	1125.27 (972.04–1309.66)	−77.85 (−80.23 to −74.90)	−137.79 (−142.18 to −133.39)
Eastern Sub-Saharan Africa	5027.46 (4575.44–5510.32)	974.18 (866.67–1102.37)	−80.62 (−82.35 to −78.58)	−132.66 (−134.11 to −131.20)
Southern Sub-Saharan Africa	4596.87 (3988.59–5179.94)	895.13 (771.03–1037.96)	−80.53 (−82.82 to −77.73)	−122.28 (−124.15 to −120.41)
Western Sub-Saharan Africa	5262.77 (4744.19–5735.65)	1277.72 (1119.73–1472.29)	−75.72 (−78.25 to −72.55)	−129.52 (−131.52 to −127.53)
High-middle SDI region	1659.29 (1366.30–2012.20)	423.52 (334.16–530.67)	−74.48 (−77.06 to −71.95)	−39.91 (−40.54 to −39.27)
High SDI region	939.43 (708.39–1250.85)	608.41 (456.01–799.00)	−35.24 (−39.71 to −31.09)	−10.91 (−12.41 to −9.42)
Low-middle SDI region	4144.11 (3663.65–4673.73)	1032.66 (899.96–1189.28)	−75.08 (−76.88 to −73.06)	−103.49 (−105.57 to −101.42)
Low SDI region	4752.56 (4299.25–5224.10)	1157.71 (1014.17–1325.92)	−75.64 (−77.63 to −73.17)	−115.73 (−116.98 to −114.48)
Middle SDI region	2903.06 (2479.77–3362.19)	736.45 (620.83–875.57)	−74.63 (−76.16 to −73.08)	−67.39 (−68.78 to −66.00)

Abbreviations: GBD, Global Burden of Disease; AAPC, average annual percent change; *CI*, confidence interval; *UI*, uncertainty interval; SDI, socio-demographic index.

The mortality rate of diarrhea in children under five fell from 263.95 per 100,000 population in 1990 (95% *UI*: 207.34–311.47 per 100,000 population) to 51.72 per 100,000 population in 2021 (95% *UI*: 38.13–70.54 per 100,000 population), reflecting a significant decline (AAPC = −6.89, 95% *CI*: −6.95 to −6.83) (Table 3). The number of deaths decreased from 1.64 million in 1990 (95% *UI*: 1.29–1.93 million) to 0.34 million in 2021 (95% *UI*: 0.25–0.46 million), showing a consistent downward trend (Table S3).

**Table 3:** The mortality rate of diarrheal disease among children under five years in 1990 and 2021, and the changing trend of the mortality rate across different GBD regions.

Location	Mortality rate per 100,000 population (95% *UI*) in 1990	Mortality rate per 100,000 population (95% *UI*) in 2021	Percentage change of mortality rate (95% *UI*) in 1990–2021/%	AAPC of mortality rate (95% *CI*) in 1990–2021/%
Global	263.95 (207.34–311.47)	51.72 (38.13–70.54)	−80.40 (−85.53 to −74.04)	−6.89 (−6.95 to −6.83)
East Asia	62.68 (46.37–79.32)	0.99 (0.75–1.36)	−98.42 (−98.85 to −97.85)	−2.01 (−2.05 to −1.96)
Southeast Asia	292.38 (182.00–379.88)	24.34 (18.27–31.88)	−91.68 (−94.09 to −86.71)	−8.63 (−8.79 to −8.47)
Oceania	191.18 (133.17–280.73)	91.31 (55.02–142.36)	−52.24 (−68.61 to −30.03)	−3.29 (−3.48 to −3.09)
Central Asia	143.58 (129.43–160.05)	17.17 (12.41–23.07)	−88.04 (−91.60 to −83.93)	−4.20 (−4.36 to −4.04)
Central Europe	8.46 (7.53–9.51)	3.31 (2.70–3.90)	−60.81 (−68.35 to −52.61)	−0.15 (−0.16 to −0.14)
Eastern Europe	8.04 (7.62–8.48)	0.90 (0.80–1.00)	−88.79 (−90.02 to −87.57)	−0.22 (−0.24 to −0.20)
High-income Asia Pacific	1.69 (1.41–2.12)	0.66 (0.58–0.75)	−60.87 (−68.89 to −50.71)	−0.03 (−0.03 to −0.03)
Australasia	0.89 (0.78–1.01)	0.33 (0.27–0.41)	−62.68 (−71.23 to −52.29)	−0.02 (−0.02 to −0.01)
Western Europe	0.73 (0.67–0.80)	0.63 (0.53–0.73)	−14.15 (−28.08 – 1.33)	−0.00 (−0.01 to −0.00)
Southern Latin America	16.71 (15.34–18.20)	2.07 (1.63–2.64)	−87.60 (−90.39 to −83.86)	−0.47 (−0.51 to −0.44)
High-income North America	1.03 (0.97–1.10)	0.57 (0.49–0.65)	−45.15 (−53.05 to −37.00)	−0.01 (−0.02 to −0.01)
Caribbean	303.39 (248.83–360.48)	102.75 (68.75–142.91)	−66.13 (−77.85 to −53.29)	−7.00 (−8.10 to −5.90)
Andean Latin America	149.07 (124.59–178.45)	9.16 (5.99–12.99)	−93.86 (−95.92 to −91.48)	−5.21 (−5.59 to −4.84)
Central Latin America	172.73 (159.29–188.61)	14.45 (10.50–19.28)	−91.64 (−94.03 to −88.71)	−4.95 (−5.09 to −4.81)
Tropical Latin America	184.75 (157.68–212.97)	4.35 (3.40–5.50)	−97.65 (−98.21 to −96.93)	−5.88 (−5.97 to −5.79)
North Africa and middle East	176.43 (132.04–226.29)	18.77 (13.22–28.93)	−89.36 (−92.17 to −85.78)	−5.04 (−5.11 to −4.97)
South Asia	390.69 (311.63–469.33)	35.41 (21.76–51.39)	−90.94 (−94.54 to −86.77)	−11.67 (−11.86 to −11.48)
Central Sub-Saharan Africa	583.73 (406.19–736.64)	63.63 (39.01–98.54)	−89.10 (−92.81 to −82.07)	−16.57 (−17.02 to −16.11)
Eastern Sub-Saharan Africa	566.66 (357.13–776.49)	104.50 (72.49–151.08)	−81.56 (−88.54 to −69.62)	−15.14 (−15.42 to −14.85)
Southern Sub-Saharan Africa	316.37 (273.20–364.18)	100.89 (74.14–134.47)	−68.11 (−75.32 to −59.32)	−7.42 (−7.90 to −6.95)
Western Sub-Saharan Africa	810.81 (566.21–1007.66)	197.35 (132.88–287.80)	−75.66 (−82.55 to −65.46)	−20.36 (−20.76 to −19.96)
High-middle SDI region	35.61 (28.39–42.82)	2.40 (1.86–3.00)	−93.27 (−94.67 to −91.63)	−1.07 (−1.09 to −1.05)
High SDI region	3.39 (2.62–4.57)	0.79 (0.68–0.89)	−76.71 (−82.78 to −69.55)	−0.08 (−0.09 to −0.08)
Low-middle SDI region	426.17 (343.75–497.47)	44.63 (33.79–59.80)	−89.53 (−92.28 to −85.54)	−12.42 (−12.50 to −12.34)
Low SDI region	630.33 (459.39–790.35)	137.15 (96.81–190.65)	−78.24 (−84.66 to −67.94)	−16.30 (−16.57 to −16.03)
Middle SDI region	143.86 (110.85–170.60)	14.40 (11.00–18.93)	−89.99 (−92.05 to −87.70)	−4.18 (−4.24 to −4.12)

Abbreviations: GBD, Global Burden of Disease; AAPC, average annual percent change; *CI*, confidence interval; *UI*, uncertainty interval; SDI, socio-demographic index.

The DALY rate due to diarrhea in children under five years dropped from 23,838.68 per 100,000 population in 1990 (95% *UI*: 18,844.31–28,015.78 per 100,000 population) to 4699.58 per 100,000 population in 2021 (95% *UI*: 3512.49–6376.29 per 100,000 population), indicating a significant reduction (AAPC = −621.79, 95% *CI*: −627.20 to −616.38) (Table 4). Correspondingly, the number of DALYs decreased from 147.79 million in 1990 (95% *UI*: 116.82–173.68 million) to 30.93 million in 2021 (95% *UI*: 23.12–41.97 million), showing a marked decline (Table S4).

**Table 4:** The DALY rate of diarrheal disease among children under five years in 1990 and 2021, and the changing trend of the DALY rate across different GBD regions.

Location	DALY rate per 100,000 population (95% *UI*) in 1990	DALY rate per 100,000 population (95% *UI*) in 2021	Percentage change of DALY rate (95% *UI*) in 1990–2021/%	AAPC of DALY rate (95% *CI*) in 1990–2021/%
Global	23,838.68 (18,844.31–28,015.78)	4699.58 (3512.49–6376.29)	−80.29 (−85.31 to −74.05)	−621.79 (−627.20 to −616.38)
East Asia	5773.92 (4343.03–7250.92)	113.43 (88.33–146.69)	−98.04 (−98.55 to −97.41)	−182.65 (−186.59 to −178.71)
Southeast Asia	26,528.93 (16,790.15–34,260.42)	2369.54 (1810.56–3036.72)	−91.07 (−93.54 to −86.03)	−775.81 (−790.43 to −761.20)
Oceania	17,364.81 (12,219.49–25,393.35)	8346.77 (5149.29–12,916.15)	−51.93 (−67.92 to −30.27)	−297.12 (−314.42 to −279.83)
Central Asia	13,085.32 (11,796.98–14,569.95)	1560.82 (1130.19–2084.09)	−88.07 (−91.56 to −84.07)	−380.85 (−395.97 to −365.73)
Central Europe	801.57 (719.75–889.34)	312.79 (259.04–365.82)	−60.98 (−68.12 to −53.19)	−14.36 (−15.56 to −13.17)
Eastern Europe	938.96 (853.08–1040.35)	125.33 (108.31–146.80)	−86.65 (−87.88 to −85.30)	−25.19 (−27.12 to −23.26)
High-income Asia Pacific	272.31 (218.13–347.37)	206.46 (151.52–289.02)	−24.18 (−37.59 to −11.91)	−1.95 (−2.14 to −1.76)
Australasia	137.34 (112.21–170.14)	49.68 (40.50–62.37)	−63.83 (−69.43 to −57.52)	−2.44 (−2.72 to −2.16)
Western Europe	213.08 (155.79–299.23)	186.70 (139.28–255.69)	−12.38 (−18.24 to −5.92)	−0.91 (−1.17 to −0.66)
Southern Latin America	1689.92 (1545.58–1834.86)	226.52 (182.24–284.02)	−86.60 (−89.22 to −83.26)	−47.27 (−50.37 to −44.17)
High-income North America	158.08 (132.02–196.26)	56.86 (49.47–64.72)	−64.03 (−71.06 to −56.84)	−2.60 (−2.93 to −2.27)
Caribbean	27,250.03 (22,428.55–32,314.72)	9223.41 (6206.31–12,811.86)	−66.15 (−77.81 to −53.38)	−617.10 (−708.91 to −525.29)
Andean Latin America	13,878.76 (11,671.16–16,409.75)	864.33 (585.62–1205.73)	−93.77 (−95.75 to −91.49)	−481.71 (−515.64 to −447.77)
Central Latin America	15,646.98 (14,444.86–17,085.79)	1341.72 (988.75–1779.40)	−91.43 (−93.80 to −88.54)	−447.65 (−459.90 to −435.40)
Tropical Latin America	16,850.51 (14,389.96–19,365.06)	445.65 (359.78–547.28)	−97.36 (−97.96 to −96.61)	−534.89 (−542.59 to −527.19)
North Africa and middle East	16,145.92 (12,173.72–20,601.85)	1765.15 (1280.13–2658.66)	−89.07 (−91.82 to −85.63)	−458.07 (−464.41 to −451.73)
South Asia	35,198.65 (28,119.06–42,176.67)	3267.38 (2066.25–4691.40)	−90.72 (−94.25 to −86.59)	−1059.87 (−1073.45 to −1046.28)
Central Sub-Saharan Africa	52,503.20 (36,619.49–66,048.00)	5792.12 (3593.83–8926.64)	−88.97 (−92.65 to −82.04)	−1484.40 (−1527.11 to −1441.69)
Eastern Sub-Saharan Africa	50,923.68 (32,335.09–69,517.88)	9397.03 (6544.95–13,553.68)	−81.55 (−88.49 to −69.79)	−1360.55 (−1387.67 to −1333.43)
Southern Sub-Saharan Africa	28,744.38 (24,826.34–33,039.04)	9097.62 (6720.75–12,075.27)	−68.35 (−75.43 to −59.79)	−676.66 (−719.40 to −633.92)
Western Sub-Saharan Africa	72,528.16 (50,849.28–89,903.72)	17,660.54 (11,989.31–25,664.69)	−75.65 (−82.50 to −65.57)	−1821.49 (−1857.50 to −1785.47)
High-middle SDI region	3363.08 (2698.52–3993.35)	263.00 (209.92–317.77)	−92.18 (−93.64 to −90.41)	−99.71 (−101.50 to −97.91)
High SDI region	411.72 (327.99–521.11)	141.50 (114.58–177.12)	−65.63 (−73.97 to −57.68)	−8.53 (−8.73 to −8.32)
Low-middle SDI region	38,408.45 (31,009.23–44,777.94)	4094.07 (3122.24–5451.19)	−89.34 (−92.05 to −85.39)	−1117.08 (−1124.50 to −1109.66)
Low SDI region	56,518.92 (41,443.09–70,762.92)	12,309.84 (8752.12–17,028.51)	−78.22 (−84.56 to −68.10)	−1460.37 (−1482.60 to −1438.14)
Middle SDI region	13,147.99 (10,248.57–15,528.01)	1368.86 (1065.51–1754.18)	−89.59 (−91.57 to −87.38)	−378.62 (−384.03 to −373.20)

Abbreviations: DALY, disability-adjusted life year; GBD, Global Burden of Disease; AAPC, average annual percent change; *CI*, confidence interval; *UI*, uncertainty interval; SDI, socio-demographic index.

### 3.2 SDI regions

In 2021, the incidence rate of diarrhea among children under five years was highest in the low SDI region, at 79,236.37 per 100,000 population (95% *UI*: 66,678.62–92,048.61 per 100,000 population). From 1990 to 2021, the incidence rate showed a significant decline across all five SDI regions, with the smallest reduction observed in the high SDI region (AAPC = −699.24, 95% *CI*: −791.83 to −606.64) and the largest in the low SDI region (AAPC = −6591.68, 95% *CI*: −6706.93 to −6476.43) (Table 1, Fig. S1).

Similarly, the prevalence rate of diarrhea in 2021 was highest in the low SDI region, at 1157.71 per 100,000 population (95% *UI*: 1014.17–1325.92 per 100,000 population). Between 1990 and 2021, the prevalence rate significantly decreased across all SDI regions, with the smallest reduction in the high SDI region (AAPC = −10.91, 95% *CI*: −12.41 to −9.42) and the largest in the low SDI region (AAPC = −115.73, 95% *CI*: −116.98 to −114.48) (Table 2, Fig. S1).

The mortality rate of diarrhea among children under five in 2021 was also highest in the low SDI region, at 137.15 per 100,000 population (95% *UI*: 96.81–190.65 per 100,000 population). From 1990 to 2021, the mortality rate declined in all SDI regions, with the smallest reduction in the high SDI region (AAPC = −0.08, 95% *CI*: −0.09 to −0.08) and the largest in the low SDI region (AAPC = −16.30, 95% *CI*: −16.57 to −16.03) (Table 3, Fig. S1).

The DALY rate for diarrhea in children under five in 2021 was highest in the low SDI region, at 12,309.84 per 100,000 population (95% *UI*: 8752.12–17,028.51 per 100,000 population). Between 1990 and 2021, the DALY rate declined across all SDI regions, with the smallest decrease observed in the high SDI region (AAPC = −8.53, 95% *CI*: −8.73 to −8.32) and the largest in the low SDI region (AAPC = −1460.37, 95% *CI*: −1482.60 to −1438.14) (Table 4, Fig. S1).

### 3.3 Geographical regions

In 2021, the incidence rate of diarrhea among children under five years was highest in Oceania (128,542.07 per 100,000 population, 95% *UI*: 107,231.53–150,654.43 per 100,000 population) and lowest in high-income North America (3133.27 per 100,000 population, 95% *UI*: 2437.97–3988.19 per 100,000 population). Between 1990 and 2021, the incidence rate of diarrheal diseases decreased in 20 out of the 21 regions, with the exception of high-income Asia Pacific, where it increased (AAPC = 561.60, 95% *CI*: 498.80–624.41). The largest decrease was observed in Andean Latin America (AAPC = −8789.01, 95% *CI*: −8902.36 to −8675.65) (Table 1).

In 2021, the prevalence rate of diarrhea for children under five years was also highest in Oceania (1942.08 per 100,000 population, 95% *UI*: 1684.67 to 2228.69 per 100,000 population) and lowest in high-income North America (1263.44 per 100,000 population, 95% *UI*: 952.92–1640.22 per 100,000 population). From 1990 to 2021, the prevalence rate of diarrhea in children under five years declined in all regions except high-income Asia Pacific, where it increased (AAPC = 7.57, 95 % *CI*: 6.53–8.61). The largest reduction occurred in Andean Latin America (AAPC = −161.67, 95% *CI*: −162.87 to −160.48) (Table 2).

In 2021, the mortality rate due to diarrhea in children under five years was highest in Western Sub-Saharan Africa (197.35 per 100,000 population, 95% *UI*: 132.88–287.80 per 100,000 population) and lowest in Australasia (0.33 per 100,000 population, 95% *UI*: 0.27–0.41 per 100,000 population). Between 1990 and 2021, the mortality rate decreased in all 21 regions, with the greatest decline seen in Western Sub-Saharan Africa (AAPC = −20.36, 95% *CI*: −20.76 to −19.96) (Table 3).

The DALY rate for diarrhea in children under five years in 2021 was highest in Western Sub-Saharan Africa (17,660.54 per 100,000 population, 95% *UI*: 11,989.31–25,664.69 per 100,000 population) and lowest in Australasia (49.68 per 100,000 population, 95% *UI*: 40.50–62.37 per 100,000 population). From 1990 to 2021, the DALY rate declined in all regions, with Western Sub-Saharan Africa showing the largest decrease (AAPC = −1821.49, 95% *CI*: −1857.50 to −1785.47) (Table 4).

### 3.4 Countries and territories

In 2021, the highest incidence rate of diarrhea in children under five years was observed in the Netherlands, at 166,208.95 per 100,000 population (95% *UI*: 111,931.07–220,944.04 per 100,000 population). The highest number of cases occurred in Cambodia at 1.23 million cases (95% *UI*: 1.04–1.42 million). Among 204 countries and territories, the incidence rate of diarrhea increased in 11 countries, remained relatively stable in two, and decreased in 191. The largest increase was seen in Taiwan, China (AAPC = 2124.30, 95% *CI*: 2026.29–2222.31), while Paraguay experienced the largest decrease (AAPC = −10,077.81, 95% *CI*: −10,215.92 to −9939.70) (Table S5, Fig. S1).

In 2021, the highest prevalence rate of diarrhea was also reported in the Netherlands, at 2622.19 per 100,000 population (95% *UI*: 1800.27–3559.28 per 100,000 population). India had the highest number of prevalent cases: 1.15 million cases (95% *UI*: 1.00–1.37 million). Across the 204 countries and territories, the prevalence rate increased in nine countries, remained stable in six, and decreased in 189. The largest increase in prevalence rate was in the Netherlands (AAPC = 28.15, 95% *CI*: 21.96–34.33), while Paraguay had the greatest decline (AAPC = −187.35, 95% *CI*: −189.36 to −185.35) (Table S6, Fig. S2).

In terms of mortality rate in 2021, Chad had the highest diarrhea-related mortality rate, at 560.45 per 100,000 population (95% *UI*: 373.77–952.59 per 100,000 population), while Nigeria had the highest number of deaths: 97.68 thousand (95% *UI*: 63.51–141.99 thousand). Mortality rates increased in 11 countries, remained stable in 13, and declined in 180 countries. The largest increase in mortality was observed in Tokelau (AAPC = 1.82, 95% *CI*: 1.42–2.22), while Niger showed the greatest decline (AAPC = −40.09, 95% *CI*: −41.01 to −39.18) (Table S7, Fig. S2).

Chad had the highest DALY rate for diarrhea in 2021: 49,921.52 per 100,000 population (95% *UI*: 33,399.80–84,699.30 per 100,000 population). Nigeria had the highest number of DALYs: 8.74 million (95% *UI*: 5.71–12.65 million). Among the 204 countries and territories, DALY rates increased in 7 countries, remained stable in 11, and decreased in 186. The largest increase in DALY rates was recorded in Tokelau (AAPC = 159.74, 95% *CI*: 123.56–195.91), while Niger saw the largest decrease (AAPC = −3552.02, 95% *CI*: −3635.36 to −3468.68) (Table S8, Fig. S2).

### 3.5 Global trends by age-gender

In 2021, there were no significant differences in the incidence, prevalence, mortality, or DALY rates of diarrheal disease between males and females across age groups <1 year, 1–2 years, 2–4 years, and <5 years. In addition, the number of male and female children affected by diarrhea showed no significant differences across these age groups (Fig. S3A–D).

### 3.6 The association between rate and SDI

In 2021, there was a negative correlation between the SDI and the rates of diarrheal disease in children under five years across 204 countries and territories. Specifically, incidence rate (*r* = −0.204, *P* < 0.001), prevalence rate (*r* = −0.193, *P* < 0.001), mortality rate (*r* = −0.879, *P* < 0.001), and DALY rate (*r* = −0.855, *P* < 0.001) were all negatively correlated with SDI (Fig. S4A–D).

From 1990 to 2021, a similar negative correlation was observed globally between SDI and diarrheal disease rates in children under five. Incidence (*r* = −0.782, *P* < 0.001), prevalence (*r* = −0.777, *P* < 0.001), mortality (*r* = −0.908, *P* < 0.001), and DALY rates (*r* = −0.904, *P* < 0.001) were all negatively associated with SDI over this period (Fig. S5A–D).

### 3.7 Risk factors

**Fig. 1:**
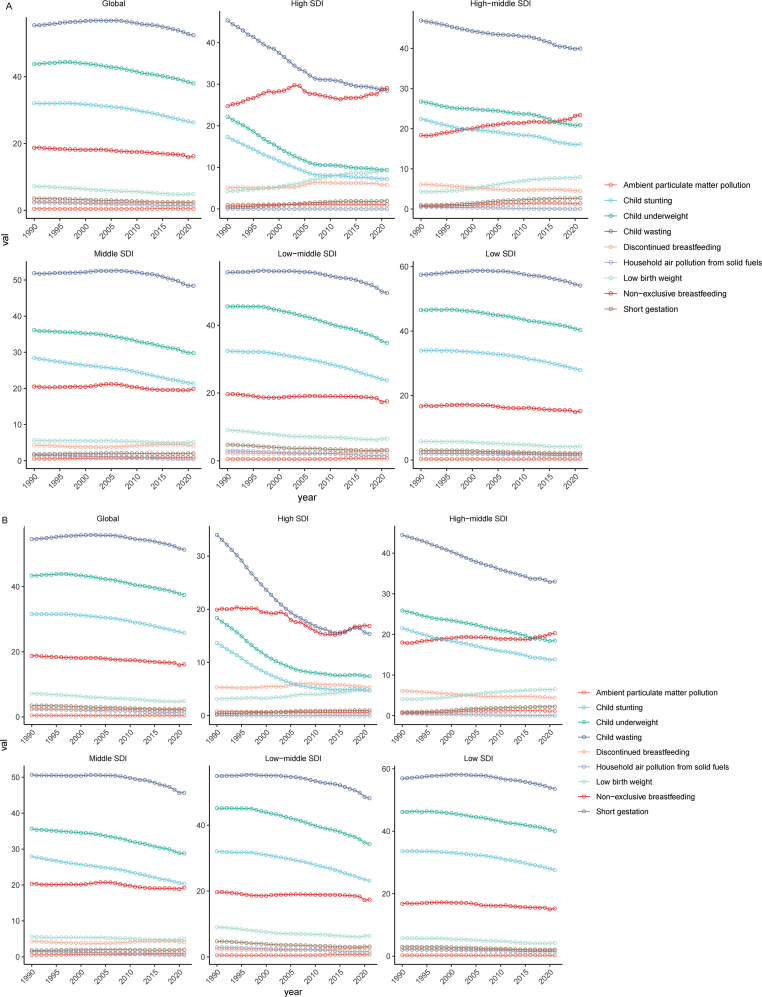
The changing trend of risk factors leading to mortality rate and DALY rate of children under five years old with diarrheal disease from 1990 to 2021. A: mortality rate. B: DALY rate. Abbreviations: DALYs, disability-adjusted life years; SDI, socio-demographic index.

At the global level, from 1990 to 2021, child wasting consistently remained the leading cause of diarrhea-related deaths among children under five, followed by child underweight, child stunting and non-exclusive breastfeeding. The contribution of these four factors to diarrhea-related mortality has been gradually declining. Similar trends were observed in middle SDI, low-middle SDI, and low SDI regions. In contrast, in high SDI regions, non-exclusive breastfeeding emerged as the second leading cause of diarrhea-related deaths in children under five years, with its contribution steadily increasing from 1990 to 2021. In high-middle SDI regions, deaths attributed to non-exclusive breastfeeding have risen consistently and, since 2015, it has become the second most significant cause of diarrhea-related mortality in children under five years (Fig. 1A).

At the global level, from 1990 to 2021, the primary risk factors contributing to diarrhea-related DALY rates among children under five years were child wasting, child underweight, child stunting and non-exclusive breastfeeding, with the contribution of these factors gradually decreasing over time. Notably, non-exclusive breastfeeding became the leading risk factor after 2020 in high SDI regions, it has ranked second since 2018 and continues to show a slow upward trend in high-middle SDI regions. In additions, the ranking and trends of major risk factors align with global patterns in middle SDI, low-middle SDI, and low SDI regions (Fig. 1B).

### 3.8 Projecting

**Fig. 2:**
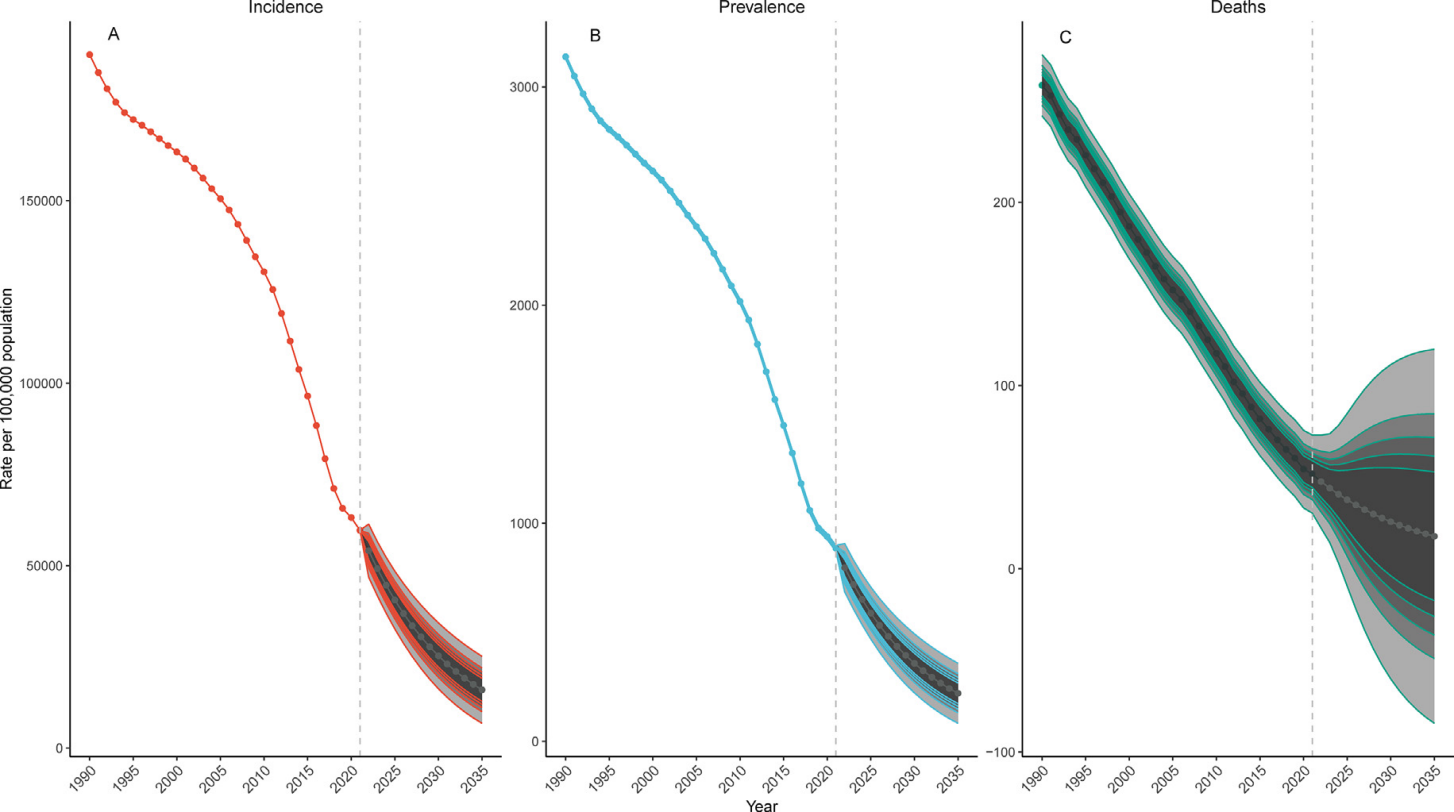
The prediction of global burden of diarrheal diseases among children under five years for 2022–2035 based on the Bayesian age-period-cohort model. A: incidence rate. B: prevalence rate. C: mortality rate.

The study, using the BACP model, projects a significant decline in the incidence rate, prevalence rate, and mortality rate of diarrhea among children under five years from 2022 to 2035. Globally, the incidence rate in 2035 is expected to be 15,974.66 per 100,000 population (95% *CI*: 6801.97–25,147.36 per 100,000 population). The EAPC for the incidence rate over this period is −8.97% (95% *CI*: −9.02% to −8.92%). The prevalence rate in 2035 is projected to be 220.74 per 100,000 population (95% *CI*: 83.30–358.19 per 100,000 population), with an EAPC of −9.41% (95% *CI*: −9.46% to −9.36%). The mortality rate for 2035 is expected to be relatively low, at 17.77 per 100,000 population (95% *CI*: 0.00–118.29 per 100,000 population), and the EAPC for mortality is projected at −7.32% (95% *CI*: −7.37% to −7.26%) (Fig. 2, Table 5).

**Table 5:** The prediction of global burden of diarrheal disease among children under five years for 2022–2035 based on the BAPC model.

Disease	Index	Incidence	Prevalence	Death
Diarrheal disease (<5 years)	Rate per 100,000 population (95% *CI*) in 2035	15,974.66 (6801.97–25,147.36)	220.74 (83.3–358.19)	17.77 (0.00–118.29)
	EAPC (95% *CI*) in 2022–2035/%	−8.97 (−9.02 to −8.92)	−9.41 (−9.46 to −9.36)	−7.32 (−7.37 to −7.26)

Abbreviations: BAPC, Bayesian age-period-cohort; *CI*, confidence interval; EAPC, estimated annual percentage change.

### 3.9 Global trends in pathogens

**Fig. 3:**
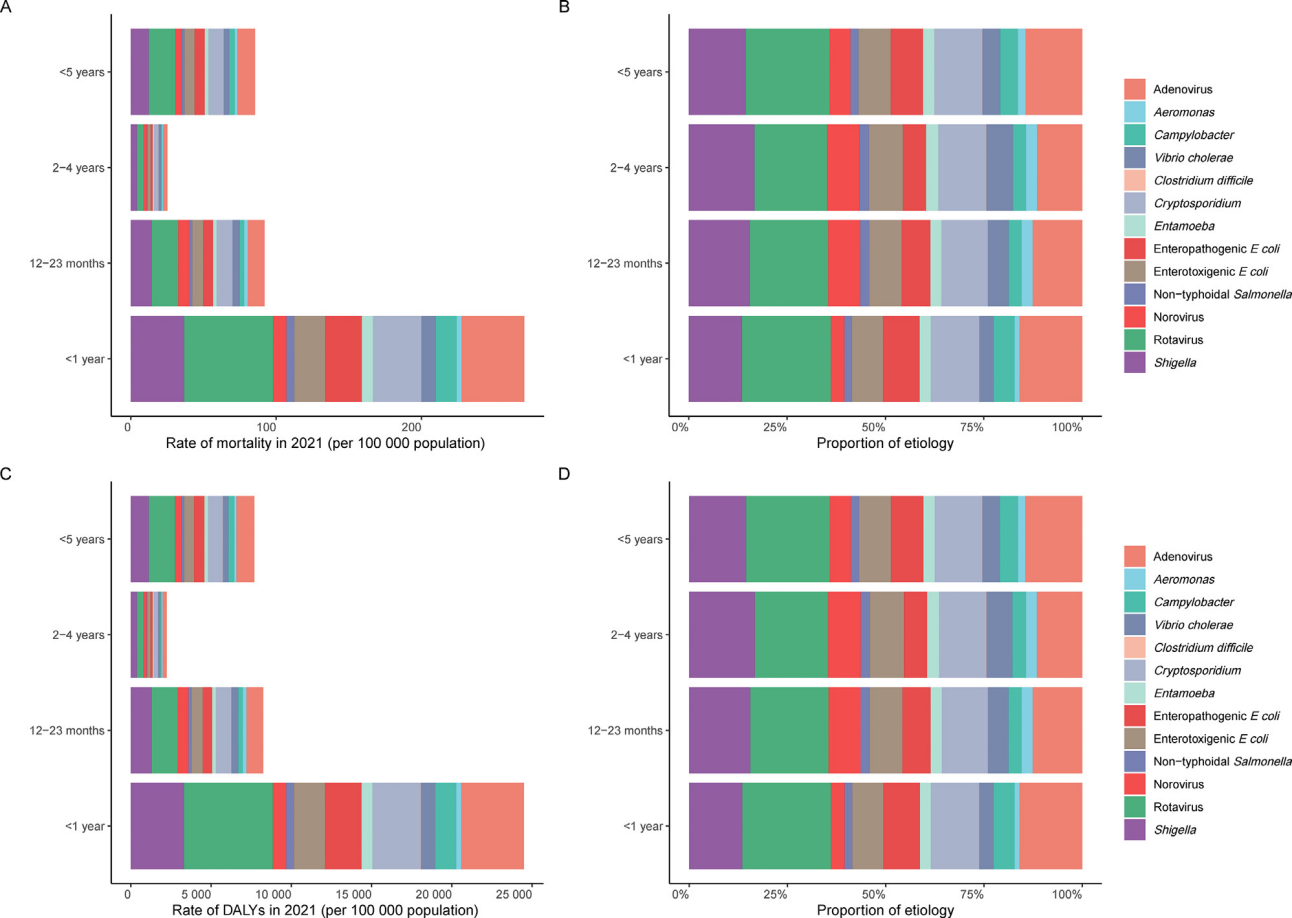
The diarrhea-related mortality and DALY in children under five years of age attributed to specific pathogens globally in 2021 year. A: mortality rate. B: mortality number. C: DALY rate. D: DALY number. Abbreviations: DALY, disability-adjusted life year.

In 2021, rotavirus was the leading pathogen responsible for diarrhea-related deaths rate among children under five years globally, across all age groups, followed by *Shigella* and adenovirus (Fig. 3A). From 1990 to 2021, among 13 common diarrhea-related pathogens, mortality rate due to all but *C. difficile* decreased in children under five across all age groups (Table 6, Fig. 3A). In addition, in 2021, rotavirus accounted for the highest number of deaths in children under five years across all age groups. While the number of mortality cases due to *C. difficile* increased in children aged <5 years, 1–2 years, and 2–4 years from 1990 to 2021, it showed a declining trend in infants under 1 year. Apart from *C. difficile*, the number of mortality cases caused by the other 12 pathogens continued to decline over this period (Table S9, Fig. 3B).

**Table 6:** The changing trend of mortality rate of children under five years old attributed to specific diarrheal pathogens across the globe from 1990 to 2021.

Age group	Pathogen	Mortality rate per 100,000 population (95% *UI*) in 1990	Mortality rate per 100,000population (95% *UI*) in 2021	AAPC of mortality rate (95% *CI*) in 1990–2021/%
<5 years	*Vibrio cholerae*	17.76 (14.75–21.43)	3.92 (2.81–5.31)	−0.47 (−0.49 to −0.45)
	Non-typhoidal *Salmonella*	8.16 (1.20–17.77)	1.8 (0.24–4.17)	−0.21 (−0.21 to −0.20)
	*Shigella*	48.35 (29.24–78.08)	12.42 (7.28–20.96)	−1.19 (−1.20 to −1.18)
	Enteropathogenic *Escherichia coli*	26.83 (15.46–42.14)	7.04 (3.95–11.59)	−0.32 (−0.32 to −0.31)
	Enterotoxigenic *Escherichia coli*	29.72 (17.32–47.09)	6.96 (3.83–11.72)	−0.67 (−0.68 to −0.66)
	*Campylobacter*	22.98 (10.17–42.4)	3.84 (1.56–7.41)	−0.64 (−0.65 to −0.63)
	*Entamoeba*	12.28 (5.88–23.03)	2.44 (1.07–4.91)	−0.32 (−0.32 to −0.31)
	*Cryptosporidium*	38.32 (23.65–59.95)	10.4 (5.91–16.65)	−0.90 (−0.93 to −0.87)
	Rotavirus	100.31 (74.21–127.24)	18.21 (12.63–25.74)	−2.62 (−2.66 to −2.58)
	*Aeromonas*	8.31 (3.62–15.15)	1.63 (0.73–3.07)	−0.22 (−0.22 to −0.22)
	*Clostridium difficile*	0.02 (0.01–0.03)	0.02 (0.01–0.03)	−0.00 (−0.00 – 0.00)
	Norovirus	22.35 (7.64–40.06)	4.49 (1.47–8.36)	−0.58 (−0.58 to −0.57)
	Adenovirus	56.78 (32.7–90.28)	12.32 (6.82–20.21)	−1.47 (−1.49 to −1.44)
<1 years	*Vibrio cholerae*	43.56 (34.48–53.92)	9.9 (6.66–13.81)	−1.17 (−1.22 to −1.12)
	Non-typhoidal *Salmonella*	23.1 (3.87–51.69)	5.27 (0.87–12.21)	−0.58 (−0.59 to −0.57)
	*Shigella*	134.35 (75.91–227.36)	36.67 (19.98–64.09)	−3.29 (−3.35 to −3.22)
	Enteropathogenic *Escherichia coli*	90.1 (47.49–152.77)	25.17 (12.41–44.49)	−2.23 (−2.28 to −2.17)
	Enterotoxigenic *Escherichia coli*	89.15 (49.43–150.83)	21.45 (11.09–36.92)	−2.22 (−2.25 to −2.20)
	*Campylobacter*	80.75 (33.33–153.56)	14.36 (5.6–28.61)	−2.21 (−2.25 to −2.17)
	*Entamoeba*	35.36 (15.94–69.56)	7.57 (3.12–15.31)	−0.91 (−0.92 to −0.90)
	*Cryptosporidium*	113.18 (67.77–180.94)	33.37 (18.9–54.53)	−2.63 (−2.69 to −2.57)
	Rotavirus	309.15 (230.49–390.99)	61.26 (42.73–85.95)	−8.05 (−8.13 to −7.97)
	*Aeromonas*	17.05 (4.23–40.96)	3.54 (0.89–8.95)	−0.45 (−0.45 to −0.44)
	*Clostridium difficile*	0.02 (0.01–0.04)	0.02 (0.01–0.04)	−0.00 (−0.00 to −0.00)
	Norovirus	42.75 (14.17–89.68)	9.2 (3.02–19.17)	−1.11 (−1.12 to −1.10)
	Adenovirus	182.36 (104.36–288.12)	43.1 (24.47–71.98)	−4.71 (−4.79 to −4.63)
1–2 years	*Vibrio cholerae*	22.05 (18.04–27.06)	4.93 (3.41–6.95)	−0.58 (−0.60 to −0.56)
	*Non-typhoidal Salmonella*	9.21 (0.00–26.42)	2.14 (0.00–6.5)	−0.23 (−0.23 to −0.23)
	*Shigella*	53.2 (27.47–88.29)	14.32 (7.17–24.46)	−1.28 (−1.29 to −1.26)
	Enteropathogenic *Escherichia coli*	24.28 (8.61–46.29)	6.65 (2.51–12.62)	−0.59 (−0.61 to −0.58)
	Enterotoxigenic *Escherichia coli*	30.13 (14.72–54.09)	7.64 (3.64–15.01)	−0.74 (−0.75 to −0.72)
	*Campylobacter*	16.31 (2.68–41.62)	2.96 (0.48–7.19)	−0.44 (−0.45 to −0.44)
	*Entamoeba*	12.75 (5.45–25.13)	2.62 (1.09–5.4)	−0.32 (−0.33 to −0.32)
	*Cryptosporidium*	39.91 (18.32–73.82)	10.81 (4.95–20.12)	−0.94 (−0.97 to −0.92)
	Rotavirus	97.6 (67.12–134.72)	18.33 (11.56–27.33)	−2.56 (−2.60 to −2.52)
	*Aeromonas*	12.76 (4.86–25.52)	2.62 (0.96–5.38)	−0.33 (−0.33 to −0.33)
	*Clostridium difficile*	0.02 (0.01–0.03)	0.02 (0.01–0.04)	0.00 (−0.00 – 0.00)
	Norovirus	35.41 (5.09–66.56)	7.47 (1.09–14.23)	−0.91 (−0.91 to −0.90)
	Adenovirus	51.01 (24.04–96.47)	11.59 (5.39–20.71)	−1.29 (−1.33 to −1.26)
2–4 years	*Vibrio cholerae*	7.34 (5.59–9.34)	1.71 (1.11–2.45)	−0.18 (−0.19 to −0.18)
	Non-typhoidal *Salmonella*	2.61 (0.00–7.64)	0.6 (0.00–1.87)	−0.06 (−0.06 to −0.06)
	*Shigella*	16.82 (7.91–28.91)	4.2 (2.00–7.96)	−0.41 (−0.41 to −0.40)
	Enteropathogenic *E coli*	5.7 (1.85–11.34)	1.47 (0.49–2.98)	−0.14 (−0.14 to −0.14)
	Enterotoxigenic *E coli*	8.93 (4.34–15.47)	2.18 (1.00–4.13)	−0.22 (−0.22 to −0.22)
	*Campylobacter*	5.16 (0.85–13.21)	0.82 (0.13–2.05)	−0.14 (−0.14 to −0.14)
	*Entamoeba*	4.11 (1.87–8.12)	0.78 (0.33–1.61)	−0.11 (−0.11 to −0.11)
	*Cryptosporidium*	11.76 (5.81–20.97)	3.05 (1.38–5.61)	−0.28 (−0.28 to −0.27)
	Rotavirus	28.64 (18.74–39.96)	4.64 (2.74–7.36)	−0.78 (−0.79 to −0.76)
	*Aeromonas*	3.76 (1.52–7.14)	0.71 (0.28–1.38)	−0.10 (−0.10 to −0.10)
	*Clostridium difficile*	0.01 (0.01–0.02)	0.01 (0.01–0.02)	0.00 (0.00–0.00)
	Norovirus	10.83 (1.59–20.77)	2.06 (0.28–4.12)	−0.28 (−0.28 to −0.28)
	Adenovirus	15.09 (7.91–28.6)	2.88 (1.34–5.68)	−0.39 (−0.40 to −0.38)

Abbreviations: AAPC, average annual percent change; *CI*, confidence interval; *UI*, uncertainty interval.

In 2021, rotavirus was the leading pathogen responsible for diarrhea-related DALY rate among children under five years globally, and across all age groups, followed by *Shigella* and adenovirus (Fig. 3C). From 1990 to 2021, among 13 common diarrhea-related pathogens, DALY rate due to all but *C. difficile* decreased in children under five years across all age groups (Table 7, Fig. 3C).

**Table 7:** The changing trend of DALY rate of children under five years old attributed to specific diarrheal pathogens across the globe from 1990 to 2021.

Age group	Pathogen	DALY rate per 100,000 population (95% *UI*) in 1990	DALY rate per 100,000 population (95% *UI*) in 2021	AAPC of DALY rate (95% *CI*) in 1990–2021/%
<5 years	*Vibrio cholerae*	1575.87 (1308.45–1902.79)	346.9 (248.99–470.04)	−41.66 (−43.11 to −40.21)
	Non-typhoidal *Salmonella*	731 (113.22–1582.89)	161.27 (23.62–371.93)	−18.55 (−18.81 to −18.29)
	*Shigella*	4335.87 (2629.83–6993.84)	1115.88 (656.16–1879.92)	−106.47 (−107.57 to −105.38)
	Enteropathogenic *E coli*	2408.14 (1394.59–3779.64)	633.5 (355.65–1039.61)	−60.15 (−61.62 to −58.68)
	Enterotoxigenic *E coli*	2672.46 (1562.18–4230.62)	627.43 (346.64–1054.51)	−67.02 (−68.26 to −65.78)
	*Campylobacter*	2079.31 (919.26–3814.44)	351.75 (148.74–670.26)	−57.52 (−58.43 to −56.62)
	*Entamoeba*	1098.71 (532.34–2054.85)	218.96 (96.64–438.05)	−28.38 (−28.62 to −28.14)
	*Cryptosporidium*	3424.61 (2116.35–5352.33)	930.43 (531.16–1485.32)	−80.78 (−82.88 to −78.69)
	Rotavirus	9007.42 (6678.68–11,403.73)	1639.23 (1142.33–2309.77)	−244.22 (−247.75 to −240.70)
	*Aeromonas*	741.45 (323.64–1356.03)	145.98 (65.69–274.51)	−19.37 (−19.53 to −19.22)
	*Clostridium difficile*	1.36 (0.78–2.24)	1.33 (0.71–2.31)	−0.00 (−0.00 – 0.00)
	Norovirus	2014.44 (708.88–3584.62)	407.8 (139.96–750.81)	−52.13 (−52.53 to −51.73)
	Adenovirus	5096.4 (2936.66–8125.49)	1111.51 (614.84–1818.75)	−131.36 (−133.93 to −128.79)
<1 years	*Vibrio cholerae*	3904.1 (3090.53–4832.81)	887 (597.18–1237.54)	−105.10 (−109.47 to −100.72)
	Non-typhoidal *Salmonella*	2083.02 (354.11–4655.53)	476.31 (81.05–1099.29)	−52.43 (−53.08 to −51.77)
	*Shigella*	12,097.51 (6852–20,472.58)	3308.52 (1813.52–5770.21)	−296.09 (−302.08 to −290.10)
	Enteropathogenic *E coli*	8118.21 (4289.57–13,763.74)	2274.13 (1125.26–4014.16)	−200.31 (−205.28 to −195.34)
	Enterotoxigenic *E coli*	8036.45 (4454.42–13,589.24)	1941.88 (1004.18–3342.16)	−200.24 (−202.77 to −197.72)
	*Campylobacter*	7310.49 (3044.88–13,859.00)	1313.38 (519.37–2598.01)	−199.77 (−203.14 to −196.40)
	*Entamoeba*	3181.15 (1436.82–6249.22)	681.67 (285.4–1379)	−81.74 (−82.46 to −81.02)
	*Cryptosporidium*	10,181.56 (6102.04–16,277.96)	3005.9 (1704.14–4908.93)	−236.31 (−241.52 to −231.10)
	Rotavirus	27,834.31 (20,775.62–35,185.09)	5525.18 (3856.62–7740.15)	−745.66 (−756.89 to −734.43)
	*Aeromonas*	1539.31 (387.65–3685.93)	321.27 (81.76–806.22)	−40.29 (−40.70 to −39.87)
	*Clostridium difficile*	1.87 (0.98–3.27)	1.66 (0.77–3.16)	−0.01 (−0.01 to −0.00)
	Norovirus	3899.48 (1328.05–8110.4)	848.46 (295.15–1748.08)	−100.61 (−101.60 to −99.61)
	Adenovirus	16,426.59 (9406.54–25,933.84)	3899.63 (2217.01–6494.46)	−419.46 (−430.10 to −408.82)
1–2 years	*Vibrio cholerae*	1954.06 (1599.16–2398.53)	437.05 (302.2–615.47)	−51.20 (−53.09 to −49.31)
	Non-typhoidal *Salmonella*	821.49 (2.27–2346.49)	191.04 (0.52–577.72)	−20.37 (−20.68 to −20.07)
	*Shigella*	4759.44 (2454.2–7910.36)	1281.3 (642.51–2187.36)	−114.27 (−115.49 to −113.06)
	Enteropathogenic *E coli*	2168.71 (777.14–4135.2)	594.2 (225.27–1123.12)	−52.98 (−54.03 to −51.94)
	Enterotoxigenic *E coli*	2704.44 (1325.95–4863.62)	686.68 (327.6–1338.74)	−66.60 (−67.83 to −65.37)
	*Campylobacter*	1470.75 (263.61–3716.06)	268.21 (49.01–642.88)	−39.82 (−40.51 to −39.13)
	*Entamoeba*	1136.25 (490.3–2237.42)	233.62 (98.18–479.35)	−28.97 (−29.26 to −28.67)
	*Cryptosporidium*	3552.89 (1633.35–6563.07)	962.53 (439.94–1791.8)	−83.46 (−85.82 to −81.10)
	Rotavirus	8742.92 (6019.98–12,049.5)	1642.9 (1038.59–2441.12)	−229.16 (−232.89 to −225.44)
	*Aeromonas*	1136.27 (439.8–2274.34)	233.68 (85.83–478.19)	−29.30 (−29.57 to −29.04)
	*Clostridium difficile*	1.75 (1.01–2.88)	1.75 (0.86–3.12)	−0.00 (−0.00 – 0.00)
	Norovirus	3173.34 (490.82–5937.67)	670.36 (105.43–1277.7)	−81.11 (−81.82 to −80.41)
	Adenovirus	4562.01 (2159.96–8573.63)	1039.57 (483.04–1852.35)	−116.00 (−118.42 to −113.57)
2–4 years	*Vibrio cholerae*	638.48 (486.3–812.44)	148.41 (96.52–212.25)	−15.98 (−16.41 to −15.55)
	Non-typhoidal *Salmonella*	230.44 (1.45–667.06)	52.77 (0.4–163.88)	−5.88 (−6.00 to −5.76)
	*Shigella*	1494.76 (701.1–2580.79)	373.97 (177.81–709.31)	−36.19 (−36.63 to −35.75)
	Enteropathogenic *E coli*	504.81 (164.11–996.79)	130.32 (44.25–261.93)	−12.31 (−12.46 to −12.16)
	Enterotoxigenic *E coli*	797.4 (390.65–1371.39)	195.38 (91.98–368.78)	−19.44 (−19.60 to −19.27)
	*Campylobacter*	467.57 (88.59–1171.02)	76.1 (15.97–182.84)	−12.67 (−12.81 to −12.53)
	*Entamoeba*	362.24 (165.81–709.37)	68.85 (29.75–141.16)	−9.41 (−9.51 to −9.31)
	*Cryptosporidium*	1032.78 (511.49–1840.26)	267.83 (122.04–490.1)	−24.37 (−25.16 to −23.59)
	Rotavirus	2553.92 (1685.08–3549.22)	416.6 (250.34–652.14)	−69.01 (−70.24 to −67.79)
	*Aeromonas*	330.3 (134.04–623.28)	62.95 (25.58–121.15)	−8.60 (−8.70 to −8.51)
	*Clostridium difficile*	1.05 (0.54–1.87)	1.1 (0.56–1.94)	0.00 (0.00–0.00)
	Norovirus	966.41 (163.36–1832.82)	185.64 (30.89–361.7)	−24.98 (−25.29 to −24.68)
	Adenovirus	1339.84 (703.97–2532.56)	258.05 (122.32–503.3)	−34.58 (−35.26 to −33.91)

Abbreviations: DALY, disability-adjusted life year; AAPC, average annual percent change; *CI*, confidence interval; *UI*, uncertainty interval.

In addition, in 2021, rotavirus accounted for the highest DALY numbers in children under five years across all age groups. During the period from 1990 to 2021, DALY numbers caused by *C. difficile* increased in children under five years and in the 2–4 years age group, while a declining trend was observed in infants under 1 year and in children aged 1–2 years. Apart from *C. difficile*, DALY numbers associated with the other 12 pathogens continued to decrease during this period (Table S10, Fig. 3D).

## 4 Discussion

The study reveals that, despite a decline in the burden of diarrheal disease among children under five over the past few decades, diarrhea remains a serious health threat, especially in low- and low-middle income regions. Low birth weight and poor child development remain the leading causes of diarrhea-related deaths, with rotavirus still being the primary pathogen. These findings are of significant importance for international organizations, governments, researchers, and healthcare professionals in the effective prevention and control of diarrhea, ultimately reducing the global burden of diarrheal disease.

It highlighted the significant role of various pathogens in the burden of diarrheal disease, particularly the dominance of viral pathogens such as rotavirus in childhood diarrhea. Bacterial pathogens, including *Shigella* and *E. coli*, as well as parasitic infections, have also substantially contributed to the diarrhea burden in specific regions [34,35]. While the mortality and DALYs associated with viral and bacterial diarrhea have been declining, special attention must be given to the rising incidence of *C*. *difficil*e infections [36]. A key risk factor for *C*. *difficil*e infections is the widespread use of antibiotics, particularly broad-spectrum antibiotics, which disrupt the balance of gut microbiota and allow *C*. *difficil*e to proliferate, causing severe intestinal infections [37]. The misuse of antibiotics has increased the risk of *C*. *difficil*e infections, leading to rising incidence and mortality rates. Additionally, in some developing countries and resource-limited settings, *C*. *difficil*e infections are often underestimated or misdiagnosed, resulting in delayed treatment and high mortality rates. In many cases, the diagnosis and treatment of diarrheal disease in children focus primarily on other pathogens such as rotavirus, S*higella* and *E. coli*, overlooking the possibility of *C*. *difficil*e infection [38]. Moreover, many healthcare facilities, particularly in resource-limited regions, lack effective infection control measures, including proper hand hygiene, environmental sanitation, and isolation practices, which increases the risk of *C*. *difficil*e transmission in hospitals, making children more vulnerable to infection [39]. Furthermore, *C*. *difficil*e has a high recurrence rate, and recurrent infections are more challenging to treat, contributing to increased mortality rates [40].

The study identified rotavirus and *Shigella* as the leading pathogens responsible for diarrhea-related deaths. Reducing diarrhea-related mortality requires a multi-tiered approach with comprehensive interventions [[41], [42], [43], [44]]. First, promoting widespread rotavirus vaccination remains the most effective strategy for preventing rotavirus-induced diarrhea [45], and the development of a *Shigella* vaccine holds promise for further reducing mortality rates [46]. Second, improving access to clean drinking water, enhancing basic sanitation infrastructure, and promoting health education can significantly curb the transmission of pathogens [47]. In addition, nutritional support, such as breastfeeding and zinc supplementation, can boost children's immune systems, reducing both the incidence and severity of diarrhea [48]. In terms of treatment, the use of oral rehydration salts and targeted antibiotics is key to lowering diarrhea-related mortality [49]. In conclusion, global cooperation and financial support are essential for promoting vaccination, improving sanitation, and strengthening community health education [50]. Such preventive measures can significantly reduce the global burden of diarrheal diseases among children under five years of age.

The study has several limitations. Firstly, the analysis relies on data from the GBD study 2021, which may vary in quality and coverage, particularly in developing and low-income countries where data may be incomplete or inaccurate, potentially affecting the reliability of the findings [18,19,51]. Secondly, the GBD study 2021 database is based on model estimations rather than real-world data, which may lead to overestimation or underestimation of the disease burden [18,19,51]. Thirdly, the study focuses primarily on children under five, excluding adolescents, adults, and the elderly. Forth, the predictive models used in this study, such as the BAPC model, while sophisticated, depend on the quality of available data and historical trends [15,16]. Given that the occurrence and transmission of childhood diarrhea are influenced by numerous factors, including climate change and population movement, these models may not fully account for future variations, leading to some uncertainty in the predictions.

## 5 Conclusion

The study reveals that the global incidence, prevalence, mortality, and DALY rates associated with diarrhea in children under five years have significantly declined from 1990 to 2021. However, children in regions with low SDI, particularly in Sub-Saharan Africa and South Asia, continue to bear a high burden of diarrheal disease. Child wasting and underweight remain key risk factors for diarrhea-related mortality. Rotavirus is the leading pathogen responsible for such deaths. Therefore, expanding rotavirus vaccination, improving sanitation, and ensuring access to clean drinking water are critical interventions to effectively reduce the burden of diarrheal disease in children.

## CRediT authorship contribution statement

**Chu Chu:** Writing – review & editing, Writing – original draft, Visualization, Validation, Software, Methodology, Investigation, Formal analysis, Data curation, Conceptualization. **Guobing Yang:** Writing – review & editing, Writing – original draft, Visualization, Validation, Software, Methodology, Investigation, Formal analysis, Data curation, Conceptualization. **Jian Yang:** Writing – review & editing, Writing – original draft, Visualization, Validation, Software, Methodology, Investigation, Formal analysis, Data curation, Conceptualization. **Defeng Liang:** Writing – review & editing, Writing – original draft, Visualization, Validation, Software, Methodology, Investigation, Formal analysis, Data curation, Conceptualization. **Ruitao Liu:** Visualization, Validation, Software, Methodology, Investigation, Data curation. **Guanhua Chen:** Visualization, Validation, Software, Methodology, Investigation, Data curation. **Jichun Wang:** Validation, Supervision, Methodology. **Guisheng Zhou:** Validation, Supervision, Methodology. **Hongli Wang:** Validation, Supervision, Methodology.

## Ethics approval and consent to participate

Not applicable.

## Consent for publication

All authors consent for publication.

## Data availability statement

The datasets analyzed during the current study are available at http://ghdx.healthdata.org/gbd-results-tool.

## Funding sources

The study was supported by the fund of Shanghai Natural Science Foundation (grant number 23ZR1464000), and the Talent Fund of Longhua Hospital affiliated to Shanghai University of Traditional Chinese Medicine (grant number LH001.007), and the Science and Technology Support Project of Taizhou city (SSF20210070).

## Declaration of competing interest

The authors declare that they have no competing interests.
