# Supplementary material for: Trends in epidemiological characteristics and etiologies of diarrheal disease in children under five: an ecological study based on Global Burden of Disease study 2021
Source: Sci One Health. 2024 Nov 1;3:100086. doi: 10.1016/j.soh.2024.100086 (PMC11585739; doi:10.1016/j.soh.2024.100086)
Supplement: Multimedia component 1 [file mmc1.docx]

**Trends in epidemiological characteristics and aetiologies of diarrhoeal disease in children under five: an ecological study based on global burden of disease study 2021**

Chu Chu^a, 1^, Guo-Bing Yang^a, 1^, Jian Yang^a, 1^, De-Feng Liang^d^, Rui-Tao Liu^d^, Guan-Hua Chen^d^, Ji-Chun Wang^c^*, Gui-Sheng Zhou^a^**, Hong-Li Wang^d***^

^a^ National Key Laboratory on Technologies for Chinese Medicine Pharmaceutical Process Control and Intelligent Manufacture, Nanjing University of Chinese Medicine, Nanjing 210023, People's Republic of China.

^b^ Gansu Provincial Center for Disease Control and Prevention, Lanzhou 730000, Gansu, China.

^c^ Chinese Center for Disease Control and Prevention; National Key Laboratory of Intelligent Tracking and Forecasting for Infectious Diseases, Beijing 102206, China.

^d^Guangzhou Women and Children's Medical Center, Guangzhou Medical University, Guangzhou, 510623, China.

^1^ These authors contributed equally to this work.

*Corresponding author. Chinese Center for Disease Control and Prevention. *E-mail address: [wangjc@chinacdc.cn](mailto:wangjc@chinacdc.cn)*

**Nanjing University of Chinese Medicine. *E-mail address*: [zhouguisheng1@njucm.edu.cn](mailto:zhouguisheng1@njucm.edu.cn)

***Correspondence authors: Guangzhou Women and Children's Medical Center. *E-mail address: [2019760935@gzhmu.edu.cn](mailto:2019760935@gzhmu.edu.cn)*

**Items**

Table S1: Incidence cases of diarrhea disease among children under five years in 2021, and change trend of incidence cases were analyzed across GBD regions.

Table S2: Prevalence cases of diarrhea disease among children under five years in 2021, and change trend of prevalence cases were analyzed across GBD regions.

Table S3: The mortality cases of diarrhea disease among children under five years in 2021, and change trend of mortality cases were analyzed across GBD regions.

Figure S1: Trends in the disease burden (rate) of diarrhea in the global and five SDI regions from 1990 to 2021.

Table S5: The change trend of incidence rate of diarrhea disease among children under 5 years old in 204 countries and territories of GBD from 1990 to 2021.

Table S6: The change trend of prevalence rate of diarrhea disease among children under 5 years old in 204 countries and territories of GBD from 1990 to 2021.

Table S7: The change trend of mortality rate of diarrhea disease among children under 5 years old in 204 countries and territories of GBD from 1990 to 2021.

Table S8: The change trend of DALY rate of diarrhea disease among children under 5 years old in 204 countries and territories of GBD from 1990 to 2021.

Figure S2: AAPC in the incidence rate, prevalence rate, mortality rate, and DALY rate of diarrhea disease among children under five years across 204 countries and territories from 1990 to 2021.

Figure S3. The specific rate of diarrheal diseases showed no notable differences across age and gender distributions in children under five years in 2021 year.

Figure S4. The association between the SDI and the rate of diarrhea illness across 204 countries and territories in 2021 year.

Figure S5. The association between the SDI and the rate of diarrhea disease from 1990 to 2021 year.

Table S9: The change trend of mortality number of children under five years old attributed to specific diarrhea pathogens in global from 1990 to 2021.

Table S10: The change trend of DALY number of children under five years old attributed to specific diarrhea pathogens in global from 1990 to 2021.

Table S1: Incidence cases of diarrhea disease among children under five years in 2021, and change trend of incidence cases were analyzed across GBD regions.

| Location | Incidence episodes  (individuals)  (95% UI).  1990 year. | Incidence episodes  (individuals)  (95% UI).  2021 year. | Percentage change of incidence episodes  (95% UI).  1990－2021. | AAPC of incidence number (95% *CI*).  1990－2021. |
| --- | --- | --- | --- | --- |
| Global | 1178110288.50(1000354993.16-1366541107.87) | 392778889.99(324124687.42-463633234.81) | -66.66(-68.35--64.57) | -26073408.91(-26626878.46,-25519939.36) |
| East Asia | 120386256.44(94929796.38-151869249.32) | 11805172.80(8509481.25-15840149.92) | -90.19(-91.62--88.41) | -3579535.11(-3625441.52,-3533628.69) |
| Southeast Asia | 144558581.46(123914857.92-163794574.93) | 62745186.92(51400444.81-76002864.58) | -56.60(-60.09--52.11) | -2511187.29(-2595456.33,-2426918.25) |
| Oceania | 2067612.28(1626372.60-2595318.55) | 508648.34(392608.15-638051.44) | 31.95(19.14-46.54) | 16077.27(14088.24,18066.30) |
| Central Asia | 1884484.37(1645382.50-2140065.46) | 2486583.37(2074341.42-2914336.26) | -86.65(-88.20--85.17) | -386237.74(-389368.92,-383106.56) |
| Central Europe | 13569353.15(11987381.47-15283988.99) | 1812180.72(1489983.12-2160246.03) | -75.40(-76.61--74.26) | -51330.57(-53294.94,-49366.20) |
| Eastern Europe | 20106605.30(16009830.95-25047215.62) | 2635020.23(1952430.14-3399969.92) | -86.89(-88.17--85.28) | -553091.74(-564601.68,-541581.80) |
| High-income Asia Pacific | 19407605.91(13551031.61-26345487.11) | 15502479.95(11131294.71-20392868.89) | -20.68(-25.80--15.24) | -30970.39(-40574.40,-21366.39) |
| Australasia | 6882910.94(4866472.37-9311078.00) | 5459346.45(3902131.33-7237515.24) | -59.96(-63.44--56.40) | -10205.62(-11190.58,-9220.66) |
| Western Europe | 507880.33(365126.45-671706.86) | 203333.83(151079.08-266500.44) | -20.12(-24.03--16.39) | -178180.56(-203009.87,-153351.26) |
| Southern Latin America | 5801008.92(4745879.64-7021946.98) | 1061440.57(787147.27-1382547.86) | -81.70(-83.77--79.39) | -169444.34(-176929.36,-161959.31) |
| High-income North America | 7545114.90(5181664.75-10576161.24) | 642274.98(499749.70-817522.15) | -91.49(-92.49--90.22) | -242943.71(-266932.95,-218954.47) |
| Caribbean | 32395748.39(26553639.15-38804541.14) | 6445992.21(5124296.53-7889307.17) | -69.93(-72.58--67.25) | -111959.61(-113620.37,-110298.84) |
| Andean Latin America | 4933545.69(4205493.37-5670209.82) | 1483432.88(1211873.86-1786443.18) | -88.23(-90.26--86.08) | -458907.31(-469853.76,-447960.86) |
| Central Latin America | 106411109.74(92650053.83-120824075.70) | 32305753.78(26760750.30-37972863.07) | -80.10(-81.72--78.23) | -840068.32(-875761.49,-804375.15) |
| Tropical Latin America | 15756871.01(14494988.60-16953387.33) | 1854613.85(1468522.91-2287470.65) | -79.77(-81.40--78.12) | -746565.76(-762565.85,-730565.67) |
| North Africa and Middle East | 28730852.58(23019747.47-34934912.51) | 5813628.14(4434810.68-7392156.73) | -69.64(-71.85--67.31) | -2334405.88(-2387210.67,-2281601.08) |
| South Asia | 29582047.87(26117742.79-32538832.84) | 16479500.56(13759554.07-19269153.94) | -72.23(-74.99--69.31) | -9081870.94(-9361413.17,-8802328.71) |
| Central Sub-Saharan Africa | 385246785.49(316264984.65-453056743.09) | 106994260.97(88613168.76-127455069.92) | -44.29(-49.92--37.57) | -512170.99(-553855.96,-470486.02) |
| Eastern Sub-Saharan Africa | 103603830.98(91552402.47-113911596.58) | 42046037.02(36246261.37-48452569.18) | -59.42(-62.66--55.66) | -2015629.50(-2108564.18,-1922694.82) |
| Southern Sub-Saharan Africa | 108088161.22(96715647.45-117694047.86) | 69823406.37(58810866.12-81693193.45) | -77.38(-79.91--74.31) | -543622.56(-555062.01,-532183.11) |
| Western Sub-Saharan Africa | 20643921.51(17798334.12-22955093.08) | 4670596.05(3873115.53-5540730.50) | -35.40(-41.90--27.12) | -1172112.10(-1240026.70,-1104197.50) |
| High-middle SDI | 96107436.84(77466419.82-117910530.76) | 20200475.30(15302775.15-25985678.56) | -78.98(-80.87--76.69) | -2476252.14(-2503859.36,-2448644.93) |
| High SDI | 436526221.87(373423256.03-496662334.60) | 132445052.03(111206357.51-155228966.00) | -42.33(-45.87--38.79) | -533637.42(-568720.10,-498554.73) |
| Low-middle SDI | 37611903.46(27244561.84-50267709.48) | 21691429.57(15513334.65-28673257.02) | -69.66(-71.55--67.45) | -9523905.98(-9764399.92,-9283412.04) |
| Low SDI | 253349930.74(222268561.25-281911169.33) | 131195836.51(110403314.81-152409747.81) | -48.22(-51.91--43.74) | -4117419.70(-4282660.98,-3952178.42) |
| Middle SDI | 353833116.84(294476333.30-417960640.36) | 86908065.93(70658803.46-104742712.35) | -75.44(-76.65--74.05) | -8551824.27(-8653272.09,-8450376.45) |

Notes: AAPC=Average Annual Percent Change. CI=Confidence interval. GBD=Global burden of disease. UI=Uncertainty interval. SDI=Socio-demographic index.

Table S2: Prevalence cases of diarrhea disease among children under five years in 2021, and change trend of prevalence cases were analyzed across GBD regions.

| Location | Prevalence episodes  (individuals)  (95% UI).  1990 year. | Prevalence episodes  (individuals)  (95% UI).  2021 year. | Percentage change of prevalence episodes  (95% UI).  1990－2021. | AAPC of prevalence number (95% *CI)*.  1990－2021. |
| --- | --- | --- | --- | --- |
| Global | 19458645.08(17043261.84-22054325.19) | 5825269.66(4975326.02-6775130.90) | -70.06(-71.89--68.07) | -452653.32(-461198.11,-444108.53) |
| East Asia | 1995900.39(1578934.39-2480064.79) | 170059.51(126488.89-223611.18) | -91.48(-92.88--89.97) | -60172.94(-60931.20,-59414.69) |
| Southeast Asia | 2506944.44(2213598.05-2794365.90) | 972180.64(824829.50-1160173.33) | -61.22(-65.08--56.34) | -51765.79(-53534.98,-49996.60) |
| Oceania | 30525.28(27543.21-33729.61) | 37568.65(32589.10-43112.85) | 23.07(11.70-35.95) | 173.92(140.22,207.62) |
| Central Asia | 230192.82(211931.53-251343.51) | 25981.13(22206.30-30146.05) | -88.71(-90.05--87.35) | -6720.15(-6778.28,-6662.03) |
| Central Europe | 35502.93(29093.03-42939.79) | 7795.46(6306.48-9596.03) | -78.04(-79.57--76.70) | -902.02(-934.38,-869.66) |
| Eastern Europe | 327481.38(267795.85-401271.75) | 38853.88(29900.88-49232.16) | -88.14(-89.33--86.90) | -9155.18(-9352.43,-8957.93) |
| High-income Asia Pacific | 106258.43(78571.76-141992.16) | 81519.24(61483.69-105829.29) | -23.28(-29.92--17.18) | -563.99(-719.33,-408.64) |
| Australasia | 7684.66(5793.83-10336.25) | 3121.69(2440.26-4064.63) | -59.38(-63.22--55.52) | -153.03(-168.32,-137.73) |
| Western Europe | 290322.68(210868.68-396536.37) | 237220.24(175146.61-312800.60) | -18.29(-23.07--11.92) | -2566.18(-2934.03,-2198.34) |
| Southern Latin America | 87173.56(73825.68-102068.50) | 15280.32(11868.43-19168.28) | -82.47(-84.62--80.12) | -2293.90(-2393.86,-2193.95) |
| High-income North America | 121328.46(84137.11-170863.15) | 10735.34(8577.52-13425.10) | -91.15(-92.48--89.56) | -3976.13(-4397.79,-3554.47) |
| Caribbean | 74427.92(66602.76-82312.50) | 21176.94(18054.01-24893.96) | -71.55(-73.91--69.10) | -1728.26(-1757.63,-1698.89) |
| Andean Latin America | 285494.59(266164.93-304139.94) | 26404.85(21765.60-31325.86) | -90.75(-92.18--88.99) | -8443.61(-8571.76,-8315.47) |
| Central Latin America | 508370.53(432749.27-595721.31) | 95391.91(79466.99-113630.76) | -81.24(-82.66--79.70) | -13369.42(-13787.00,-12951.85) |
| Tropical Latin America | 480761.82(398953.80-572410.98) | 85297.91(66820.16-106347.46) | -82.26(-83.95--80.71) | -12881.41(-13150.63,-12612.19) |
| North Africa and Middle East | 1834655.64(1652365.29-2027464.46) | 481035.02(417791.30-555113.66) | -73.78(-75.98--71.64) | -42724.09(-43749.02,-41699.16) |
| South Asia | 5969094.79(5059962.96-6980625.88) | 1563594.67(1331633.64-1845411.64) | -73.81(-76.31--70.93) | -144446.41(-148410.15,-140482.66) |
| Central Sub-Saharan Africa | 527631.83(478257.10-575016.60) | 237054.74(204775.22-275899.27) | -55.07(-59.89--49.08) | -10870.23(-11623.68,-10116.77) |
| Eastern Sub-Saharan Africa | 1814230.49(1651112.94-1988476.81) | 621488.58(552903.04-703274.88) | -65.74(-68.80--62.14) | -39729.77(-41408.09,-38051.45) |
| Southern Sub-Saharan Africa | 343519.63(298063.45-387091.98) | 71870.91(61907.27-83339.01) | -79.08(-81.54--76.07) | -9219.07(-9399.14,-9038.99) |
| Western Sub-Saharan Africa | 1881142.81(1695781.38-2050172.57) | 1021638.03(895315.43-1177218.94) | -45.69(-51.35--38.59) | -27162.71(-28087.11,-26238.31) |
| High-middle SDI | 1541524.04(1269327.56-1869385.59) | 296655.22(234063.08-371707.15) | -80.76(-82.70--78.85) | -40425.91(-40932.78,-39919.04) |
| High SDI | 579741.46(437162.80-771923.20) | 327606.45(245544.01-430230.34) | -43.49(-47.40--39.88) | -8461.83(-8998.35,-7925.30) |
| Low-middle SDI | 7189322.07(6355802.86-8108129.62) | 1978346.56(1724115.80-2278399.07) | -72.48(-74.47--70.25) | -162921.62(-166548.45,-159294.80) |
| Low SDI | 4315110.57(3903527.06-4743250.40) | 1916883.12(1679216.82-2195388.80) | -55.58(-59.20--51.07) | -80563.57(-83351.04,-77776.10) |
| Middle SDI | 5821677.26(4972831.80-6742398.68) | 1300693.96(1096498.24-1546401.77) | -77.66(-79.01--76.29) | -144446.55(-145859.18,-143033.92) |

Notes: AAPC=Average Annual Percent Change. CI=Confidence interval. GBD=Global burden of disease. UI=Uncertainty interval. SDI=Socio-demographic index.

Table S3: The mortality cases of diarrhea disease among children under five years in 2021, and change trend of mortality cases were analyzed across GBD regions.

| Location | Mortality cases  (individuals)  (95% UI).  1990 year. | Mortality cases  (individuals)  (95% UI).  2021 year. | Percentage change of mortality cases(95% UI).  1990－2021. | AAPC of death number (95%CI).  1990－2021. |
| --- | --- | --- | --- | --- |
| Global | 1636314.37(1285401.79-1930942.81) | 340429.22(250952.27-464258.39) | -79.20(-84.64--72.44) | -42527.42(-42869.53,-42185.30) |
| East Asia | 72549.52(53671.84-91813.38) | 795.24(602.19-1091.44) | -98.90(-99.20--98.51) | -2339.71(-2387.45,-2291.97) |
| Southeast Asia | 170426.25(106085.80-221428.41) | 13697.52(10285.03-17940.91) | -91.96(-94.30--87.17) | -5059.56(-5147.81,-4971.32) |
| Oceania | 1919.78(1337.23-2819.07) | 1766.42(1064.33-2753.95) | -7.99(-39.53-34.80) | -2.38(-4.46,-0.29) |
| Central Asia | 13675.38(12327.57-15244.45) | 1716.02(1240.59-2306.60) | -87.45(-91.18--83.13) | -393.66(-407.53,-379.78) |
| Central Europe | 772.30(687.47-868.26) | 185.14(150.78-217.63) | -76.03(-80.64--71.01) | -16.05(-17.20,-14.90) |
| Eastern Europe | 1386.07(1314.50-1462.02) | 91.19(80.68-101.08) | -93.42(-94.14--92.71) | -40.59(-43.40,-37.77) |
| High-income Asia Pacific | 172.89(144.20-216.63) | 42.72(37.15-48.60) | -75.29(-80.35--68.87) | -4.06(-4.21,-3.90) |
| Australasia | 13.73(12.08-15.55) | 6.03(4.82-7.49) | -56.06(-66.12--43.83) | -0.21(-0.25,-0.18) |
| Western Europe | 168.35(153.83-183.43) | 133.65(112.79-155.43) | -20.61(-33.49--6.29) | -1.40(-2.36,-0.44) |
| Southern Latin America | 860.23(789.53-936.68) | 88.65(69.56-113.04) | -89.69(-92.01--86.58) | -24.50(-26.17,-22.83) |
| High-income North America | 223.92(211.07-238.27) | 116.11(100.51-132.32) | -48.15(-55.61--40.43) | -3.59(-4.32,-2.86) |
| Caribbean | 12534.14(10280.31-14892.92) | 3974.50(2659.22-5528.02) | -68.29(-79.26--56.27) | -293.69(-333.98,-253.39) |
| Andean Latin America | 7872.95(6580.10-9424.98) | 563.72(368.48-799.34) | -92.84(-95.24--90.06) | -275.55(-295.69,-255.41) |
| Central Latin America | 39758.33(36665.33-43414.18) | 2902.77(2108.52-3872.46) | -92.70(-94.79--90.15) | -1148.63(-1176.17,-1121.10) |
| Tropical Latin America | 31553.24(26930.71-36374.26) | 748.45(584.37-945.63) | -97.63(-98.20--96.91) | -984.45(-998.64,-970.26) |
| North Africa and Middle East | 90384.56(67645.95-115926.96) | 11473.48(8079.78-17689.69) | -87.31(-90.65--83.03) | -2498.70(-2541.23,-2456.16) |
| South Asia | 613477.35(489339.62-736962.40) | 56160.79(34502.71-81504.89) | -90.85(-94.48--86.64) | -18438.28(-18650.59,-18225.98) |
| Central Sub-Saharan Africa | 60616.77(42180.27-76495.68) | 13403.63(8217.90-20759.31) | -77.89(-85.42--63.62) | -1512.31(-1616.10,-1408.52) |
| Eastern Sub-Saharan Africa | 204487.25(128877.06-280208.85) | 66666.73(46246.35-96385.56) | -67.40(-79.74--46.29) | -4485.97(-4638.94,-4333.01) |
| Southern Sub-Saharan Africa | 23642.23(20416.01-27214.81) | 8100.65(5952.53-10796.62) | -65.74(-73.49--56.29) | -527.69(-558.94,-496.43) |
| Western Sub-Saharan Africa | 289819.11(202387.96-360182.11) | 157795.80(106245.46-230122.84) | -45.55(-60.96--22.74) | -4391.29(-4550.39,-4232.20) |
| High-middle SDI | 33085.45(26377.25-39776.37) | 1678.13(1304.74-2104.25) | -94.93(-95.98--93.69) | -1006.05(-1023.38,-988.73) |
| High SDI | 2089.44(1618.33-2821.35) | 424.63(366.97-479.77) | -79.68(-84.97--73.43) | -53.17(-54.19,-52.14) |
| Low-middle SDI | 739330.55(596346.91-863030.59) | 85502.15(64732.28-114556.51) | -88.44(-91.48--84.03) | -21386.95(-21539.48,-21234.42) |
| Low SDI | 572315.76(417109.35-717600.90) | 227078.60(160301.66-315666.55) | -60.32(-72.03--41.53) | -11322.88(-11528.18,-11117.58) |
| Middle SDI | 288482.07(222301.82-342110.39) | 25439.40(19431.65-33425.39) | -91.18(-93.00--89.17) | -8463.73(-8588.67,-8338.78) |

Notes: AAPC=Average Annual Percent Change. CI=Confidence interval. GBD=Global burden of disease. UI=Uncertainty interval. SDI=Socio-demographic index.

Table S4: The DALY cases of diarrhea disease among children under five years in 2021, and change trend of DALY cases were analyzed across GBD regions.

| Location | DALY cases  (individuals)  (95% UI).  1990 year. | DALY cases  (individuals)  (95% UI).  2021 year. | Percentage change of DALY cases  (95% UI).  1990－2021. | AAPC of DALYs number (95% *CI*).  1990－2021. |
| --- | --- | --- | --- | --- |
| Global | 147785001.90(116823041.47-173680475.20) | 30931279.79(23118226.00-41966935.72) | -79.07(-84.40--72.45) | -3832921.67(-3863590.10,-3802253.24) |
| East Asia | 6683248.06(5027004.67-8392858.17) | 90829.90(70728.96-117457.36) | -98.64(-99.00--98.21) | -215154.26(-219569.97,-210738.55) |
| Southeast Asia | 15463619.61(9786917.16-19970273.31) | 1333684.45(1019065.72-1709205.17) | -91.38(-93.76--86.51) | -456393.15(-464311.94,-448474.36) |
| Oceania | 174374.84(122706.36-254996.27) | 161464.26(99610.50-249856.54) | -7.40(-38.21-34.32) | -196.59(-383.82,-9.36) |
| Central Asia | 1246316.15(1123608.22-1387720.58) | 156035.25(112985.19-208346.92) | -87.48(-91.14--83.28) | -35557.20(-37065.23,-34049.16) |
| Central Europe | 73195.52(65723.90-81210.04) | 17471.32(14468.89-20433.52) | -76.13(-80.50--71.37) | -1541.63(-1642.90,-1440.37) |
| Eastern Europe | 161897.56(147088.64-179378.90) | 12682.05(10959.96-14854.94) | -92.17(-92.88--91.38) | -4787.20(-4992.39,-4582.02) |
| High-income Asia Pacific | 27818.56(22284.37-35487.32) | 13321.06(9776.50-18648.26) | -52.11(-60.58--44.36) | -439.76(-466.90,-412.62) |
| Australasia | 2118.24(1730.65-2624.07) | 902.15(735.49-1132.67) | -57.41(-64.00--49.98) | -33.79(-38.42,-29.16) |
| Western Europe | 48915.28(35765.33-68693.28) | 39635.92(29568.09-54280.17) | -18.97(-24.39--13.00) | -318.27(-389.07,-247.47) |
| Southern Latin America | 86978.89(79550.30-94439.14) | 9691.67(7797.02-12151.48) | -88.86(-91.04--86.09) | -2455.88(-2614.85,-2296.90) |
| High-income North America | 34273.78(28625.11-42552.18) | 11655.85(10140.80-13266.47) | -65.99(-72.64--59.19) | -604.88(-672.95,-536.81) |
| Caribbean | 1125812.70(926617.33-1335056.12) | 356778.18(240071.16-495585.84) | -68.31(-79.22--56.35) | -26698.55(-30490.43,-22906.67) |
| Andean Latin America | 733005.98(616411.91-866679.92) | 53206.34(36049.66-74222.43) | -92.74(-95.05--90.08) | -25437.89(-27256.88,-23618.91) |
| Central Latin America | 3601607.76(3324906.36-3932792.70) | 269556.80(198644.06-357487.96) | -92.52(-94.59--90.00) | -103911.04(-106370.72,-101451.36) |
| Tropical Latin America | 2877929.61(2457687.26-3307393.89) | 76685.35(61910.30-94173.94) | -97.34(-97.94--96.59) | -89537.87(-90809.48,-88266.26) |
| North Africa and Middle East | 8271492.40(6236548.87-10554250.87) | 1079163.36(782635.23-1625428.13) | -86.95(-90.23--82.85) | -227242.61(-230977.45,-223507.77) |
| South Asia | 55270939.10(44154165.97-66228227.73) | 5181856.42(3276947.57-7440274.74) | -90.62(-94.19--86.45) | -1643277.18(-1667555.65,-1618998.72) |
| Central Sub-Saharan Africa | 5452145.42(3802716.64-6858691.94) | 1220197.39(757095.14-1880529.86) | -77.62(-85.09--63.57) | -137594.75(-146436.05,-128753.45) |
| Eastern Sub-Saharan Africa | 18376539.36(11668580.13-25086521.93) | 5994967.38(4175441.88-8646755.07) | -67.38(-79.65--46.59) | -403500.89(-417019.29,-389982.48) |
| Southern Sub-Saharan Africa | 2148039.68(1855248.38-2468974.87) | 730458.45(539616.62-969537.29) | -65.99(-73.60--56.79) | -48119.00(-50920.96,-45317.05) |
| Western Sub-Saharan Africa | 25924733.40(18175756.52-32135519.57) | 14121036.24(9586422.15-20520999.58) | -45.53(-60.84--22.98) | -382966.47(-397988.69,-367944.25) |
| High-middle SDI | 3124386.63(2506992.55-3709926.70) | 184218.60(147037.51-222583.97) | -94.10(-95.20--92.77) | -94207.40(-95734.18,-92680.61) |
| High SDI | 254083.05(202408.74-321586.31) | 76189.88(61696.18-95371.42) | -70.01(-77.29--63.08) | -5660.48(-5800.92,-5520.05) |
| Low-middle SDI | 66632099.06(53795719.33-77682087.74) | 7843322.41(5981528.73-10443268.10) | -88.23(-91.22--83.86) | -1922715.84(-1936469.27,-1908962.41) |
| Low SDI | 51316662.06(37628479.89-64249578.64) | 20382047.18(14491345.33-28195004.72) | -60.28(-71.84--41.83) | -1014311.27(-1032785.79,-995836.75) |
| Middle SDI | 26366426.71(20552055.58-31139226.88) | 2417638.08(1881869.20-3098179.72) | -90.83(-92.58--88.88) | -770896.32(-782140.19,-759652.46) |

Notes: AAPC=Average Annual Percent Change. CI=Confidence interval. DALY=disability-adjusted life years. GBD= Global burden of disease. UI=Uncertainty interval. SDI=Socio-demographic index.


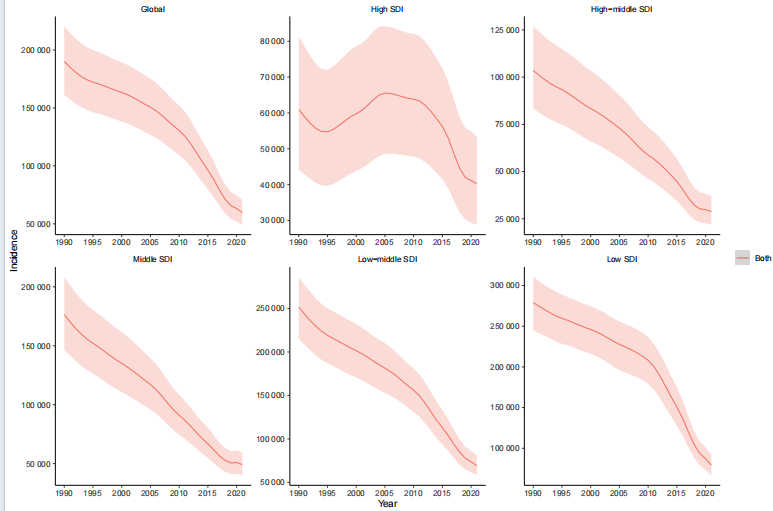


A


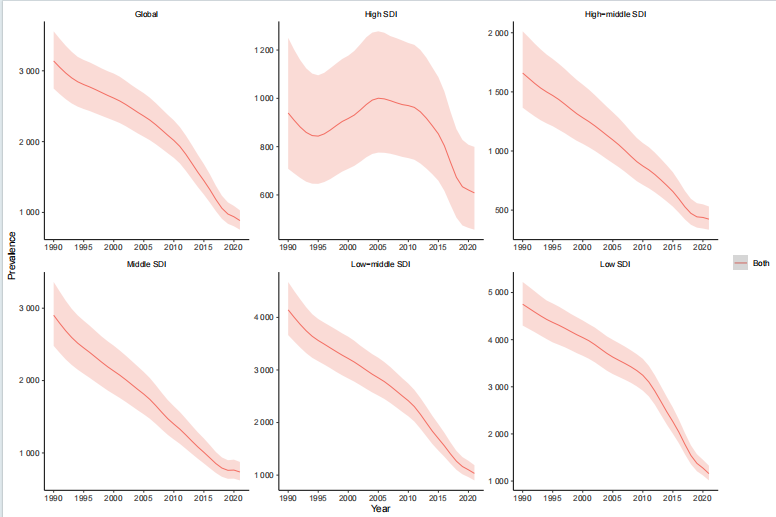


B


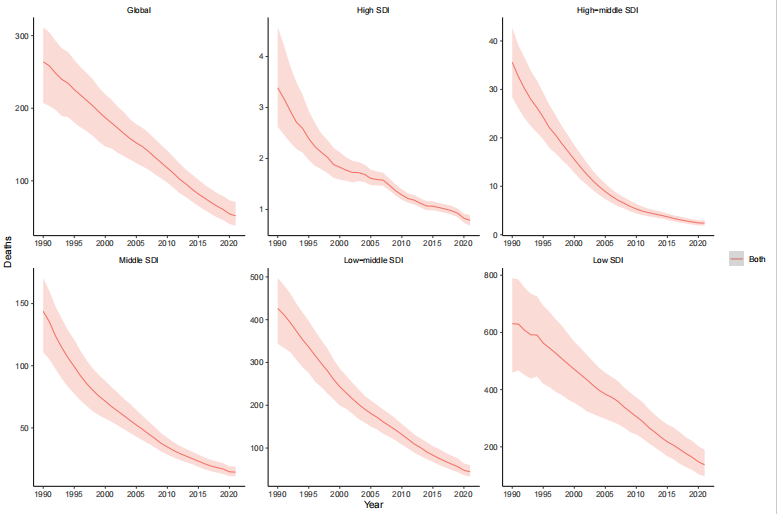


C


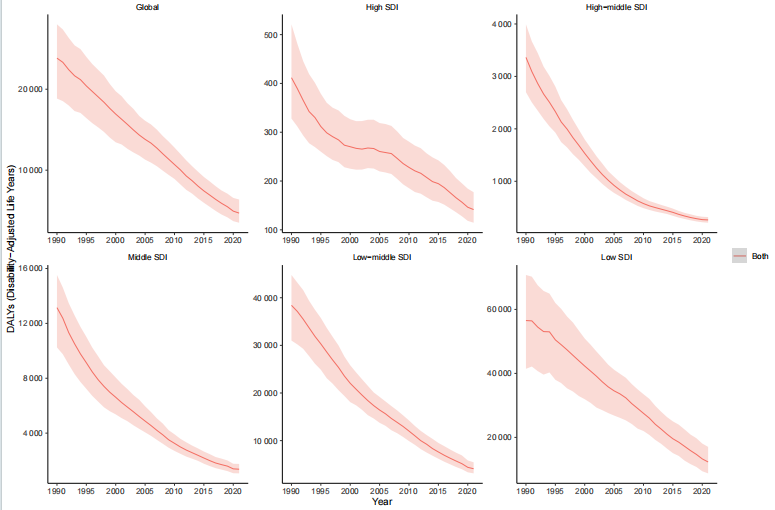


D

Figure S1: Trends in the disease burden (rate) of diarrhea in the global and five SDI regions from 1990 to 2021 (A: Incidence rate , B: Prevalence rate. C: Mortality rate. D: DALYs rate. Abbreviations: DALYs=disability-adjusted life years. SDI=Socio-demographic index.. UI=Uncertainty interval)

Table S5: The change trend of incidence rate of diarrhea disease among children under 5 years old in 204 countries and territories of GBD from 1990 to 2021.

| Location | Incidence rate  (per 100,000 person-years)  (95% UI).  1990 year. | Incidence rate  (per 100,000 person-years)  (95% UI).  2021 year. | AAPC of incidence rate (95% CI).  1990－2021. |
| --- | --- | --- | --- |
| China | 104669.68(82363.81-132130.82) | 11904.60(8513.94-16243.25) | -3051.27(-3094.41,-3008.13) |
| North Korea | 91787.62(74035.81-111843.48) | 86421.92(67884.07-107800.96) | -630.97(-989.95,-271.99) |
| Taiwan | 75619.51(57886.66-97674.74) | 140255.15(98579.60-188169.55) | 2124.30(2026.29,2222.31) |
| Cambodia | 257591.64(219464.27-291990.98) | 70080.36(59624.06-81468.79) | -6315.76(-6522.79,-6108.73) |
| Indonesia | 267283.17(222798.24-307967.69) | 139099.98(112438.21-168753.51) | -4315.63(-4494.04,-4137.22) |
| Laos | 285123.21(264470.24-304357.15) | 99103.79(83784.83-115320.33) | -6401.88(-6580.63,-6223.13) |
| Malaysia | 86401.51(69782.75-104583.67) | 90853.59(64455.98-121176.32) | 248.28(88.42,408.15) |
| Maldives | 254875.65(220938.01-283149.63) | 118298.84(98257.73-140093.93) | -4640.32(-4838.20,-4442.44) |
| Myanmar | 277779.12(255304.06-298097.17) | 122330.42(105141.14-140323.42) | -5060.72(-5184.24,-4937.20) |
| Philippines | 297101.98(255287.47-331641.82) | 97398.34(76718.62-121036.54) | -6571.61(-6795.79,-6347.43) |
| Sri Lanka | 147417.13(122636.48-173228.06) | 68911.48(50868.76-89645.03) | -2795.01(-2974.27,-2615.76) |
| Thailand | 200619.36(167412.52-233418.21) | 65699.57(54227.66-78507.35) | -4723.13(-5024.28,-4421.98) |
| Timor-Leste | 285660.56(256707.81-309502.72) | 110170.23(90793.16-129054.57) | -5913.89(-6090.97,-5736.80) |
| Vietnam | 219126.71(177445.20-262254.09) | 90248.02(67850.20-114170.69) | -4038.52(-4223.14,-3853.90) |
| Fiji | 117220.38(97036.85-141567.47) | 90479.06(74647.56-107796.41) | -967.16(-1041.86,-892.47) |
| Kiribati | 205527.69(173108.79-240031.34) | 67884.87(56210.01-80570.74) | -4474.65(-4505.92,-4443.39) |
| Marshall Islands | 121308.13(97464.38-146238.91) | 44346.77(35581.92-54033.50) | -3054.53(-3289.56,-2819.51) |
| Federated States of Micronesia | 135834.99(109056.42-166750.03) | 43687.75(34212.61-54141.37) | -3548.63(-3750.49,-3346.77) |
| Papua New Guinea | 218963.69(192077.81-247609.67) | 142204.85(118607.08-167193.17) | -2782.13(-2940.34,-2623.93) |
| Samoa | 88815.26(71284.64-108731.49) | 55661.90(43502.63-69531.16) | -1147.64(-1220.32,-1074.96) |
| Solomon Islands | 135123.95(109693.68-164554.04) | 46583.62(38965.27-54675.94) | -2912.48(-2952.68,-2872.27) |
| Tonga | 75590.01(59849.17-92692.22) | 47590.17(34671.57-63403.83) | -928.31(-959.96,-896.66) |
| Vanuatu | 145602.14(117266.01-176578.67) | 64823.49(52552.56-77296.24) | -2521.67(-2684.61,-2358.74) |
| Armenia | 118981.81(102399.31-139370.23) | 18975.74(14913.67-23618.57) | -3098.27(-3246.97,-2949.57) |
| Azerbaijan | 111833.52(96823.70-131189.20) | 28053.51(22183.06-34494.34) | -2810.71(-2881.37,-2740.05) |
| Georgia | 102651.20(88488.84-118187.24) | 24164.65(18976.90-29722.91) | -2590.04(-2767.83,-2412.26) |
| Kazakhstan | 115676.53(101029.34-130447.54) | 15957.22(12047.08-20446.32) | -3321.32(-3362.97,-3279.67) |
| Kyrgyzstan | 128138.58(112183.38-148373.22) | 19609.52(16451.31-22979.90) | -3548.04(-3570.47,-3525.60) |
| Mongolia | 124139.59(100394.91-152273.27) | 10357.03(8754.55-12046.40) | -3956.54(-4190.46,-3722.62) |
| Tajikistan | 247642.15(216673.55-280176.33) | 46219.12(39034.98-52368.87) | -6534.40(-6598.24,-6470.55) |
| Turkmenistan | 207671.05(179509.16-239397.75) | 17591.13(15130.40-20522.04) | -6261.67(-6350.00,-6173.34) |
| Uzbekistan | 137599.91(118417.88-157873.48) | 7695.42(5959.40-9603.30) | -4239.19(-4296.99,-4181.39) |
| Albania | 34879.68(28663.86-41039.28) | 12236.12(9333.44-15545.73) | -693.31(-733.12,-653.49) |
| Bosnia and Herzegovina | 34517.27(27287.92-41974.91) | 17203.08(13312.41-22199.33) | -610.63(-659.10,-562.16) |
| Bulgaria | 21003.25(16858.32-25965.79) | 10204.84(8101.06-12412.66) | -349.67(-355.31,-344.03) |
| Croatia | 13095.33(9984.32-16965.25) | 9622.11(7581.60-11860.33) | -86.20(-95.27,-77.12) |
| Czech Republic | 11485.29(8173.90-15433.67) | 10846.00(8412.26-13609.83) | -15.27(-24.29,-6.25) |
| Hungary | 36854.66(26343.40-48226.05) | 14880.79(11424.35-18762.45) | -703.65(-716.77,-690.53) |
| Macedonia | 82223.50(71751.79-95043.65) | 17521.22(13450.86-22193.22) | -1924.23(-2056.08,-1792.38) |
| Montenegro | 28106.30(21024.31-36511.47) | 13535.86(9653.59-18104.17) | -529.87(-607.11,-452.64) |
| Poland | 20286.73(14993.40-27247.14) | 3231.66(2490.94-4164.67) | -483.55(-531.04,-436.05) |
| Romania | 20372.86(17357.41-23661.03) | 10682.89(8571.23-13053.40) | -287.62(-301.98,-273.27) |
| Serbia | 21910.77(16068.53-28821.43) | 15352.68(11267.95-19823.26) | -229.76(-308.25,-151.26) |
| Slovakia | 9045.42(6751.88-11887.35) | 7183.50(5394.05-9365.87) | -51.35(-59.28,-43.42) |
| Slovenia | 19374.28(14242.87-26013.41) | 20562.83(14778.67-27320.58) | 69.00(45.11,92.90) |
| Belarus | 87748.90(72617.32-103847.09) | 44601.06(33565.58-57979.70) | -1385.55(-1421.21,-1349.89) |
| Estonia | 71592.46(53694.41-90353.27) | 57257.92(42321.16-75678.43) | -488.71(-541.14,-436.29) |
| Latvia | 71847.76(55635.33-91083.13) | 37614.91(28198.47-48003.20) | -1186.18(-1223.44,-1148.92) |
| Lithuania | 74071.46(56259.15-94126.99) | 49996.27(38228.02-63666.51) | -657.29(-703.28,-611.30) |
| Moldova | 100302.99(84950.26-117669.98) | 41318.85(34220.14-49383.85) | -2095.17(-2255.26,-1935.07) |
| Russian Federation | 129841.84(103458.50-160875.24) | 24412.58(18110.95-31509.38) | -3253.36(-3312.26,-3194.45) |
| Ukraine | 90958.56(69926.10-115239.84) | 22876.65(15957.04-30660.96) | -2192.84(-2252.57,-2133.11) |
| Brunei | 14572.69(9998.76-20302.24) | 9146.91(6190.71-12695.57) | -187.95(-200.12,-175.79) |
| Japan | 92436.97(65267.30-125700.70) | 113173.26(81026.65-149857.91) | 740.91(643.87,837.94) |
| South Korea | 21144.17(15007.32-27736.98) | 16203.64(11120.86-22612.98) | -194.77(-225.55,-164.00) |
| Singapore | 10469.12(7873.97-13220.36) | 5558.48(4073.10-7499.14) | -171.52(-181.34,-161.70) |
| Australia | 30131.15(21935.93-39906.60) | 8009.77(5793.08-10775.43) | -714.57(-820.74,-608.40) |
| New Zealand | 45570.22(31700.91-61702.58) | 26522.98(19867.78-34186.97) | -552.13(-672.60,-431.67) |
| Andorra | 97552.25(68526.03-133409.29) | 97947.07(66646.84-136040.60) | -196.04(-341.54,-50.55) |
| Austria | 171412.76(127584.34-220286.07) | 74728.02(56832.85-95910.48) | -3404.94(-3592.61,-3217.27) |
| Belgium | 76674.27(52755.50-105563.48) | 55674.11(40277.07-74099.82) | -813.51(-892.05,-734.97) |
| Cyprus | 87927.79(60849.91-120707.77) | 99723.43(67951.36-137491.45) | 222.57(32.01,413.13) |
| Denmark | 89737.87(62954.79-121006.69) | 104003.06(76252.15-137161.00) | 301.09(91.29,510.88) |
| Finland | 94172.75(66149.43-127281.19) | 62413.69(42647.50-85544.47) | -1065.90(-1235.87,-895.93) |
| France | 95935.04(67806.52-127532.89) | 132411.89(91222.48-179197.04) | 957.14(802.93,1111.34) |
| Germany | 92181.34(63150.97-129009.28) | 55563.51(42841.79-70722.08) | -1569.85(-1882.72,-1256.97) |
| Greece | 99836.34(69246.06-137296.16) | 75775.90(51156.69-103678.94) | -916.55(-1018.47,-814.63) |
| Iceland | 95168.21(65324.87-131229.22) | 96799.62(67231.48-130221.50) | -231.24(-362.72,-99.76) |
| Ireland | 86290.60(57816.00-121533.14) | 91326.60(63868.05-125742.85) | -290.07(-590.92,10.77) |
| Israel | 74670.44(51888.96-102785.42) | 52847.43(37407.24-71897.08) | -931.14(-1041.34,-820.94) |
| Italy | 50877.90(34963.02-70452.45) | 29931.28(21933.51-39361.28) | -553.62(-650.95,-456.28) |
| Luxembourg | 81147.81(57952.24-107672.73) | 61050.01(43421.20-81670.01) | -809.20(-896.97,-721.43) |
| Malta | 87571.71(58092.13-121820.69) | 87007.51(59407.23-117524.58) | -265.60(-380.60,-150.60) |
| Netherlands | 108779.32(74897.84-150947.26) | 166208.95(111931.07-220944.04) | 1751.24(1367.75,2134.72) |
| Norway | 56052.54(38190.70-77612.71) | 47331.95(33702.80-63371.39) | -225.22(-340.69,-109.74) |
| Portugal | 83592.61(62300.37-108222.70) | 52597.11(39060.66-67104.91) | -988.81(-1114.30,-863.32) |
| Spain | 87744.31(61650.96-117414.57) | 82390.24(61286.01-106729.47) | -427.98(-564.59,-291.37) |
| Sweden | 53884.31(37307.85-73561.93) | 50429.33(35928.30-67500.53) | -6.86(-50.09,36.36) |
| Switzerland | 131412.26(97379.68-171424.84) | 131322.85(97824.22-170708.23) | -220.22(-357.12,-83.32) |
| United Kingdom | 71121.31(48603.49-99323.85) | 37977.56(25933.70-52344.02) | -1305.37(-1426.77,-1183.97) |
| Argentina | 132029.17(107364.64-159616.83) | 25060.91(18509.06-33213.37) | -3869.10(-4061.09,-3677.12) |
| Chile | 70064.66(53576.90-87970.09) | 24857.92(18456.38-31458.11) | -1518.05(-1616.26,-1419.83) |
| Uruguay | 93931.76(77062.65-113948.57) | 20642.18(15601.74-27056.83) | -2241.74(-2347.10,-2136.38) |
| Canada | 46962.03(32736.15-64041.77) | 10708.97(7654.01-14186.85) | -1176.94(-1204.11,-1149.76) |
| United States | 33619.05(22953.74-47512.04) | 2358.69(1861.23-2998.47) | -1125.16(-1253.18,-997.13) |
| Antigua and Barbuda | 50148.07(40149.02-62328.07) | 28683.40(21221.75-37978.01) | -667.09(-685.55,-648.63) |
| The Bahamas | 39259.97(30483.85-48744.94) | 17332.45(11830.17-24065.63) | -720.72(-771.94,-669.50) |
| Barbados | 49883.02(38336.50-63557.51) | 35408.70(23993.68-49635.56) | -581.11(-628.81,-533.42) |
| Belize | 88174.65(73741.82-101443.57) | 26506.75(21089.35-32346.35) | -2082.82(-2137.05,-2028.59) |
| Cuba | 43285.78(32242.75-55418.89) | 37292.00(24946.52-52365.05) | -141.10(-198.97,-83.23) |
| Dominica | 37984.32(30129.35-45939.68) | 21758.19(16396.04-27805.75) | -503.60(-520.17,-487.02) |
| Dominican Republic | 192737.41(166584.52-217164.98) | 27820.65(22476.18-33541.43) | -5698.11(-5906.53,-5489.70) |
| Grenada | 47637.45(37489.35-59613.51) | 32030.17(21480.74-45780.70) | -423.39(-493.50,-353.29) |
| Guyana | 61680.83(53381.40-70455.64) | 14596.48(12459.58-17096.42) | -1528.16(-1579.42,-1476.89) |
| Haiti | 157859.17(131418.26-185343.54) | 50523.60(42709.01-59773.21) | -3645.00(-3755.25,-3534.75) |
| Jamaica | 124876.27(106608.73-143336.89) | 22527.02(16890.41-28837.56) | -3307.39(-3377.12,-3237.67) |
| Saint Lucia | 57996.57(47872.21-69460.58) | 33635.15(25178.39-44095.71) | -843.98(-883.49,-804.47) |
| Saint Vincent and the Grenadines | 76524.52(63024.74-90377.53) | 23541.75(18266.23-30116.81) | -1696.16(-1738.32,-1653.99) |
| Suriname | 96076.53(81641.58-110614.59) | 23613.99(20005.32-28107.70) | -2589.35(-2688.91,-2489.80) |
| Trinidad and Tobago | 43508.13(35994.00-52089.80) | 20981.46(15804.96-26950.11) | -774.47(-808.37,-740.58) |
| Bolivia | 251261.51(218699.70-274998.29) | 24364.88(19146.50-29946.32) | -7500.91(-7705.85,-7295.97) |
| Ecuador | 330213.18(302624.91-358620.90) | 21413.44(16831.93-26522.50) | -10050.90(-10216.66,-9885.15) |
| Peru | 299934.48(276847.65-324812.42) | 36598.99(28409.43-45355.99) | -8639.96(-8787.64,-8492.29) |
| Colombia | 130503.80(109467.20-149745.77) | 38917.81(27987.03-49162.86) | -2825.58(-2933.06,-2718.10) |
| Costa Rica | 83629.35(66306.45-100870.78) | 52407.44(37652.69-71287.98) | -981.46(-1021.78,-941.14) |
| El Salvador | 160245.02(134883.50-186641.93) | 27777.32(20525.10-36491.82) | -3983.15(-4124.85,-3841.46) |
| Guatemala | 170556.90(145475.74-200457.16) | 47135.82(39468.75-56048.62) | -4258.58(-4531.32,-3985.84) |
| Honduras | 117545.91(95711.49-142215.04) | 32008.19(25946.89-38920.51) | -2605.72(-2722.22,-2489.21) |
| Mexico | 148525.08(114038.53-188753.53) | 22038.08(16889.16-27820.01) | -4047.63(-4195.51,-3899.75) |
| Nicaragua | 202430.99(169496.45-236150.65) | 28503.66(22209.70-34691.73) | -5749.78(-5829.85,-5669.72) |
| Panama | 164371.84(134676.12-198406.73) | 80904.68(65109.31-97535.26) | -2272.14(-2547.38,-1996.90) |
| Venezuela | 95331.54(79318.49-112215.34) | 47121.58(37832.87-57397.37) | -1673.90(-1752.54,-1595.26) |
| Brazil | 162249.13(128275.90-199683.20) | 34212.25(26078.33-43503.26) | -4236.29(-4333.03,-4139.55) |
| Paraguay | 327565.98(302256.84-351682.11) | 22908.49(17756.81-28562.73) | -10077.81(-10215.92,-9939.70) |
| Algeria | 186587.99(147958.71-230059.80) | 34082.36(26994.14-41938.20) | -4900.06(-4938.06,-4862.06) |
| Bahrain | 54298.28(39497.43-71549.04) | 27304.34(19223.74-36654.12) | -805.44(-859.38,-751.50) |
| Egypt | 249724.69(221072.52-275200.87) | 75069.98(64071.15-87006.71) | -5576.34(-5823.39,-5329.30) |
| Iran | 194395.24(153617.66-242236.11) | 18847.86(14040.39-24736.38) | -5611.45(-5674.37,-5548.52) |
| Iraq | 193390.56(156831.87-232776.62) | 48018.11(40172.26-55856.07) | -4990.66(-5149.65,-4831.67) |
| Jordan | 109706.39(91798.57-130852.88) | 35213.31(27596.42-43700.78) | -2652.07(-2785.57,-2518.56) |
| Kuwait | 125063.04(93784.23-158611.76) | 42790.06(29867.13-58521.88) | -2619.01(-2673.69,-2564.33) |
| Lebanon | 89602.46(71073.45-112211.70) | 52009.18(38240.46-67621.94) | -1376.83(-1481.02,-1272.64) |
| Libya | 137768.16(111200.65-165534.45) | 29461.50(23216.81-36969.16) | -3387.00(-3485.80,-3288.19) |
| Morocco | 254312.95(225688.77-279837.96) | 40517.15(34587.66-46893.99) | -7462.13(-7795.76,-7128.51) |
| Palestine | 79553.87(64611.80-97064.15) | 18586.54(14058.08-23858.60) | -2004.33(-2117.05,-1891.61) |
| Oman | 100311.77(82162.27-118218.56) | 24685.42(18913.54-30814.16) | -2337.01(-2420.33,-2253.69) |
| Qatar | 63927.15(48468.10-81483.62) | 35821.27(27323.42-46617.24) | -970.50(-1015.07,-925.93) |
| Saudi Arabia | 139985.61(113561.93-169003.31) | 33715.28(24686.77-45395.95) | -3434.48(-3536.51,-3332.44) |
| Syrian Arab Republic | 176435.13(145640.14-208623.91) | 36693.63(29775.39-44887.00) | -4759.87(-5001.28,-4518.46) |
| Tunisia | 151695.40(123478.78-184649.84) | 48921.46(37575.63-62113.49) | -3331.49(-3423.06,-3239.92) |
| T眉rkiye | 182310.60(150878.73-215117.00) | 33197.02(25586.87-42709.87) | -5212.47(-5367.10,-5057.84) |
| United Arab Emirates | 84216.53(64590.44-106464.93) | 35460.86(24698.03-48414.40) | -1694.91(-1828.25,-1561.56) |
| Yemen | 292657.14(270906.89-316348.16) | 70369.61(59993.20-81289.59) | -6218.24(-6826.72,-5609.76) |
| Afghanistan | 176413.96(143571.02-211870.66) | 51132.32(43836.61-58146.83) | -4291.07(-4453.26,-4128.89) |
| Bangladesh | 272412.47(229356.46-315294.41) | 16584.74(13874.67-19949.22) | -8267.56(-8340.00,-8195.11) |
| Bhutan | 159374.12(127875.10-192840.13) | 27042.12(21962.75-32669.95) | -4237.14(-4397.98,-4076.30) |
| India | 234255.49(186693.96-281018.14) | 70867.76(57700.46-85324.69) | -5457.83(-5638.15,-5277.52) |
| Nepal | 268633.65(234094.28-297442.98) | 27876.09(23949.75-32162.71) | -7832.13(-8008.21,-7656.04) |
| Pakistan | 283662.17(238700.18-323910.05) | 83522.99(66903.80-101233.84) | -6932.22(-7154.03,-6710.41) |
| Angola | 317675.98(293625.94-347301.56) | 82478.44(69864.06-94819.61) | -8292.47(-8624.62,-7960.33) |
| Central African Republic | 291359.12(262950.41-315191.13) | 100044.66(84295.04-116836.88) | -6439.75(-6610.08,-6269.42) |
| Congo | 296264.10(261566.50-326773.38) | 77068.84(64549.80-90853.22) | -7130.79(-7351.89,-6909.70) |
| Democratic Republic of the Congo | 275752.27(237828.37-308219.89) | 76071.54(62063.47-90470.73) | -6970.09(-7250.07,-6690.12) |
| Equatorial Guinea | 301905.69(276124.34-326421.21) | 42319.31(34832.32-50792.13) | -8559.98(-8853.85,-8266.12) |
| Gabon | 242044.71(195512.91-286137.51) | 52093.94(42760.65-62800.33) | -5983.68(-6105.38,-5861.97) |
| Burundi | 271179.03(231504.92-308414.18) | 83921.34(71118.10-99028.33) | -5775.64(-5991.29,-5560.00) |
| Comoros | 284612.17(240560.65-324038.94) | 69527.20(58573.83-80613.93) | -7073.64(-7218.75,-6928.53) |
| Djibouti | 285068.03(244479.39-321024.63) | 44381.41(36502.61-52808.40) | -8169.61(-8377.58,-7961.63) |
| Eritrea | 310829.96(279070.63-337387.89) | 71946.71(61515.09-83934.83) | -7885.96(-8039.44,-7732.48) |
| Ethiopia | 291827.78(246812.65-335790.09) | 83662.51(69316.25-99381.92) | -7202.87(-7399.12,-7006.61) |
| Kenya | 258988.87(216050.60-299747.33) | 90176.30(76035.79-105589.13) | -5577.64(-5746.77,-5408.51) |
| Madagascar | 345870.39(323085.45-365333.46) | 92316.41(78401.89-108234.53) | -9065.35(-9409.98,-8720.72) |
| Malawi | 299004.39(273427.36-325776.89) | 62747.71(53806.16-71859.26) | -8491.02(-9117.78,-7864.26) |
| Mauritius | 228455.35(198376.88-261538.69) | 94355.68(74614.03-115109.51) | -4683.67(-4925.04,-4442.31) |
| Mozambique | 286159.70(260917.38-307758.88) | 30360.11(25681.79-35371.31) | -8883.81(-9214.78,-8552.84) |
| Rwanda | 295938.35(269860.58-323553.47) | 43905.59(37516.86-50910.76) | -8401.27(-8631.95,-8170.59) |
| Seychelles | 84167.70(68050.37-101151.42) | 45088.38(35268.63-55709.50) | -1273.58(-1319.03,-1228.13) |
| Somalia | 268272.61(229009.93-299397.08) | 58398.22(50147.00-67488.32) | -6863.95(-7065.29,-6662.61) |
| United Republic of Tanzania | 273021.37(235192.65-308094.81) | 34753.30(29425.22-40643.20) | -7194.87(-7516.66,-6873.07) |
| Uganda | 284028.25(249026.58-317717.78) | 53065.98(45977.12-60649.61) | -7304.98(-7634.13,-6975.83) |
| Zambia | 292398.83(268893.64-314667.78) | 56520.16(48436.83-65891.03) | -7858.83(-7981.66,-7736.01) |
| Botswana | 230638.36(194297.21-269282.87) | 75450.67(61891.85-88117.72) | -5457.00(-5732.17,-5181.84) |
| Lesotho | 262658.83(220527.36-304436.81) | 60029.24(50867.65-69345.94) | -6669.40(-6763.63,-6575.18) |
| Namibia | 242812.94(195741.09-286886.25) | 57889.04(47423.68-68405.20) | -6554.62(-6804.61,-6304.62) |
| South Africa | 310731.36(267121.37-344804.02) | 67889.38(54906.03-82726.81) | -8058.75(-8251.94,-7865.57) |
| Eswatini | 270069.76(224137.31-316179.55) | 63368.66(54013.12-72879.03) | -6564.13(-6821.30,-6306.96) |
| Zimbabwe | 193078.72(159383.88-229762.83) | 34042.53(28185.45-39378.33) | -5268.65(-5463.96,-5073.34) |
| Benin | 263858.68(220745.78-303094.31) | 50675.33(42155.12-59283.21) | -6874.99(-6981.69,-6768.29) |
| Burkina Faso | 298415.52(270384.75-323352.03) | 60963.45(50950.56-70862.36) | -8359.10(-8787.68,-7930.53) |
| Cameroon | 282910.48(247501.39-311248.95) | 70149.30(58555.11-82260.14) | -7246.82(-7417.20,-7076.44) |
| Cabo Verde | 259272.72(223643.18-293007.43) | 21979.29(17887.88-27002.97) | -7662.52(-7730.93,-7594.11) |
| Chad | 305257.24(264310.89-340815.00) | 116141.55(99066.22-134503.36) | -6063.60(-6152.84,-5974.37) |
| C么te d'Ivoire | 249501.68(205293.03-291027.92) | 55046.12(45577.20-64255.41) | -6567.73(-6696.41,-6439.05) |
| Gambia | 226108.54(188553.84-261027.41) | 28081.09(23404.18-33763.91) | -7161.17(-7559.61,-6762.72) |
| Ghana | 299286.53(274379.54-322850.25) | 38595.86(32699.66-44857.85) | -8688.26(-8940.03,-8436.48) |
| Guinea | 283287.74(240360.77-321325.74) | 45353.22(38002.67-53278.99) | -8106.29(-8392.84,-7819.74) |
| Guinea-Bissau | 264233.52(220056.22-304802.04) | 42144.53(35336.86-50441.10) | -7682.94(-8060.65,-7305.23) |
| Liberia | 297592.87(261284.98-328546.66) | 72275.02(61145.08-85079.84) | -7784.60(-7984.67,-7584.53) |
| Mali | 315213.74(283440.20-342871.85) | 48987.81(42445.42-55923.38) | -8998.84(-9201.87,-8795.81) |
| Mauritania | 249245.66(207615.41-288649.78) | 71118.96(58858.10-83828.05) | -6097.74(-6294.60,-5900.89) |
| Niger | 322217.45(296881.51-349938.40) | 95601.51(81180.24-110925.74) | -7665.55(-7867.08,-7464.02) |
| Nigeria | 314682.44(275653.28-345521.10) | 112335.35(92473.40-134900.12) | -6500.02(-6959.54,-6040.51) |
| Sao Tome and Principe | 252103.27(208943.08-293133.52) | 13681.60(11181.41-16659.78) | -7891.89(-8193.43,-7590.36) |
| Senegal | 313163.54(288027.30-339207.78) | 64803.04(54267.98-75030.58) | -8035.25(-8188.38,-7882.11) |
| Sierra Leone | 308826.56(271389.89-342672.29) | 56566.50(45137.67-68300.14) | -8732.62(-9045.55,-8419.68) |
| Togo | 315085.90(284428.51-344518.26) | 82771.00(69460.72-97302.34) | -7848.46(-8001.23,-7695.70) |
| American Samoa | 67485.95(53478.81-82764.69) | 58848.91(44630.14-74639.45) | -335.14(-376.23,-294.04) |
| Bermuda | 48099.46(35344.37-63197.74) | 50523.02(33553.57-71370.46) | 19.23(-6.02,44.49) |
| Cook Islands | 70544.26(51666.08-93844.47) | 81462.16(55807.48-109728.09) | 266.97(213.60,320.33) |
| Greenland | 16199.06(11453.30-21726.76) | 5141.83(3625.89-7038.77) | -334.06(-349.61,-318.52) |
| Guam | 70884.59(53128.76-90289.93) | 81137.95(61200.62-101405.27) | 371.89(239.80,503.98) |
| Monaco | 94211.14(64351.37-129375.81) | 90678.88(62228.91-125496.44) | -276.36(-414.54,-138.19) |
| Nauru | 82541.03(65671.96-101613.79) | 61046.46(49429.14-75472.06) | -858.81(-961.31,-756.31) |
| Niue | 84311.10(66892.39-106437.22) | 68791.21(55213.24-85002.96) | -624.28(-680.43,-568.13) |
| Northern Mariana Islands | 75823.39(57478.91-97067.44) | 77032.43(59240.91-97566.69) | 44.36(15.82,72.90) |
| Palau | 112713.73(91044.14-137553.42) | 57076.18(45624.11-69464.11) | -1711.73(-1767.62,-1655.85) |
| Puerto Rico | 56185.24(37601.73-78616.36) | 39845.62(27889.81-53614.43) | -576.55(-600.72,-552.37) |
| Saint Kitts and Nevis | 64103.67(54098.87-73959.31) | 29013.96(23291.68-35232.02) | -1135.41(-1158.20,-1112.63) |
| San Marino | 86042.46(59666.48-117656.26) | 83066.54(58026.53-111549.31) | -284.40(-389.76,-179.04) |
| Tokelau | 81074.77(65192.15-100403.10) | 48840.43(37161.45-63685.40) | -970.24(-1019.38,-921.10) |
| Tuvalu | 115435.32(92892.55-143295.73) | 38875.07(30793.82-47393.36) | -2450.85(-2502.60,-2399.10) |
| United States Virgin Islands | 44295.74(32012.65-58480.33) | 25602.68(17072.10-35744.02) | -666.58(-693.14,-640.02) |
| South Sudan | 305387.35(269558.47-338258.56) | 118209.83(101380.92-134802.76) | -6672.02(-6949.96,-6394.09) |
| Sudan | 293386.92(273990.60-314600.51) | 97004.26(80318.88-114007.15) | -6624.80(-6731.88,-6517.71) |

Notes: AAPC=Average Annual Percent Change. CI=Confidence interval. GBD= Global burden of disease. UI=Uncertainty interval.

Table S6: The change trend of prevalence rate of diarrhea disease among children under 5 years old in 204 countries and territories of GBD from 1990 to 2021.

| Location | Prevalence rate  (per 100,000 person-years)  (95% UI).  1990 year. | Prevalence rate  (per 100,000 person-years)  (95% UI).  2021 year. | AAPC of prevalence rate (95%CI).  1990－2021. |
| --- | --- | --- | --- |
| China | 1734.55(1369.41-2158.81) | 171.14(125.70-230.98) | -51.02(-51.62,-50.43) |
| Democratic People's Republic of Korea | 1539.35(1275.95-1847.43) | 1230.68(1000.69-1498.36) | -17.44(-23.21,-11.67) |
| Taiwan (Province of China) | 1282.74(1012.96-1598.22) | 2075.62(1470.56-2785.73) | 26.51(24.84,28.19) |
| Cambodia | 4606.95(4018.12-5155.11) | 1033.66(902.89-1192.65) | -120.13(-124.19,-116.07) |
| Indonesia | 4697.08(4017.43-5358.42) | 2237.31(1898.21-2671.34) | -82.15(-85.31,-79.00) |
| Lao People's Democratic Republic | 5453.58(5031.97-5803.60) | 1548.34(1366.75-1753.72) | -124.12(-128.50,-119.74) |
| Malaysia | 1245.58(1037.02-1490.92) | 1258.62(916.68-1668.28) | 1.14(-0.16,2.44) |
| Maldives | 4657.35(4090.05-5213.22) | 1710.27(1467.95-1998.78) | -99.49(-102.53,-96.46) |
| Myanmar | 5347.79(4948.23-5746.12) | 1908.99(1701.83-2142.32) | -112.56(-115.20,-109.92) |
| Philippines | 5116.20(4484.76-5734.67) | 1514.24(1228.06-1858.50) | -117.36(-120.81,-113.90) |
| Sri Lanka | 2180.93(1903.64-2473.91) | 951.28(710.03-1223.25) | -43.43(-46.12,-40.74) |
| Thailand | 3247.51(2820.30-3707.69) | 944.96(805.02-1105.24) | -79.04(-83.71,-74.37) |
| Timor-Leste | 5324.84(4752.30-5836.92) | 1672.45(1442.52-1915.80) | -120.19(-121.66,-118.71) |
| Viet Nam | 3596.71(3001.06-4289.86) | 1266.47(991.34-1587.34) | -73.28(-75.82,-70.75) |
| Fiji | 1721.71(1461.69-2034.61) | 1324.17(1146.17-1558.08) | -14.14(-15.25,-13.03) |
| Kiribati | 3247.21(2809.49-3761.98) | 979.04(832.63-1128.84) | -73.36(-73.83,-72.90) |
| Marshall Islands | 1836.06(1534.47-2173.67) | 617.10(512.58-733.98) | -48.09(-51.74,-44.44) |
| Micronesia (Federated States of) | 2085.07(1752.12-2536.03) | 603.05(487.65-738.05) | -56.26(-59.50,-53.01) |
| Papua New Guinea | 3613.86(3258.56-4005.24) | 2159.55(1867.02-2481.24) | -48.50(-51.90,-45.10) |
| Samoa | 1294.54(1060.30-1550.73) | 765.81(610.55-939.21) | -18.23(-19.33,-17.12) |
| Solomon Islands | 2060.69(1763.55-2442.69) | 665.92(575.11-760.35) | -45.52(-46.11,-44.93) |
| Tonga | 1082.10(878.96-1298.38) | 627.08(472.26-813.60) | -15.15(-15.65,-14.66) |
| Vanuatu | 2215.42(1857.10-2651.05) | 935.22(791.84-1091.37) | -39.42(-42.16,-36.67) |
| Armenia | 2175.83(1955.80-2437.78) | 267.59(215.58-325.81) | -59.93(-61.75,-58.10) |
| Azerbaijan | 2098.16(1872.26-2349.13) | 406.85(330.56-491.69) | -56.46(-57.76,-55.17) |
| Georgia | 1634.18(1446.56-1835.93) | 334.12(267.73-404.60) | -42.66(-45.58,-39.73) |
| Kazakhstan | 1965.19(1775.70-2192.32) | 217.81(167.35-274.32) | -57.62(-58.14,-57.11) |
| Kyrgyzstan | 2087.47(1893.10-2323.03) | 282.21(243.73-321.97) | -58.63(-59.15,-58.12) |
| Mongolia | 2007.62(1673.37-2424.13) | 152.53(133.51-175.15) | -64.38(-67.89,-60.87) |
| Tajikistan | 4569.26(4095.53-5047.86) | 692.59(605.49-780.67) | -130.57(-133.25,-127.89) |
| Turkmenistan | 3572.97(3199.73-4015.22) | 251.58(223.06-286.08) | -108.37(-109.98,-106.77) |
| Uzbekistan | 2190.29(1986.49-2418.12) | 105.03(83.53-129.49) | -67.92(-68.91,-66.93) |
| Albania | 561.56(482.79-650.05) | 182.69(142.29-231.60) | -11.34(-12.03,-10.65) |
| Bosnia and Herzegovina | 538.68(448.23-647.96) | 262.75(207.25-330.91) | -10.63(-11.24,-10.03) |
| Bulgaria | 357.66(304.60-417.62) | 158.91(131.98-190.88) | -6.48(-6.59,-6.37) |
| Croatia | 214.75(172.64-268.15) | 147.99(120.65-182.43) | -1.72(-1.91,-1.52) |
| Czechia | 172.76(125.18-232.51) | 165.04(131.45-208.87) | -0.22(-0.34,-0.10) |
| Hungary | 590.49(425.78-771.67) | 224.20(177.51-279.22) | -11.79(-12.00,-11.58) |
| North Macedonia | 1688.79(1522.80-1876.47) | 265.90(210.61-329.01) | -44.12(-46.21,-42.02) |
| Montenegro | 443.77(342.79-577.67) | 204.12(147.27-279.05) | -8.75(-10.06,-7.44) |
| Poland | 341.78(255.87-458.77) | 52.00(41.15-66.31) | -10.15(-10.96,-9.35) |
| Romania | 404.71(357.38-450.48) | 163.40(135.48-195.55) | -7.40(-7.71,-7.09) |
| Serbia | 331.93(253.90-435.04) | 231.47(176.02-298.02) | -3.38(-4.73,-2.02) |
| Slovakia | 135.94(105.64-173.87) | 107.81(84.06-137.21) | -0.76(-0.88,-0.63) |
| Slovenia | 298.43(225.37-395.57) | 320.16(236.87-420.16) | 1.18(0.82,1.55) |
| Belarus | 1377.19(1172.21-1597.03) | 656.09(499.34-840.52) | -23.32(-23.81,-22.83) |
| Estonia | 1074.99(847.64-1341.70) | 852.00(652.51-1106.28) | -7.74(-8.58,-6.90) |
| Latvia | 1081.31(855.98-1338.63) | 554.99(427.23-707.07) | -18.01(-18.57,-17.46) |
| Lithuania | 1114.63(886.53-1379.22) | 737.38(574.73-930.82) | -10.54(-11.14,-9.94) |
| Republic of Moldova | 1928.62(1707.74-2179.74) | 626.11(532.49-737.41) | -38.07(-40.37,-35.76) |
| Russian Federation | 2111.61(1727.62-2581.95) | 362.55(281.05-458.46) | -54.42(-55.48,-53.35) |
| Ukraine | 1483.32(1157.38-1862.77) | 323.41(237.09-427.37) | -38.18(-39.18,-37.17) |
| Brunei Darussalam | 206.45(141.58-284.17) | 126.97(87.67-171.76) | -2.71(-2.90,-2.52) |
| Japan | 1436.95(1058.75-1937.53) | 1691.82(1276.85-2199.36) | 9.44(7.75,11.12) |
| Republic of Korea | 306.93(229.01-395.68) | 236.29(168.49-322.24) | -2.79(-3.26,-2.32) |
| Singapore | 158.65(123.87-198.04) | 84.09(62.46-112.57) | -2.57(-2.71,-2.44) |
| Australia | 456.49(335.74-611.71) | 119.29(89.38-158.78) | -10.89(-12.43,-9.34) |
| New Zealand | 686.90(499.61-941.90) | 424.88(334.23-542.24) | -10.29(-12.85,-7.72) |
| Andorra | 1463.13(1060.48-2045.74) | 1529.87(1058.21-2133.44) | -0.95(-3.07,1.16) |
| Austria | 2807.06(2108.85-3608.39) | 1156.19(907.37-1461.11) | -58.29(-61.51,-55.07) |
| Belgium | 1139.21(803.74-1570.34) | 832.76(606.86-1127.14) | -11.98(-13.12,-10.83) |
| Cyprus | 1302.32(912.18-1814.78) | 1539.90(1068.71-2135.71) | 4.69(1.71,7.67) |
| Denmark | 1318.98(959.95-1802.03) | 1564.84(1164.88-2058.56) | 5.57(2.59,8.56) |
| Finland | 1391.47(992.93-1882.36) | 941.92(662.53-1306.97) | -15.31(-17.92,-12.70) |
| France | 1418.40(1039.55-1871.39) | 2034.43(1433.21-2756.78) | 16.80(14.26,19.35) |
| Germany | 1381.74(952.44-1932.44) | 853.38(667.40-1080.31) | -23.64(-28.84,-18.44) |
| Greece | 1515.27(1073.79-2108.80) | 1155.53(785.46-1597.81) | -13.92(-15.45,-12.39) |
| Iceland | 1418.99(1005.17-1973.18) | 1485.98(1070.09-2047.75) | -2.43(-4.46,-0.40) |
| Ireland | 1297.22(880.09-1807.28) | 1394.24(983.41-1939.72) | -3.63(-8.27,1.01) |
| Israel | 1101.19(781.28-1545.23) | 783.77(561.30-1059.84) | -13.57(-15.21,-11.93) |
| Italy | 759.67(540.30-1060.59) | 476.08(357.26-633.77) | -7.12(-8.80,-5.44) |
| Luxembourg | 1185.65(867.47-1574.34) | 913.47(676.67-1227.23) | -11.13(-12.44,-9.82) |
| Malta | 1316.58(891.19-1844.35) | 1330.48(925.92-1808.08) | -3.29(-5.00,-1.57) |
| Netherlands | 1659.77(1138.31-2337.06) | 2622.19(1800.27-3559.28) | 28.15(21.96,34.33) |
| Norway | 825.22(590.28-1150.06) | 724.70(529.82-987.91) | -2.45(-4.00,-0.89) |
| Portugal | 1307.72(1022.54-1621.01) | 788.20(603.25-1012.88) | -17.09(-19.00,-15.19) |
| Spain | 1289.98(928.49-1765.25) | 1263.92(963.45-1661.35) | -4.72(-6.78,-2.66) |
| Sweden | 800.11(569.79-1129.76) | 762.55(559.98-1057.96) | 0.33(-0.33,1.00) |
| Switzerland | 1960.46(1487.84-2528.20) | 1990.38(1518.34-2593.60) | -2.29(-4.32,-0.27) |
| United Kingdom | 1051.70(737.17-1483.01) | 556.02(398.16-769.10) | -16.16(-16.97,-15.35) |
| Argentina | 1983.93(1688.71-2334.18) | 356.89(276.25-453.91) | -58.59(-61.50,-55.69) |
| Chile | 1040.26(838.75-1267.81) | 369.07(284.92-468.28) | -22.44(-23.83,-21.04) |
| Uruguay | 1479.49(1249.16-1740.80) | 294.20(226.12-375.53) | -35.35(-37.24,-33.46) |
| Canada | 748.36(520.50-1049.71) | 164.04(121.78-220.81) | -19.07(-19.56,-18.57) |
| United States of America | 541.28(372.74-769.95) | 40.95(32.89-51.53) | -18.13(-20.24,-16.03) |
| Antigua and Barbuda | 695.44(566.82-840.71) | 377.60(286.58-489.21) | -9.89(-10.14,-9.64) |
| Bahamas | 537.25(427.29-655.72) | 222.67(159.25-304.75) | -10.35(-11.04,-9.67) |
| Barbados | 683.71(542.36-842.15) | 459.87(324.82-631.45) | -8.86(-9.53,-8.20) |
| Belize | 1277.31(1123.82-1432.61) | 358.08(298.65-431.25) | -30.89(-31.67,-30.11) |
| Cuba | 631.03(499.85-790.45) | 496.16(338.47-695.77) | -3.61(-4.48,-2.74) |
| Dominica | 525.15(432.75-631.78) | 289.96(226.53-368.84) | -7.28(-7.50,-7.05) |
| Dominican Republic | 2932.73(2644.09-3225.29) | 397.51(329.43-475.59) | -87.53(-90.88,-84.19) |
| Grenada | 652.43(537.21-797.20) | 404.63(280.86-560.14) | -7.00(-7.88,-6.12) |
| Guyana | 882.48(786.86-987.88) | 201.56(177.44-229.59) | -22.14(-22.85,-21.42) |
| Haiti | 2440.03(2114.45-2794.32) | 741.31(648.69-850.25) | -54.75(-56.83,-52.68) |
| Jamaica | 1808.55(1618.58-2033.35) | 299.90(232.04-377.46) | -48.59(-49.47,-47.70) |
| Saint Lucia | 815.84(697.01-950.42) | 444.63(346.98-566.74) | -12.72(-13.28,-12.17) |
| Saint Vincent and the Grenadines | 1103.91(944.54-1274.37) | 314.45(248.27-389.23) | -25.23(-25.83,-24.64) |
| Suriname | 1412.51(1244.20-1604.07) | 336.22(291.40-391.33) | -38.37(-39.86,-36.88) |
| Trinidad and Tobago | 607.06(518.60-708.40) | 277.30(215.37-345.11) | -11.25(-11.71,-10.79) |
| Bolivia (Plurinational State of) | 4380.30(3843.26-4864.25) | 353.08(290.08-423.62) | -131.94(-135.86,-128.02) |
| Ecuador | 5890.79(5448.82-6344.19) | 294.97(241.90-358.36) | -182.93(-185.40,-180.45) |
| Peru | 5536.31(5105.62-5932.40) | 523.82(426.71-617.42) | -160.24(-161.69,-158.80) |
| Colombia | 2042.45(1806.88-2309.45) | 551.19(417.62-690.25) | -46.62(-48.15,-45.10) |
| Costa Rica | 1309.01(1095.80-1555.39) | 728.07(537.91-949.52) | -18.87(-19.63,-18.11) |
| El Salvador | 2574.87(2262.51-2908.06) | 377.97(293.26-485.46) | -66.28(-68.53,-64.02) |
| Guatemala | 2775.68(2452.90-3123.54) | 751.47(660.40-854.31) | -57.53(-61.18,-53.87) |
| Honduras | 1826.97(1557.87-2121.94) | 445.21(373.48-527.56) | -41.81(-43.87,-39.75) |
| Mexico | 2287.75(1789.34-2911.00) | 337.41(269.62-422.68) | -62.32(-64.16,-60.48) |
| Nicaragua | 3492.95(3035.83-4028.94) | 398.33(326.57-484.57) | -101.65(-102.79,-100.52) |
| Panama | 2650.05(2259.33-3124.06) | 1255.31(1046.35-1511.50) | -37.33(-42.31,-32.35) |
| Venezuela (Bolivarian Republic of) | 1542.36(1372.17-1750.87) | 674.06(572.97-795.53) | -30.15(-31.39,-28.91) |
| Brazil | 2693.53(2198.01-3257.40) | 502.70(391.93-627.04) | -72.50(-74.10,-70.90) |
| Paraguay | 6053.02(5607.09-6485.39) | 317.28(256.96-389.09) | -187.35(-189.36,-185.35) |
| Algeria | 3069.70(2547.89-3717.33) | 476.43(385.74-577.61) | -83.03(-84.20,-81.85) |
| Bahrain | 744.75(562.05-959.49) | 363.37(263.70-490.00) | -13.41(-14.11,-12.70) |
| Egypt | 4676.46(4346.37-5071.94) | 1191.63(1052.32-1361.64) | -111.07(-115.70,-106.45) |
| Iran (Islamic Republic of) | 3133.03(2534.48-3840.33) | 260.98(201.35-334.97) | -91.65(-92.97,-90.33) |
| Iraq | 3128.49(2644.45-3712.83) | 684.99(593.22-794.38) | -83.98(-86.28,-81.69) |
| Jordan | 1626.48(1376.17-1904.02) | 483.93(393.72-595.83) | -40.46(-42.42,-38.50) |
| Kuwait | 1860.04(1485.74-2282.66) | 586.03(423.86-793.90) | -40.64(-41.47,-39.81) |
| Lebanon | 1309.54(1062.26-1596.11) | 717.39(543.59-921.90) | -21.58(-23.11,-20.05) |
| Libya | 2084.27(1749.97-2449.83) | 402.83(326.58-494.80) | -56.36(-57.79,-54.94) |
| Morocco | 4727.75(4380.00-5074.43) | 610.64(533.18-708.54) | -132.49(-137.33,-127.64) |
| Palestine | 1141.65(958.57-1372.78) | 249.92(197.58-311.64) | -29.48(-31.11,-27.86) |
| Oman | 1496.41(1282.51-1741.89) | 343.13(273.99-420.16) | -35.79(-36.91,-34.67) |
| Qatar | 908.98(720.18-1144.78) | 497.08(387.17-638.98) | -14.13(-14.75,-13.52) |
| Saudi Arabia | 2152.37(1821.78-2568.82) | 447.18(331.60-583.11) | -55.11(-56.53,-53.69) |
| Syrian Arab Republic | 2727.81(2343.19-3162.29) | 505.32(421.13-604.79) | -74.74(-78.40,-71.08) |
| Tunisia | 2296.09(1934.67-2716.09) | 666.86(528.78-843.52) | -52.95(-54.32,-51.58) |
| T眉rkiye | 2861.27(2470.21-3292.87) | 451.81(362.04-564.43) | -79.98(-81.89,-78.07) |
| United Arab Emirates | 1206.72(962.88-1482.14) | 486.19(356.31-659.60) | -24.93(-27.06,-22.80) |
| Yemen | 5589.57(5126.00-5999.72) | 1074.93(942.62-1239.03) | -136.66(-144.82,-128.49) |
| Afghanistan | 2792.65(2361.05-3326.28) | 741.87(663.18-833.76) | -70.62(-73.39,-67.85) |
| Bangladesh | 4158.32(3733.05-4708.60) | 227.30(196.12-265.06) | -126.78(-127.91,-125.65) |
| Bhutan | 2378.10(1976.68-2861.91) | 374.77(308.12-442.27) | -64.01(-66.64,-61.39) |
| India | 3585.99(2959.63-4309.22) | 1035.61(878.14-1233.31) | -84.93(-87.64,-82.22) |
| Nepal | 4557.49(4046.73-5042.98) | 392.05(345.54-441.83) | -136.73(-137.70,-135.76) |
| Pakistan | 4663.82(3985.43-5342.99) | 1229.66(1023.76-1470.11) | -119.33(-124.68,-113.97) |
| Angola | 6029.95(5641.90-6471.53) | 1180.32(1042.93-1334.22) | -160.20(-168.09,-152.30) |
| Central African Republic | 5349.94(4861.66-5793.43) | 1451.87(1282.18-1637.59) | -131.04(-133.85,-128.23) |
| Congo | 5394.89(4854.28-5937.54) | 1108.89(960.90-1287.59) | -141.69(-146.21,-137.16) |
| Democratic Republic of the Congo | 4812.91(4259.45-5314.16) | 1096.34(919.15-1294.86) | -129.24(-133.84,-124.64) |
| Equatorial Guinea | 5623.60(5193.44-6087.20) | 600.97(508.02-709.41) | -163.93(-166.35,-161.50) |
| Gabon | 3797.25(3222.39-4441.23) | 735.39(622.27-868.23) | -96.14(-98.13,-94.15) |
| Burundi | 4412.13(3879.64-4953.79) | 1220.48(1067.35-1401.06) | -116.54(-125.60,-107.49) |
| Comoros | 4961.00(4316.67-5625.47) | 1025.07(894.71-1172.55) | -140.36(-149.05,-131.66) |
| Djibouti | 4952.06(4389.69-5626.00) | 628.28(529.33-735.06) | -146.12(-149.41,-142.82) |
| Eritrea | 5483.32(5026.46-5947.54) | 1026.48(912.38-1155.37) | -142.65(-147.37,-137.92) |
| Ethiopia | 4921.14(4170.65-5663.73) | 1244.04(1061.46-1455.94) | -126.09(-129.55,-122.63) |
| Kenya | 4341.37(3699.19-5004.53) | 1428.74(1245.47-1637.29) | -102.29(-105.72,-98.85) |
| Madagascar | 6402.35(5997.93-6802.59) | 1332.49(1165.76-1486.61) | -178.98(-184.99,-172.97) |
| Malawi | 5561.97(5156.59-5966.12) | 900.33(802.38-1020.94) | -146.07(-150.93,-141.20) |
| Mauritius | 3841.30(3428.74-4260.67) | 1357.43(1120.04-1621.84) | -82.97(-86.87,-79.08) |
| Mozambique | 5333.99(4918.49-5729.28) | 440.90(387.97-504.21) | -158.81(-162.56,-155.05) |
| Rwanda | 5412.06(4979.88-5807.67) | 629.45(553.03-718.90) | -161.68(-166.83,-156.53) |
| Seychelles | 1206.22(1006.86-1430.37) | 629.24(504.65-771.34) | -18.86(-19.57,-18.14) |
| Somalia | 4487.11(3932.62-5070.17) | 855.55(759.24-969.34) | -118.21(-121.52,-114.89) |
| United Republic of Tanzania | 4781.49(4238.18-5295.71) | 489.31(429.22-562.75) | -149.08(-156.52,-141.65) |
| Uganda | 5056.22(4570.33-5586.13) | 763.64(679.76-859.16) | -149.54(-154.04,-145.04) |
| Zambia | 5428.86(5041.94-5825.24) | 841.00(739.42-950.87) | -153.69(-156.93,-150.44) |
| Botswana | 3697.17(3257.80-4152.87) | 1113.76(974.46-1273.60) | -91.32(-95.92,-86.72) |
| Lesotho | 4463.27(3961.11-5037.39) | 926.70(825.14-1043.02) | -116.84(-118.55,-115.14) |
| Namibia | 3803.01(3246.33-4438.99) | 862.41(749.62-980.17) | -103.98(-108.08,-99.88) |
| South Africa | 5247.66(4504.32-5906.39) | 1062.86(893.89-1263.04) | -139.07(-142.33,-135.80) |
| Eswatini | 4564.55(4007.19-5211.90) | 1013.94(902.84-1126.52) | -112.65(-117.39,-107.91) |
| Zimbabwe | 3028.55(2591.93-3489.16) | 488.91(419.03-558.66) | -85.00(-88.31,-81.70) |
| Benin | 4118.61(3559.04-4754.13) | 711.19(608.22-815.05) | -110.37(-111.81,-108.93) |
| Burkina Faso | 5501.30(5052.05-5910.95) | 869.02(757.27-993.64) | -151.20(-161.87,-140.54) |
| Cameroon | 4877.38(4365.44-5378.64) | 1021.10(881.35-1177.32) | -131.77(-135.17,-128.37) |
| Cabo Verde | 4360.47(3919.55-4830.32) | 301.60(252.08-361.32) | -131.05(-132.29,-129.80) |
| Chad | 5226.63(4638.10-5806.26) | 1687.61(1509.19-1884.07) | -108.91(-113.22,-104.59) |
| C么te d'Ivoire | 3973.99(3426.00-4524.80) | 779.58(664.29-898.83) | -108.37(-110.56,-106.18) |
| Gambia | 3361.46(2902.38-3815.67) | 384.46(327.25-450.53) | -102.35(-107.08,-97.61) |
| Ghana | 5488.14(5078.56-5910.20) | 542.57(472.07-617.05) | -171.30(-177.80,-164.79) |
| Guinea | 4542.11(3965.43-5127.46) | 631.26(542.53-722.35) | -126.41(-131.77,-121.06) |
| Guinea-Bissau | 4045.21(3466.82-4648.47) | 588.26(496.89-688.74) | -119.61(-125.39,-113.84) |
| Liberia | 5403.43(4853.02-5929.44) | 1039.43(901.45-1206.15) | -149.65(-154.00,-145.31) |
| Mali | 5530.19(4979.49-6073.07) | 698.38(624.45-779.14) | -155.28(-158.33,-152.22) |
| Mauritania | 3938.01(3384.16-4535.68) | 1020.45(874.18-1173.63) | -100.86(-104.92,-96.80) |
| Niger | 6079.50(5732.93-6492.40) | 1376.06(1219.75-1549.99) | -157.14(-162.38,-151.89) |
| Nigeria | 5454.78(4773.61-6092.71) | 1669.37(1421.59-1976.21) | -112.35(-117.33,-107.36) |
| Sao Tome and Principe | 4037.90(3512.19-4606.83) | 187.31(156.72-222.93) | -136.95(-141.95,-131.94) |
| Senegal | 5595.85(5192.64-5988.60) | 903.23(786.70-1036.04) | -151.76(-154.43,-149.10) |
| Sierra Leone | 5512.12(4924.86-6079.24) | 785.10(644.79-944.86) | -162.87(-169.04,-156.70) |
| Togo | 5603.00(5072.97-6159.92) | 1189.37(1044.45-1365.56) | -149.14(-152.19,-146.08) |
| American Samoa | 950.27(772.47-1166.79) | 808.12(640.86-1011.59) | -4.28(-4.59,-3.96) |
| Bermuda | 655.30(491.66-836.62) | 679.53(466.00-939.64) | -0.03(-0.39,0.34) |
| Cook Islands | 981.62(732.05-1271.69) | 1097.93(788.50-1484.24) | 2.54(1.76,3.32) |
| Greenland | 238.58(175.65-319.11) | 75.79(54.85-102.32) | -4.91(-5.15,-4.67) |
| Guam | 980.75(761.25-1227.53) | 1117.50(889.65-1363.84) | 4.86(2.83,6.88) |
| Monaco | 1414.76(1013.27-1973.62) | 1405.30(991.91-1947.34) | -2.66(-4.72,-0.61) |
| Nauru | 1201.02(996.24-1448.81) | 857.54(719.31-1033.07) | -13.61(-15.17,-12.05) |
| Niue | 1226.07(1002.07-1496.59) | 993.11(831.71-1186.37) | -9.26(-10.04,-8.49) |
| Northern Mariana Islands | 1060.70(835.51-1315.58) | 1064.74(848.90-1300.25) | 0.24(-0.36,0.84) |
| Palau | 1677.20(1396.92-2025.97) | 802.25(661.29-965.02) | -26.63(-27.63,-25.63) |
| Puerto Rico | 772.88(522.35-1065.17) | 542.03(388.81-725.22) | -8.14(-8.50,-7.79) |
| Saint Kitts and Nevis | 913.19(788.88-1038.53) | 397.76(332.29-475.41) | -16.61(-16.89,-16.33) |
| San Marino | 1288.00(917.48-1796.21) | 1273.37(900.01-1742.28) | -3.34(-4.93,-1.76) |
| Tokelau | 1175.73(971.21-1430.93) | 655.87(517.00-818.52) | -18.67(-19.98,-17.36) |
| Tuvalu | 1742.10(1453.10-2096.09) | 528.81(434.57-639.83) | -38.78(-39.49,-38.08) |
| United States Virgin Islands | 597.73(443.06-776.88) | 333.62(224.51-466.99) | -8.65(-8.96,-8.34) |
| South Sudan | 5450.81(4883.41-6038.12) | 1782.65(1600.63-1991.32) | -119.16(-123.14,-115.17) |
| Sudan | 5631.74(5248.04-5977.76) | 1445.55(1241.54-1677.46) | -152.19(-165.46,-138.91) |

Notes: AAPC=Average Annual Percent Change. CI=Confidence interval.GBD= Global burden of disease. UI=Uncertainty interval.

Table S7: The change trend of mortality rate of diarrhea disease among children under 5 years old in 204 countries and territories of GBD from 1990 to 2021.

| Location | Mortality rate  (per 100,000 person-years)  (95% UI).  1990 year. | Mortality rate  (per 100,000 person-years)  (95% UI).  2021 year. | AAPC of death rate (95% *CI*).  1990－2021. |
| --- | --- | --- | --- |
| China | 64.73(47.88-81.97) | 1.00(0.74-1.38) | -2.07(-2.12,-2.03) |
| Democratic People's Republic of Korea | 5.03(3.06-7.96) | 1.15(0.55-1.82) | -0.12(-0.12,-0.12) |
| Taiwan (Province of China) | 3.69(3.27-4.10) | 0.37(0.29-0.46) | -0.11(-0.12,-0.11) |
| Cambodia | 298.26(205.77-410.96) | 16.22(10.96-24.16) | -9.36(-9.56,-9.17) |
| Indonesia | 485.76(312.40-615.76) | 37.71(27.87-49.99) | -14.28(-14.59,-13.97) |
| Lao People's Democratic Republic | 750.82(469.84-1056.71) | 46.32(28.37-73.47) | -22.75(-23.24,-22.27) |
| Malaysia | 15.84(10.28-21.03) | 3.09(2.06-4.58) | -0.39(-0.40,-0.37) |
| Maldives | 319.07(207.32-405.43) | 10.89(7.27-15.52) | -9.98(-10.17,-9.80) |
| Myanmar | 523.32(240.71-860.83) | 33.66(21.98-49.37) | -16.23(-16.39,-16.07) |
| Philippines | 175.58(123.52-229.56) | 22.15(16.35-30.45) | -4.95(-5.06,-4.85) |
| Sri Lanka | 49.18(35.99-65.84) | 2.93(1.93-4.18) | -1.50(-1.55,-1.45) |
| Thailand | 53.33(19.12-92.73) | 7.36(5.26-9.78) | -1.45(-1.48,-1.42) |
| Timor-Leste | 653.59(354.12-901.09) | 42.72(24.15-67.56) | -19.89(-20.12,-19.65) |
| Viet Nam | 34.49(16.03-56.07) | 1.10(0.52-1.76) | -1.08(-1.10,-1.06) |
| Fiji | 33.73(22.99-47.95) | 17.54(9.86-27.70) | -0.53(-0.57,-0.49) |
| Kiribati | 277.66(200.92-384.43) | 59.28(36.76-86.27) | -7.05(-7.23,-6.87) |
| Marshall Islands | 40.29(22.71-63.38) | 14.63(8.51-22.63) | -0.56(-1.21,0.08) |
| Micronesia (Federated States of) | 46.69(30.57-68.37) | 9.04(5.35-14.00) | -0.65(-1.60,0.31) |
| Papua New Guinea | 253.96(170.92-377.39) | 107.35(63.94-169.66) | -4.81(-5.04,-4.58) |
| Samoa | 8.83(3.26-14.39) | 3.21(1.38-5.27) | -0.18(-0.18,-0.17) |
| Solomon Islands | 85.29(54.31-128.78) | 17.51(11.25-25.47) | -2.17(-2.28,-2.05) |
| Tonga | 10.96(6.74-16.33) | 3.57(1.91-5.92) | -0.22(-0.23,-0.21) |
| Vanuatu | 68.80(39.05-115.62) | 19.28(9.91-36.07) | -1.53(-1.67,-1.38) |
| Armenia | 98.05(82.91-114.74) | 2.09(1.57-2.78) | -3.19(-3.41,-2.96) |
| Azerbaijan | 165.22(126.19-212.47) | 23.52(14.39-37.71) | -4.55(-4.62,-4.49) |
| Georgia | 41.97(34.91-50.01) | 0.86(0.60-1.21) | -1.20(-1.33,-1.07) |
| Kazakhstan | 87.09(76.24-97.61) | 1.51(1.06-2.07) | -2.84(-3.01,-2.68) |
| Kyrgyzstan | 118.32(101.11-135.55) | 7.13(5.57-9.03) | -3.71(-3.91,-3.51) |
| Mongolia | 60.76(35.98-97.62) | 5.83(2.26-12.56) | -1.71(-1.75,-1.66) |
| Tajikistan | 358.53(290.62-440.59) | 97.63(65.07-136.88) | -8.81(-9.09,-8.53) |
| Turkmenistan | 268.55(231.91-313.19) | 8.00(5.80-10.80) | -8.73(-9.04,-8.42) |
| Uzbekistan | 119.80(105.22-135.98) | 2.13(1.56-2.88) | -3.97(-4.18,-3.75) |
| Albania | 24.70(18.02-33.59) | 4.20(2.22-6.25) | -0.67(-0.73,-0.61) |
| Bosnia and Herzegovina | 4.54(3.16-6.16) | 2.31(1.55-3.38) | -0.08(-0.09,-0.06) |
| Bulgaria | 4.22(3.48-5.13) | 3.99(2.96-5.12) | -0.04(-0.06,-0.02) |
| Croatia | 2.01(1.65-2.46) | 2.61(1.86-3.42) | 0.01(0.01,0.02) |
| Czechia | 5.17(4.19-6.23) | 5.39(4.22-6.72) | 0.01(-0.01,0.04) |
| Hungary | 4.80(3.82-5.95) | 6.59(4.88-8.43) | 0.07(0.05,0.09) |
| North Macedonia | 74.09(57.77-97.66) | 6.52(4.62-9.29) | -2.20(-2.30,-2.11) |
| Montenegro | 0.90(0.60-1.40) | 0.22(0.11-0.41) | -0.02(-0.02,-0.02) |
| Poland | 2.05(1.80-2.31) | 2.09(1.62-2.61) | 0.02(0.01,0.03) |
| Romania | 18.06(15.21-21.12) | 4.12(3.09-5.23) | -0.35(-0.39,-0.30) |
| Serbia | 5.32(2.17-8.26) | 1.42(0.91-2.21) | -0.12(-0.13,-0.10) |
| Slovakia | 2.17(1.54-2.86) | 1.89(1.13-3.20) | -0.01(-0.01,-0.00) |
| Slovenia | 0.83(0.66-1.02) | 0.74(0.53-1.00) | -0.00(-0.00,0.00) |
| Belarus | 4.36(3.46-5.66) | 0.41(0.28-0.59) | -0.12(-0.14,-0.10) |
| Estonia | 3.91(3.38-4.50) | 0.25(0.19-0.33) | -0.11(-0.12,-0.10) |
| Latvia | 3.26(2.71-3.99) | 0.24(0.18-0.34) | -0.10(-0.11,-0.09) |
| Lithuania | 4.85(4.07-5.74) | 0.53(0.40-0.71) | -0.14(-0.16,-0.12) |
| Republic of Moldova | 21.55(17.67-26.02) | 2.21(1.51-3.23) | -0.57(-0.62,-0.52) |
| Russian Federation | 9.07(8.64-9.54) | 0.97(0.84-1.08) | -0.27(-0.29,-0.25) |
| Ukraine | 4.74(4.07-5.53) | 0.71(0.56-0.94) | -0.11(-0.13,-0.10) |
| Brunei Darussalam | 1.35(0.92-1.87) | 1.21(0.80-1.71) | -0.00(-0.01,-0.00) |
| Japan | 0.99(0.92-1.05) | 0.65(0.55-0.75) | -0.01(-0.01,-0.01) |
| Republic of Korea | 3.09(2.21-4.38) | 0.74(0.48-1.10) | -0.07(-0.07,-0.07) |
| Singapore | 2.13(1.84-2.46) | 0.41(0.32-0.53) | -0.05(-0.06,-0.05) |
| Australia | 0.81(0.70-0.93) | 0.23(0.17-0.29) | -0.02(-0.02,-0.02) |
| New Zealand | 1.25(1.08-1.43) | 0.84(0.68-1.02) | -0.01(-0.01,-0.00) |
| Andorra | 0.16(0.09-0.25) | 0.04(0.02-0.06) | -0.00(-0.00,-0.00) |
| Austria | 0.45(0.39-0.52) | 0.21(0.17-0.26) | -0.01(-0.01,-0.01) |
| Belgium | 1.44(1.22-1.70) | 1.64(1.26-2.11) | -0.00(-0.01,0.01) |
| Cyprus | 4.88(2.28-7.16) | 0.93(0.59-1.43) | -0.13(-0.13,-0.13) |
| Denmark | 1.45(1.27-1.67) | 1.21(0.93-1.49) | -0.01(-0.02,-0.00) |
| Finland | 0.32(0.27-0.37) | 0.35(0.28-0.44) | -0.00(-0.00,0.00) |
| France | 1.42(1.24-1.62) | 0.82(0.65-1.00) | -0.03(-0.03,-0.02) |
| Germany | 0.78(0.66-0.92) | 0.65(0.52-0.81) | -0.00(-0.01,0.00) |
| Greece | 0.08(0.07-0.09) | 0.14(0.11-0.18) | 0.00(0.00,0.00) |
| Iceland | 0.31(0.26-0.38) | 0.34(0.25-0.44) | -0.00(-0.00,0.00) |
| Ireland | 0.25(0.22-0.29) | 0.23(0.18-0.30) | -0.00(-0.00,0.00) |
| Israel | 1.77(1.51-2.07) | 1.16(0.91-1.45) | -0.02(-0.02,-0.01) |
| Italy | 0.29(0.27-0.31) | 0.75(0.59-0.94) | 0.02(0.01,0.02) |
| Luxembourg | 0.96(0.82-1.13) | 0.90(0.70-1.16) | -0.00(-0.01,0.00) |
| Malta | 0.18(0.14-0.21) | 0.30(0.22-0.40) | 0.00(0.00,0.01) |
| Netherlands | 0.47(0.41-0.53) | 0.68(0.55-0.81) | 0.01(0.00,0.01) |
| Norway | 0.14(0.12-0.16) | 0.20(0.16-0.24) | 0.00(0.00,0.00) |
| Portugal | 2.09(1.84-2.43) | 0.44(0.34-0.54) | -0.05(-0.06,-0.04) |
| Spain | 0.65(0.57-0.73) | 0.56(0.45-0.66) | -0.00(-0.01,0.00) |
| Sweden | 0.06(0.06-0.07) | 0.40(0.32-0.48) | 0.01(0.01,0.01) |
| Switzerland | 0.84(0.72-0.95) | 0.60(0.46-0.74) | -0.01(-0.01,-0.01) |
| United Kingdom | 0.17(0.16-0.18) | 0.28(0.23-0.32) | 0.02(0.00,0.03) |
| Argentina | 18.79(16.88-20.60) | 2.14(1.64-2.77) | -0.54(-0.58,-0.50) |
| Chile | 11.24(10.08-12.36) | 1.65(1.33-2.06) | -0.30(-0.31,-0.29) |
| Uruguay | 19.43(17.25-22.00) | 3.40(2.56-4.40) | -0.52(-0.59,-0.45) |
| Canada | 0.37(0.31-0.43) | 0.93(0.72-1.16) | 0.02(0.02,0.02) |
| United States of America | 1.10(1.03-1.17) | 0.53(0.46-0.61) | -0.02(-0.02,-0.02) |
| Antigua and Barbuda | 11.10(8.88-13.70) | 3.33(2.61-4.10) | -0.22(-0.24,-0.21) |
| Bahamas | 14.45(11.38-18.17) | 2.45(1.72-3.40) | -0.45(-0.48,-0.42) |
| Barbados | 9.18(7.40-11.00) | 1.89(1.25-2.74) | -0.23(-0.25,-0.21) |
| Belize | 89.98(77.71-103.55) | 9.22(7.02-11.56) | -2.63(-2.75,-2.50) |
| Cuba | 15.43(13.92-17.04) | 2.06(1.57-2.59) | -0.42(-0.45,-0.39) |
| Dominica | 16.50(11.68-22.53) | 12.14(7.10-18.89) | -0.15(-0.16,-0.13) |
| Dominican Republic | 215.48(169.65-275.32) | 24.78(12.84-36.72) | -6.17(-6.31,-6.03) |
| Grenada | 15.16(12.07-19.35) | 2.39(1.77-3.11) | -0.40(-0.43,-0.37) |
| Guyana | 156.17(125.85-188.62) | 19.11(13.47-26.40) | -4.28(-4.71,-3.84) |
| Haiti | 880.89(690.79-1065.03) | 224.53(148.46-316.16) | -22.49(-25.40,-19.58) |
| Jamaica | 54.59(46.45-64.23) | 5.33(3.67-7.45) | -1.57(-1.62,-1.52) |
| Saint Lucia | 29.31(23.67-35.98) | 4.40(3.06-6.42) | -0.77(-0.80,-0.75) |
| Saint Vincent and the Grenadines | 57.37(44.44-71.77) | 6.32(4.59-8.74) | -1.65(-1.69,-1.60) |
| Suriname | 131.69(96.40-172.98) | 28.37(17.60-41.07) | -3.39(-3.45,-3.33) |
| Trinidad and Tobago | 22.34(17.66-27.33) | 4.29(3.03-6.11) | -0.58(-0.61,-0.54) |
| Bolivia (Plurinational State of) | 223.15(146.49-322.12) | 23.90(14.24-35.10) | -7.02(-7.31,-6.73) |
| Ecuador | 132.59(121.47-145.45) | 3.61(2.53-5.18) | -4.91(-5.35,-4.47) |
| Peru | 131.07(103.36-165.35) | 6.62(3.75-10.22) | -4.80(-5.20,-4.40) |
| Colombia | 81.67(69.94-92.74) | 6.22(4.14-9.19) | -2.44(-2.51,-2.36) |
| Costa Rica | 21.68(18.87-24.45) | 2.50(1.93-3.12) | -0.62(-0.66,-0.59) |
| El Salvador | 229.06(185.98-279.86) | 10.07(6.24-14.87) | -7.25(-7.41,-7.09) |
| Guatemala | 364.79(327.98-411.33) | 60.51(45.38-80.15) | -9.81(-10.13,-9.49) |
| Honduras | 261.17(212.90-314.33) | 27.07(16.40-39.03) | -7.70(-7.84,-7.55) |
| Mexico | 180.72(162.60-202.95) | 7.63(5.46-10.36) | -5.71(-5.87,-5.55) |
| Nicaragua | 314.30(254.06-405.89) | 15.08(9.10-22.12) | -9.64(-9.83,-9.46) |
| Panama | 57.51(47.23-68.37) | 15.62(11.50-20.87) | -1.40(-1.50,-1.29) |
| Venezuela (Bolivarian Republic of) | 124.63(116.17-134.22) | 21.55(10.00-30.38) | -2.70(-3.12,-2.28) |
| Brazil | 188.50(160.27-217.19) | 4.19(3.23-5.30) | -6.01(-6.09,-5.92) |
| Paraguay | 84.72(63.41-111.13) | 8.44(4.49-14.39) | -2.65(-2.75,-2.55) |
| Algeria | 28.23(10.03-47.53) | 3.88(1.64-5.83) | -0.77(-0.78,-0.75) |
| Bahrain | 13.79(10.04-18.50) | 2.01(1.27-2.78) | -0.39(-0.40,-0.38) |
| Egypt | 323.54(234.24-433.08) | 21.07(14.13-30.49) | -9.74(-9.84,-9.65) |
| Iran (Islamic Republic of) | 39.36(24.48-67.85) | 1.16(0.83-1.53) | -1.23(-1.26,-1.21) |
| Iraq | 71.28(48.37-101.71) | 11.40(7.60-16.41) | -1.96(-2.06,-1.86) |
| Jordan | 15.95(10.81-22.78) | 3.36(2.29-4.89) | -0.41(-0.42,-0.41) |
| Kuwait | 5.13(4.30-6.06) | 0.89(0.68-1.15) | -0.11(-0.13,-0.09) |
| Lebanon | 26.24(14.30-38.89) | 6.49(3.99-10.07) | -0.66(-0.71,-0.62) |
| Libya | 51.08(28.45-80.51) | 4.88(1.33-8.55) | -1.47(-1.52,-1.41) |
| Morocco | 309.29(235.95-397.26) | 19.47(11.81-30.71) | -9.14(-9.31,-8.97) |
| Palestine | 38.64(24.91-60.48) | 2.57(1.67-3.94) | -1.18(-1.20,-1.16) |
| Oman | 23.24(12.37-40.89) | 3.99(2.59-5.88) | -0.58(-0.60,-0.56) |
| Qatar | 5.88(3.75-8.76) | 1.27(0.81-1.84) | -0.15(-0.15,-0.14) |
| Saudi Arabia | 49.85(31.15-79.29) | 2.79(1.68-4.19) | -1.51(-1.54,-1.48) |
| Syrian Arab Republic | 33.89(22.05-50.68) | 2.41(1.18-3.76) | -1.00(-1.01,-0.98) |
| Tunisia | 27.82(11.76-44.40) | 2.78(1.03-4.52) | -0.80(-0.81,-0.79) |
| T眉rkiye | 75.05(51.30-118.39) | 4.30(3.06-6.27) | -2.26(-2.31,-2.20) |
| United Arab Emirates | 10.10(5.99-14.74) | 2.53(1.80-3.60) | -0.25(-0.26,-0.23) |
| Yemen | 510.14(335.55-738.21) | 27.69(9.64-56.79) | -15.31(-15.65,-14.97) |
| Afghanistan | 427.27(273.74-618.69) | 72.17(46.53-109.11) | -12.52(-13.13,-11.91) |
| Bangladesh | 255.66(189.63-379.52) | 15.88(9.90-26.06) | -7.85(-8.01,-7.70) |
| Bhutan | 608.84(269.39-1018.34) | 39.06(18.82-69.01) | -19.05(-19.71,-18.39) |
| India | 406.12(309.77-503.76) | 34.02(19.75-50.97) | -12.33(-12.57,-12.10) |
| Nepal | 425.41(315.79-548.73) | 20.04(11.00-30.24) | -13.05(-13.31,-12.79) |
| Pakistan | 424.64(336.51-534.05) | 51.67(30.65-84.74) | -12.05(-12.19,-11.90) |
| Angola | 1081.09(743.45-1468.68) | 60.13(37.75-86.14) | -33.10(-33.81,-32.38) |
| Central African Republic | 769.95(461.27-1055.70) | 314.52(182.00-510.73) | -14.29(-14.65,-13.93) |
| Congo | 407.57(259.27-620.28) | 54.43(24.23-114.23) | -11.07(-11.58,-10.57) |
| Democratic Republic of the Congo | 451.35(306.28-603.60) | 51.19(24.58-96.91) | -12.59(-13.21,-11.97) |
| Equatorial Guinea | 867.53(488.95-1247.25) | 20.85(10.51-37.21) | -28.25(-29.31,-27.20) |
| Gabon | 245.55(123.89-455.10) | 24.62(7.88-56.95) | -7.08(-7.25,-6.92) |
| Burundi | 402.06(249.66-592.19) | 89.11(36.71-189.94) | -10.50(-10.85,-10.14) |
| Comoros | 276.52(162.84-441.66) | 50.60(24.61-86.21) | -7.35(-8.67,-6.02) |
| Djibouti | 452.24(298.51-653.02) | 45.23(21.52-84.53) | -12.27(-13.58,-10.96) |
| Eritrea | 693.19(479.94-913.55) | 92.86(54.05-149.70) | -20.59(-21.45,-19.72) |
| Ethiopia | 570.57(285.89-888.83) | 85.28(57.89-129.07) | -15.93(-16.37,-15.50) |
| Kenya | 395.51(239.40-516.14) | 81.05(59.97-106.02) | -10.38(-10.67,-10.10) |
| Madagascar | 714.85(565.53-873.51) | 212.24(119.91-347.59) | -16.27(-16.58,-15.96) |
| Malawi | 882.10(553.70-1162.55) | 86.14(50.36-136.81) | -26.65(-27.32,-25.97) |
| Mauritius | 26.08(23.29-29.60) | 6.60(5.20-7.88) | -0.57(-0.64,-0.50) |
| Mozambique | 621.67(369.22-958.18) | 75.34(43.21-130.64) | -17.36(-18.07,-16.65) |
| Rwanda | 462.58(276.20-659.47) | 68.13(42.49-103.11) | -10.98(-17.58,-4.38) |
| Seychelles | 15.50(11.17-21.21) | 6.19(3.88-9.10) | -0.32(-0.41,-0.24) |
| Somalia | 804.18(454.53-1155.13) | 237.20(135.62-353.16) | -17.96(-18.97,-16.96) |
| United Republic of Tanzania | 451.64(317.57-621.52) | 70.08(39.60-113.87) | -12.67(-13.12,-12.22) |
| Uganda | 453.40(196.95-720.80) | 53.28(25.68-99.92) | -13.35(-13.62,-13.08) |
| Zambia | 652.71(412.73-910.15) | 77.20(47.99-116.69) | -19.10(-20.48,-17.73) |
| Botswana | 274.80(206.97-356.67) | 125.55(76.98-187.76) | -3.97(-5.51,-2.42) |
| Lesotho | 546.27(444.46-675.36) | 299.09(192.27-421.65) | -9.33(-10.19,-8.48) |
| Namibia | 351.76(258.74-458.14) | 120.69(70.00-178.01) | -7.99(-8.35,-7.63) |
| South Africa | 367.47(311.11-427.04) | 94.53(71.99-122.41) | -9.17(-9.68,-8.65) |
| Eswatini | 490.84(373.16-615.15) | 140.68(86.96-218.20) | -12.16(-12.83,-11.49) |
| Zimbabwe | 129.00(90.52-163.12) | 89.25(44.61-130.70) | -2.39(-3.63,-1.15) |
| Benin | 491.23(318.81-692.90) | 81.59(37.31-167.63) | -13.57(-13.89,-13.26) |
| Burkina Faso | 777.28(516.91-1056.14) | 180.34(119.25-266.86) | -18.59(-19.16,-18.02) |
| Cameroon | 367.76(232.80-558.15) | 87.85(41.94-171.49) | -9.03(-9.56,-8.50) |
| Cabo Verde | 269.01(187.62-365.59) | 20.48(12.94-30.20) | -7.03(-9.41,-4.65) |
| Chad | 1211.62(781.92-1660.14) | 560.45(373.77-952.59) | -22.01(-23.06,-20.95) |
| C么te d'Ivoire | 365.09(256.04-502.96) | 77.36(43.18-131.59) | -9.86(-10.31,-9.42) |
| Gambia | 413.01(271.53-562.90) | 62.31(41.49-87.81) | -11.41(-11.99,-10.84) |
| Ghana | 293.24(183.93-415.19) | 32.20(19.09-48.67) | -8.22(-8.54,-7.89) |
| Guinea | 692.36(453.38-960.97) | 61.88(27.67-123.16) | -20.30(-20.91,-19.68) |
| Guinea-Bissau | 622.90(435.33-859.88) | 64.66(36.16-104.79) | -16.04(-19.55,-12.54) |
| Liberia | 973.26(692.50-1253.91) | 124.18(64.16-238.17) | -30.67(-33.11,-28.24) |
| Mali | 646.67(396.52-924.17) | 89.10(56.99-138.22) | -18.19(-19.03,-17.34) |
| Mauritania | 352.91(214.13-538.73) | 48.38(23.53-95.04) | -9.34(-9.81,-8.88) |
| Niger | 1364.31(1001.57-1746.19) | 200.89(124.01-322.46) | -40.09(-41.01,-39.18) |
| Nigeria | 1023.67(697.91-1272.88) | 263.16(171.10-382.55) | -25.62(-26.35,-24.89) |
| Sao Tome and Principe | 429.52(325.92-540.91) | 16.65(10.44-25.34) | -13.38(-14.38,-12.39) |
| Senegal | 634.77(473.72-806.76) | 43.97(26.35-66.42) | -18.98(-19.95,-18.02) |
| Sierra Leone | 704.73(460.62-1031.26) | 106.43(65.52-175.76) | -19.82(-21.20,-18.43) |
| Togo | 592.18(383.02-883.45) | 121.63(61.95-221.67) | -15.71(-16.17,-15.24) |
| American Samoa | 11.72(7.70-17.28) | 7.98(4.98-12.26) | -0.10(-0.11,-0.10) |
| Bermuda | 4.57(3.58-5.65) | 0.60(0.40-0.79) | -0.13(-0.14,-0.13) |
| Cook Islands | 2.18(1.40-3.33) | 1.50(0.91-2.35) | -0.02(-0.02,-0.01) |
| Greenland | 3.48(1.55-5.32) | 1.32(0.87-1.98) | -0.07(-0.07,-0.06) |
| Guam | 4.37(3.09-6.23) | 4.51(2.94-6.72) | -0.00(-0.01,0.00) |
| Monaco | 0.25(0.12-0.40) | 0.25(0.16-0.39) | 0.00(0.00,0.00) |
| Nauru | 30.58(19.56-45.04) | 13.87(8.45-21.51) | -0.54(-0.58,-0.51) |
| Niue | 17.80(12.16-26.42) | 26.62(14.16-37.79) | 0.37(0.26,0.49) |
| Northern Mariana Islands | 3.92(2.60-5.72) | 2.52(1.67-3.61) | -0.04(-0.05,-0.02) |
| Palau | 37.05(21.63-62.10) | 12.88(8.61-18.98) | -0.77(-0.80,-0.74) |
| Puerto Rico | 4.34(3.80-4.99) | 2.13(1.69-2.72) | -0.07(-0.08,-0.05) |
| Saint Kitts and Nevis | 62.50(54.31-71.53) | 11.63(8.31-16.42) | -1.47(-1.55,-1.39) |
| San Marino | 0.36(0.18-0.53) | 0.11(0.06-0.20) | -0.01(-0.01,-0.01) |
| Tokelau | 49.77(26.42-86.83) | 88.89(52.45-143.75) | 1.82(1.42,2.22) |
| Tuvalu | 79.50(45.07-121.58) | 10.42(6.67-15.30) | -2.18(-2.22,-2.13) |
| United States Virgin Islands | 7.08(4.71-10.49) | 2.38(1.35-3.62) | -0.15(-0.15,-0.15) |
| South Sudan | 938.74(579.41-1335.96) | 449.27(279.14-667.33) | -16.01(-18.03,-13.98) |
| Sudan | 437.57(231.55-792.99) | 28.26(11.12-78.84) | -13.28(-13.62,-12.95) |

Notes: AAPC=Average Annual Percent Change. CI=Confidence interval. GBD= Global burden of disease. UI=Uncertainty interval.

Table S8: The change trend of DALY rate of diarrhea disease among children under 5 years old in 204 countries and territories of GBD from 1990 to 2021.

| Location | DALY rate  (per 100,000populationss)  (95% UI).  1990 year. | DALY rate  (per 100,000 populations)  (95% UI).  2021 year. | AAPC of DALY rate (95% CI).  1990－2021. |
| --- | --- | --- | --- |
| China | 5957.59(4475.71-7487.70) | 108.99(84.53-142.02) | -188.67(-192.74,-184.61) |
| Democratic People's Republic of Korea | 625.55(443.51-895.01) | 245.94(172.38-334.93) | -12.56(-13.29,-11.82) |
| Taiwan (Province of China) | 478.79(417.67-562.33) | 275.49(183.97-411.99) | -6.57(-7.36,-5.78) |
| Cambodia | 27121.00(18931.63-37218.59) | 1567.60(1093.81-2271.06) | -845.86(-864.30,-827.43) |
| Indonesia | 43803.98(28551.05-55394.38) | 3621.45(2729.80-4698.40) | -1265.13(-1294.46,-1235.79) |
| Lao People's Democratic Republic | 67524.18(42510.12-94789.31) | 4314.46(2696.77-6749.26) | -2034.97(-2078.88,-1991.06) |
| Malaysia | 1556.89(1071.56-2007.83) | 422.29(303.90-560.93) | -34.39(-35.74,-33.03) |
| Maldives | 28898.35(18999.67-36631.64) | 1168.66(847.80-1599.51) | -898.79(-915.16,-882.41) |
| Myanmar | 47202.82(22116.97-77205.49) | 3224.90(2159.71-4624.01) | -1458.29(-1472.76,-1443.83) |
| Philippines | 16182.17(11547.69-21002.21) | 2145.43(1636.44-2874.35) | -454.37(-463.91,-444.83) |
| Sri Lanka | 4628.47(3449.06-6118.30) | 372.68(271.30-495.74) | -131.88(-136.63,-127.13) |
| Thailand | 5134.20(2111.06-8754.89) | 766.63(572.55-983.40) | -139.26(-142.28,-136.24) |
| Timor-Leste | 58778.37(32117.66-80817.15) | 4000.98(2337.27-6208.19) | -1783.80(-1804.49,-1763.11) |
| Viet Nam | 3489.87(1844.89-5380.80) | 245.43(169.67-333.12) | -105.48(-107.36,-103.59) |
| Fiji | 3199.26(2233.07-4442.56) | 1710.68(1042.21-2593.45) | -49.13(-52.87,-45.40) |
| Kiribati | 25062.61(18265.53-34531.95) | 5379.01(3374.55-7782.63) | -635.95(-652.11,-619.79) |
| Marshall Islands | 3798.86(2199.63-5871.58) | 1372.07(832.00-2091.60) | -55.78(-114.36,2.79) |
| Micronesia (Federated States of) | 4397.28(2940.63-6330.33) | 873.92(542.09-1320.53) | -66.21(-153.42,21.00) |
| Papua New Guinea | 23017.08(15630.66-34057.30) | 9798.66(5954.52-15370.05) | -435.71(-457.08,-414.34) |
| Samoa | 934.47(439.46-1408.46) | 373.65(208.62-551.72) | -17.68(-18.19,-17.17) |
| Solomon Islands | 7845.02(5071.05-11732.21) | 1636.11(1066.90-2324.46) | -198.88(-209.22,-188.55) |
| Tonga | 1099.63(719.54-1575.61) | 389.63(240.61-600.75) | -21.15(-22.12,-20.19) |
| Vanuatu | 6385.02(3692.88-10530.35) | 1824.19(989.99-3327.82) | -141.46(-154.66,-128.26) |
| Armenia | 9014.39(7643.17-10515.29) | 217.66(167.32-284.63) | -294.76(-314.15,-275.38) |
| Azerbaijan | 15002.47(11534.32-19239.14) | 2147.51(1331.53-3411.87) | -412.79(-419.03,-406.55) |
| Georgia | 3937.03(3305.20-4657.71) | 115.60(87.95-152.75) | -112.22(-123.65,-100.79) |
| Kazakhstan | 8003.74(7029.99-8957.89) | 159.62(118.23-211.93) | -260.70(-275.74,-245.65) |
| Kyrgyzstan | 10805.60(9236.07-12352.70) | 667.63(525.79-836.77) | -338.13(-356.14,-320.11) |
| Mongolia | 5646.51(3447.88-8947.54) | 537.28(215.37-1140.54) | -158.17(-162.88,-153.47) |
| Tajikistan | 32457.53(26346.90-39770.99) | 8785.86(5885.81-12294.45) | -799.75(-824.86,-774.64) |
| Turkmenistan | 24360.53(21071.41-28328.49) | 742.70(551.59-988.70) | -792.05(-820.36,-763.74) |
| Uzbekistan | 10936.49(9646.83-12352.33) | 202.04(152.03-270.63) | -361.93(-381.84,-342.02) |
| Albania | 2273.25(1673.96-3064.34) | 396.58(221.76-581.32) | -61.60(-66.68,-56.52) |
| Bosnia and Herzegovina | 468.39(342.85-610.27) | 237.43(168.90-330.93) | -7.36(-8.14,-6.57) |
| Bulgaria | 419.16(353.16-498.83) | 375.12(283.25-474.79) | -4.27(-5.79,-2.75) |
| Croatia | 204.97(169.09-245.12) | 250.34(183.30-321.73) | 1.08(0.44,1.73) |
| Czechia | 482.87(395.76-580.04) | 501.27(398.80-618.86) | 0.45(-1.81,2.70) |
| Hungary | 497.57(403.61-601.32) | 615.59(460.29-773.40) | 4.79(3.14,6.43) |
| North Macedonia | 6824.59(5339.34-8949.86) | 614.35(445.70-869.70) | -202.39(-211.03,-193.74) |
| Montenegro | 132.60(98.65-180.93) | 43.23(28.61-63.11) | -2.93(-3.08,-2.77) |
| Poland | 223.75(195.04-254.66) | 192.86(150.35-239.66) | 0.48(-0.55,1.51) |
| Romania | 1661.53(1407.72-1933.89) | 387.56(291.83-488.57) | -31.83(-35.75,-27.91) |
| Serbia | 513.91(233.02-768.12) | 153.69(105.63-225.66) | -10.58(-11.62,-9.54) |
| Slovakia | 209.88(152.49-271.55) | 181.96(113.77-300.35) | -0.94(-1.41,-0.46) |
| Slovenia | 109.03(88.14-136.50) | 103.65(79.33-134.86) | 0.03(-0.27,0.32) |
| Belarus | 550.16(456.60-681.07) | 113.44(78.61-152.42) | -13.56(-15.26,-11.87) |
| Estonia | 474.73(403.96-558.45) | 122.12(84.26-167.89) | -11.04(-11.78,-10.30) |
| Latvia | 418.02(352.64-502.40) | 86.96(61.09-115.98) | -10.73(-11.60,-9.86) |
| Lithuania | 563.09(471.11-667.37) | 133.80(100.40-174.38) | -13.91(-15.58,-12.24) |
| Republic of Moldova | 2150.99(1779.71-2558.45) | 271.05(202.95-357.92) | -55.71(-60.22,-51.19) |
| Russian Federation | 1055.27(961.72-1161.98) | 128.47(111.17-151.97) | -30.74(-32.50,-28.98) |
| Ukraine | 596.52(510.66-698.27) | 101.41(81.98-126.08) | -14.83(-16.53,-13.12) |
| Brunei Darussalam | 144.76(103.75-192.92) | 122.88(85.79-171.08) | -0.70(-0.76,-0.64) |
| Japan | 255.47(189.23-347.81) | 255.15(182.51-363.94) | 0.11(-0.23,0.45) |
| Republic of Korea | 311.34(233.48-428.68) | 93.48(64.92-131.58) | -6.87(-7.09,-6.65) |
| Singapore | 208.48(182.72-237.87) | 46.97(37.38-58.96) | -5.14(-5.84,-4.43) |
| Australia | 125.47(101.69-158.22) | 34.19(26.88-44.20) | -2.62(-2.88,-2.35) |
| New Zealand | 190.95(156.74-239.44) | 124.16(101.86-153.45) | -1.33(-1.97,-0.70) |
| Andorra | 184.95(115.38-280.63) | 181.86(107.28-287.29) | -0.51(-0.75,-0.26) |
| Austria | 368.03(244.38-535.41) | 153.90(107.43-223.02) | -7.03(-7.46,-6.59) |
| Belgium | 261.28(202.42-340.71) | 243.90(191.41-311.04) | -1.33(-1.93,-0.73) |
| Cyprus | 588.29(363.30-797.23) | 262.86(182.76-370.36) | -10.80(-11.21,-10.39) |
| Denmark | 283.70(221.64-371.16) | 291.09(215.68-393.96) | 0.55(0.00,1.09) |
| Finland | 190.80(124.88-283.13) | 141.73(95.13-201.98) | -1.41(-1.75,-1.07) |
| France | 292.18(226.50-381.23) | 310.36(218.12-443.87) | 0.33(-0.24,0.90) |
| Germany | 230.40(162.45-332.29) | 157.79(120.97-211.31) | -3.49(-3.88,-3.10) |
| Greece | 184.76(115.34-287.75) | 147.54(95.28-224.63) | -1.36(-1.55,-1.17) |
| Iceland | 193.34(125.09-283.92) | 203.74(133.82-302.85) | 0.14(-0.20,0.47) |
| Ireland | 173.48(110.08-266.31) | 184.01(120.07-277.42) | -0.46(-1.06,0.13) |
| Israel | 287.21(232.55-364.54) | 195.37(153.26-250.97) | -2.80(-3.37,-2.23) |
| Italy | 114.50(79.35-165.48) | 123.01(97.24-153.87) | 0.16(-0.24,0.56) |
| Luxembourg | 224.14(171.69-300.49) | 187.02(142.66-244.24) | -1.36(-1.84,-0.89) |
| Malta | 169.29(104.63-261.42) | 181.74(120.21-275.06) | 0.24(-0.10,0.58) |
| Netherlands | 235.48(157.75-344.88) | 366.75(241.12-544.41) | 4.48(3.81,5.15) |
| Norway | 109.10(70.30-163.66) | 102.09(69.52-149.34) | -0.08(-0.25,0.10) |
| Portugal | 340.33(278.06-423.97) | 130.90(97.20-177.17) | -6.05(-6.68,-5.42) |
| Spain | 208.82(147.60-285.16) | 197.63(142.32-269.03) | -0.33(-0.56,-0.10) |
| Sweden | 99.15(61.97-151.72) | 124.48(89.13-172.09) | 1.00(0.83,1.16) |
| Switzerland | 304.11(221.15-416.73) | 286.39(201.40-392.55) | -0.61(-1.10,-0.13) |
| United Kingdom | 137.89(87.95-208.99) | 89.57(63.34-125.76) | -2.47(-3.28,-1.66) |
| Argentina | 1908.68(1726.40-2091.84) | 232.47(183.75-292.43) | -54.00(-57.92,-50.08) |
| Chile | 1125.03(1015.49-1247.71) | 190.32(154.55-232.33) | -29.68(-30.78,-28.59) |
| Uruguay | 1909.47(1697.50-2127.23) | 337.77(257.80-428.55) | -51.72(-57.99,-45.45) |
| Canada | 120.49(84.02-171.98) | 102.51(81.25-128.02) | -0.45(-0.65,-0.24) |
| United States of America | 161.69(136.22-198.28) | 52.18(45.76-59.35) | -2.88(-3.22,-2.54) |
| Antigua and Barbuda | 1071.04(874.92-1312.73) | 341.29(276.05-408.89) | -21.14(-22.36,-19.91) |
| Bahamas | 1353.69(1072.01-1692.28) | 244.82(180.42-329.38) | -41.22(-43.73,-38.71) |
| Barbados | 899.80(728.13-1069.74) | 222.74(162.70-301.32) | -21.81(-23.55,-20.08) |
| Belize | 8180.73(7096.63-9401.58) | 864.93(672.16-1074.54) | -237.89(-248.75,-227.03) |
| Cuba | 1450.75(1308.17-1597.95) | 240.90(192.92-297.47) | -35.25(-37.90,-32.60) |
| Dominica | 1534.66(1096.89-2068.14) | 1117.92(667.37-1720.93) | -13.91(-15.54,-12.27) |
| Dominican Republic | 19558.16(15421.38-24920.43) | 2259.47(1199.92-3320.17) | -565.60(-577.84,-553.36) |
| Grenada | 1427.89(1142.46-1801.91) | 260.32(201.85-330.31) | -34.51(-35.92,-33.11) |
| Guyana | 14061.69(11344.43-16961.56) | 1727.72(1226.64-2381.48) | -385.04(-424.10,-345.98) |
| Haiti | 78775.87(61860.31-95192.84) | 20099.09(13289.45-28252.23) | -2024.03(-2300.31,-1747.75) |
| Jamaica | 5077.07(4338.72-5950.38) | 510.97(360.25-704.28) | -145.41(-149.71,-141.11) |
| Saint Lucia | 2709.93(2192.73-3319.02) | 444.37(319.81-629.94) | -70.66(-73.20,-68.13) |
| Saint Vincent and the Grenadines | 5252.25(4110.89-6527.34) | 601.34(446.59-811.14) | -150.11(-153.68,-146.54) |
| Suriname | 11941.84(8773.02-15627.38) | 2573.36(1611.21-3709.52) | -307.52(-312.59,-302.44) |
| Trinidad and Tobago | 2066.27(1647.33-2517.41) | 415.62(301.77-580.86) | -52.59(-55.73,-49.46) |
| Bolivia (Plurinational State of) | 20343.48(13551.42-29155.30) | 2165.70(1306.49-3157.42) | -639.27(-665.47,-613.07) |
| Ecuador | 12475.40(11441.59-13602.11) | 355.29(257.55-490.59) | -457.80(-497.06,-418.54) |
| Peru | 12292.72(9754.18-15365.91) | 649.66(392.82-976.01) | -448.00(-483.69,-412.30) |
| Colombia | 7522.21(6457.64-8565.19) | 618.55(428.29-878.69) | -223.10(-229.65,-216.56) |
| Costa Rica | 2087.14(1822.67-2333.80) | 307.74(246.80-380.97) | -57.90(-60.69,-55.10) |
| El Salvador | 20737.87(16861.48-25259.97) | 942.72(597.73-1365.05) | -650.37(-664.89,-635.86) |
| Guatemala | 32723.79(29426.80-36797.66) | 5470.32(4123.32-7206.15) | -879.29(-907.58,-850.99) |
| Honduras | 23473.00(19137.73-28216.10) | 2463.01(1500.40-3521.45) | -690.78(-703.53,-678.03) |
| Mexico | 16369.60(14767.63-18343.28) | 718.49(522.12-968.03) | -516.30(-530.86,-501.74) |
| Nicaragua | 28444.74(23087.37-36652.98) | 1392.36(864.11-2021.87) | -872.18(-889.02,-855.34) |
| Panama | 5429.51(4524.84-6423.25) | 1535.22(1159.79-2014.55) | -131.28(-140.81,-121.76) |
| Venezuela (Bolivarian Republic of) | 11311.72(10562.28-12164.29) | 1997.63(961.72-2773.41) | -244.80(-282.35,-207.26) |
| Brazil | 17172.64(14623.40-19770.02) | 432.13(345.67-532.84) | -545.65(-553.63,-537.67) |
| Paraguay | 8256.06(6307.89-10589.59) | 789.95(443.03-1319.99) | -257.91(-266.85,-248.97) |
| Algeria | 2876.56(1253.56-4613.05) | 401.55(198.41-580.19) | -78.37(-79.80,-76.94) |
| Bahrain | 1317.69(986.39-1749.17) | 222.03(154.87-298.68) | -36.38(-37.24,-35.52) |
| Egypt | 29386.95(21356.92-39273.73) | 2018.65(1397.18-2860.06) | -881.34(-890.07,-872.60) |
| Iran (Islamic Republic of) | 3872.73(2570.08-6462.82) | 133.55(99.76-170.01) | -121.22(-123.62,-118.82) |
| Iraq | 6725.52(4679.42-9476.85) | 1095.99(751.31-1540.25) | -183.09(-191.88,-174.30) |
| Jordan | 1613.34(1142.67-2222.39) | 356.20(257.13-489.99) | -41.38(-42.07,-40.68) |
| Kuwait | 673.37(571.21-796.71) | 148.14(114.42-193.59) | -13.93(-15.75,-12.11) |
| Lebanon | 2497.51(1436.29-3622.42) | 664.33(448.59-1001.08) | -61.81(-65.81,-57.82) |
| Libya | 4798.15(2780.87-7469.65) | 480.02(162.47-814.40) | -133.87(-138.93,-128.82) |
| Morocco | 28159.59(21669.34-36030.51) | 1807.98(1110.13-2822.80) | -830.68(-845.81,-815.55) |
| Palestine | 3579.06(2349.40-5520.44) | 258.39(174.71-381.93) | -108.75(-110.36,-107.15) |
| Oman | 2245.74(1251.90-3810.55) | 396.18(269.29-558.45) | -55.91(-57.75,-54.07) |
| Qatar | 631.06(437.59-881.19) | 171.59(122.80-231.00) | -14.58(-15.19,-13.98) |
| Saudi Arabia | 4694.30(3047.53-7294.99) | 300.67(194.34-429.37) | -140.93(-143.83,-138.03) |
| Syrian Arab Republic | 3336.84(2304.98-4851.93) | 272.65(160.39-386.20) | -97.44(-98.40,-96.49) |
| Tunisia | 2748.06(1313.04-4185.08) | 326.30(162.44-487.26) | -76.85(-78.12,-75.59) |
| T眉rkiye | 7028.96(4878.38-10880.84) | 436.60(322.29-608.24) | -207.61(-212.18,-203.03) |
| United Arab Emirates | 1040.96(669.81-1468.91) | 282.35(211.60-382.12) | -25.04(-26.82,-23.26) |
| Yemen | 46169.64(30574.47-66522.94) | 2592.05(970.31-5176.95) | -1391.21(-1422.01,-1360.41) |
| Afghanistan | 38417.34(24805.76-55427.09) | 6525.98(4241.65-9819.13) | -1125.92(-1180.81,-1071.02) |
| Bangladesh | 23157.78(17257.57-34165.71) | 1438.86(900.97-2344.79) | -711.11(-724.71,-697.51) |
| Bhutan | 54361.06(24222.10-90655.70) | 3515.63(1711.58-6188.75) | -1699.35(-1757.28,-1641.42) |
| India | 36547.21(28042.01-45243.48) | 3150.59(1915.73-4649.55) | -1106.97(-1128.09,-1085.84) |
| Nepal | 38348.31(28470.29-49332.03) | 1832.61(1037.68-2748.17) | -1176.02(-1199.34,-1152.71) |
| Pakistan | 38393.40(30441.81-48138.62) | 4737.77(2874.64-7675.21) | -1087.64(-1100.56,-1074.72) |
| Angola | 96678.18(66665.02-131002.67) | 5485.38(3491.16-7792.45) | -2956.49(-3025.05,-2887.94) |
| Central African Republic | 69168.56(41726.67-94622.09) | 28139.58(16355.14-45600.22) | -1294.74(-1327.99,-1261.48) |
| Congo | 36905.68(23713.19-55883.51) | 4972.54(2278.35-10323.98) | -1032.79(-1070.15,-995.43) |
| Democratic Republic of the Congo | 40736.68(27752.52-54250.98) | 4684.18(2289.13-8760.31) | -1135.01(-1190.77,-1079.25) |
| Equatorial Guinea | 77813.79(44097.06-111411.33) | 1928.61(1012.47-3386.12) | -2533.19(-2627.24,-2439.15) |
| Gabon | 22308.54(11466.51-41067.20) | 2274.79(800.29-5159.14) | -641.81(-656.35,-627.28) |
| Burundi | 36162.89(22656.49-52980.15) | 8052.44(3394.34-16986.14) | -943.41(-975.93,-910.89) |
| Comoros | 25179.62(15123.70-39701.58) | 4614.40(2302.59-7801.26) | -716.81(-801.90,-631.71) |
| Djibouti | 40776.88(27039.88-58653.67) | 4091.23(1987.14-7552.73) | -1108.26(-1225.24,-991.28) |
| Eritrea | 62094.27(43215.64-81513.97) | 8348.63(4895.80-13335.85) | -1842.18(-1918.61,-1765.75) |
| Ethiopia | 51216.18(25990.22-79440.10) | 7710.13(5272.17-11597.72) | -1428.69(-1466.76,-1390.61) |
| Kenya | 35746.84(21795.52-46427.92) | 7389.77(5509.37-9639.67) | -937.53(-963.58,-911.47) |
| Madagascar | 64143.93(50974.27-78183.40) | 18991.82(10802.66-31031.98) | -1463.40(-1491.07,-1435.73) |
| Malawi | 79050.66(49968.61-104059.76) | 7752.97(4576.32-12272.62) | -2386.54(-2446.89,-2326.18) |
| Mauritius | 2768.82(2476.74-3112.69) | 747.09(612.36-873.50) | -61.66(-71.49,-51.84) |
| Mozambique | 55804.41(33438.74-85618.06) | 6750.43(3886.93-11645.04) | -1560.99(-1624.77,-1497.20) |
| Rwanda | 41657.32(25129.04-59275.83) | 6121.43(3844.80-9227.91) | -991.69(-1614.26,-369.11) |
| Seychelles | 1522.62(1153.33-2031.99) | 626.23(411.95-900.43) | -30.78(-38.48,-23.08) |
| Somalia | 71926.80(40906.89-103144.69) | 21213.04(12178.94-31457.99) | -1608.10(-1697.16,-1519.04) |
| United Republic of Tanzania | 40742.16(28767.61-55864.69) | 6283.55(3568.69-10161.67) | -1144.14(-1184.37,-1103.90) |
| Uganda | 40907.48(18128.08-64754.57) | 4819.93(2390.22-8966.46) | -1204.93(-1228.90,-1180.96) |
| Zambia | 58616.76(37463.63-81388.33) | 6953.97(4366.33-10471.58) | -1715.91(-1838.91,-1592.91) |
| Botswana | 24893.40(18819.52-32220.51) | 11299.24(7000.64-16791.70) | -371.58(-494.85,-248.31) |
| Lesotho | 49221.70(40034.51-60803.60) | 26813.51(17295.81-37761.47) | -845.32(-922.23,-768.41) |
| Namibia | 31752.62(23541.61-41200.90) | 10850.61(6324.92-15936.52) | -723.10(-755.40,-690.80) |
| South Africa | 33380.19(28300.38-38660.75) | 8551.85(6559.56-11024.02) | -834.19(-880.44,-787.94) |
| Eswatini | 44263.30(33790.33-55289.47) | 12670.39(7851.63-19562.54) | -1096.28(-1157.04,-1035.52) |
| Zimbabwe | 11860.29(8488.34-14902.97) | 8007.20(4020.10-11705.46) | -224.88(-337.55,-112.20) |
| Benin | 44071.47(28876.07-62008.06) | 7321.82(3385.27-14942.64) | -1217.88(-1245.79,-1189.97) |
| Burkina Faso | 69622.31(46499.26-94354.32) | 16128.23(10690.38-23763.74) | -1667.40(-1717.78,-1617.03) |
| Cameroon | 33215.17(21247.51-50111.63) | 7921.99(3850.26-15366.51) | -816.70(-864.26,-769.13) |
| Cabo Verde | 24495.02(17244.88-33069.41) | 1858.92(1179.39-2732.30) | -643.36(-854.55,-432.17) |
| Chad | 108068.79(70068.17-147981.31) | 49921.52(33399.80-84699.30) | -1986.64(-2090.29,-1883.00) |
| C么te d'Ivoire | 32948.43(23274.38-45205.60) | 6970.68(3941.77-11791.58) | -886.93(-935.91,-837.95) |
| Gambia | 36957.69(24541.59-50307.67) | 5562.85(3722.05-7817.32) | -1021.72(-1073.83,-969.60) |
| Ghana | 26653.78(17003.61-37452.74) | 2921.44(1752.30-4386.00) | -747.90(-776.58,-719.23) |
| Guinea | 61989.53(40771.57-85701.25) | 5574.61(2524.95-11028.84) | -1817.18(-1874.43,-1759.93) |
| Guinea-Bissau | 55689.11(39102.18-76626.78) | 5807.61(3280.60-9359.81) | -1433.90(-1747.01,-1120.79) |
| Liberia | 87178.84(62246.46-112383.66) | 11167.59(5837.23-21261.97) | -2740.69(-2956.50,-2524.88) |
| Mali | 57963.07(35887.77-82421.32) | 7992.54(5149.49-12388.28) | -1629.95(-1707.28,-1552.62) |
| Mauritania | 31769.00(19444.97-48412.72) | 4412.90(2201.17-8553.22) | -840.75(-881.49,-800.02) |
| Niger | 121505.37(89572.37-155370.18) | 17967.20(11167.03-28724.41) | -3552.02(-3635.36,-3468.68) |
| Nigeria | 91430.42(62475.14-113421.76) | 23541.83(15387.24-34089.58) | -2285.44(-2351.18,-2219.70) |
| Sao Tome and Principe | 38664.44(29528.05-48574.90) | 1503.67(946.91-2275.06) | -1215.88(-1296.51,-1135.26) |
| Senegal | 56798.29(42736.30-72025.08) | 4007.05(2456.10-5988.35) | -1696.70(-1782.48,-1610.92) |
| Sierra Leone | 63263.91(41591.47-92320.73) | 9546.31(5906.48-15663.69) | -1779.75(-1902.80,-1656.70) |
| Togo | 53157.45(34618.13-78863.45) | 10928.08(5630.31-19802.73) | -1406.87(-1446.34,-1367.40) |
| American Samoa | 1153.74(791.41-1651.31) | 803.14(533.41-1181.73) | -10.12(-10.86,-9.37) |
| Bermuda | 483.50(389.12-593.62) | 132.55(96.74-181.54) | -11.00(-11.46,-10.54) |
| Cook Islands | 307.84(222.58-415.94) | 261.26(182.55-351.37) | -1.11(-1.72,-0.50) |
| Greenland | 339.41(172.71-501.38) | 127.06(87.10-185.75) | -6.53(-6.75,-6.31) |
| Guam | 503.56(378.23-671.52) | 531.59(384.48-737.82) | 0.49(-0.03,1.01) |
| Monaco | 187.98(123.51-282.96) | 185.78(118.47-285.26) | -0.46(-0.77,-0.14) |
| Nauru | 2856.42(1877.71-4161.79) | 1330.52(860.19-1986.95) | -49.56(-52.47,-46.65) |
| Niue | 1722.49(1218.81-2470.52) | 2468.79(1368.16-3451.09) | 31.85(22.11,41.59) |
| Northern Mariana Islands | 473.10(342.93-633.71) | 348.34(259.47-464.49) | -3.72(-4.74,-2.71) |
| Palau | 3494.05(2147.71-5723.23) | 1238.13(856.98-1780.91) | -71.73(-74.58,-68.88) |
| Puerto Rico | 477.75(414.84-551.23) | 253.69(204.34-313.81) | -7.09(-8.29,-5.88) |
| Saint Kitts and Nevis | 5685.05(4944.12-6473.54) | 1084.20(790.92-1526.24) | -133.33(-140.26,-126.39) |
| San Marino | 182.12(119.46-266.45) | 158.94(96.52-246.66) | -1.01(-1.17,-0.85) |
| Tokelau | 4558.64(2497.29-7859.64) | 7977.78(4709.82-12884.68) | 159.74(123.56,195.91) |
| Tuvalu | 7278.92(4225.27-11032.27) | 987.74(656.27-1426.57) | -198.42(-202.49,-194.34) |
| United States Virgin Islands | 702.58(492.64-1011.64) | 250.63(157.46-361.31) | -14.39(-14.59,-14.19) |
| South Sudan | 84044.74(52189.19-119301.87) | 40111.87(24939.63-59448.03) | -1297.35(-1437.18,-1157.52) |
| Sudan | 39586.74(21142.68-71211.42) | 2683.39(1170.94-7178.68) | -1197.04(-1228.02,-1166.06) |

Notes: AAPC=Average Annual Percent Change. CI=Confidence interval. DALY=disability-adjusted life years. GBD= Global burden of disease. UI=Uncertainty interval.


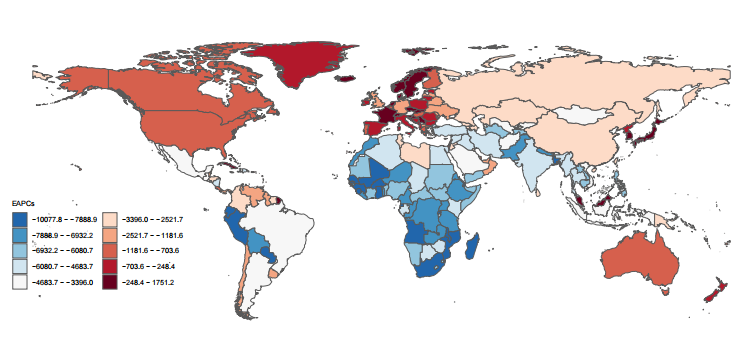


B

A


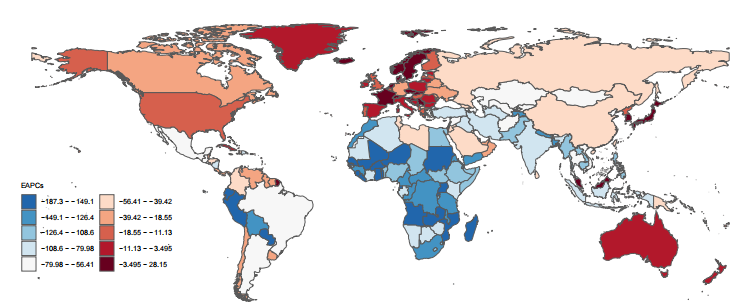


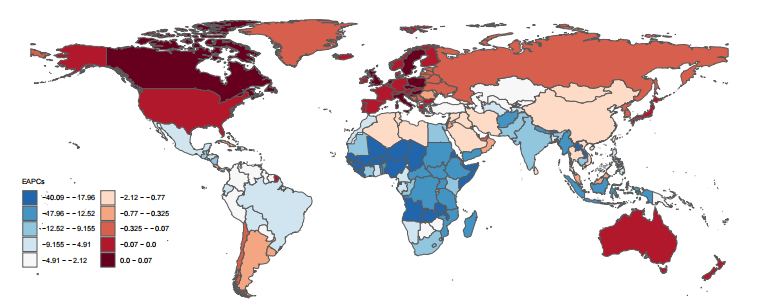


C


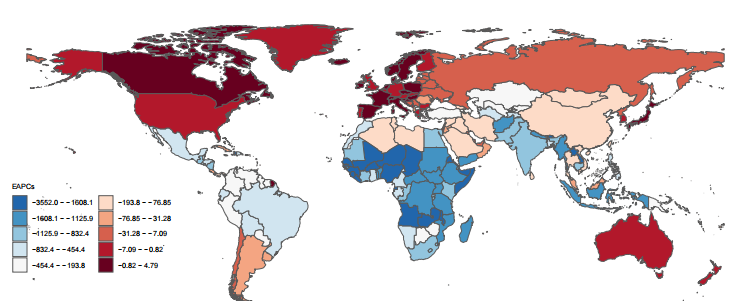


D

Figure S2: AAPC in the incidence rate, prevalence rate, mortality rate, and DALY rate of diarrhea disease among children under five years across 204 countries and territories from 1990 to 2021 (A: Incidence rate, B: Prevalence rate, C: Mortality rate, D: DALY rate. A: Incidence rate , B: Prevalence rate . C: Mortality rate. D: DALYs rate. Abbreviations: AAPC=Average Annual Percent Change. CI=Confidence interval. GBD=Global burden of disease. UI=Uncertainty interval. SDI=Socio-demographic index）


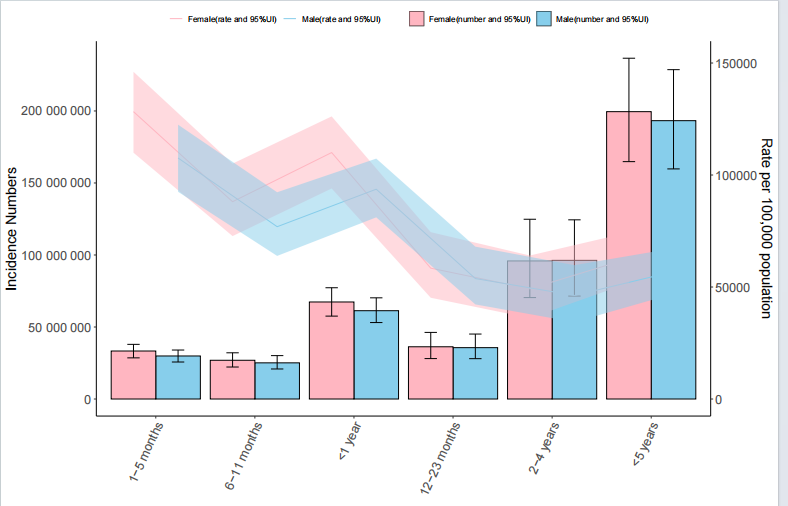


A


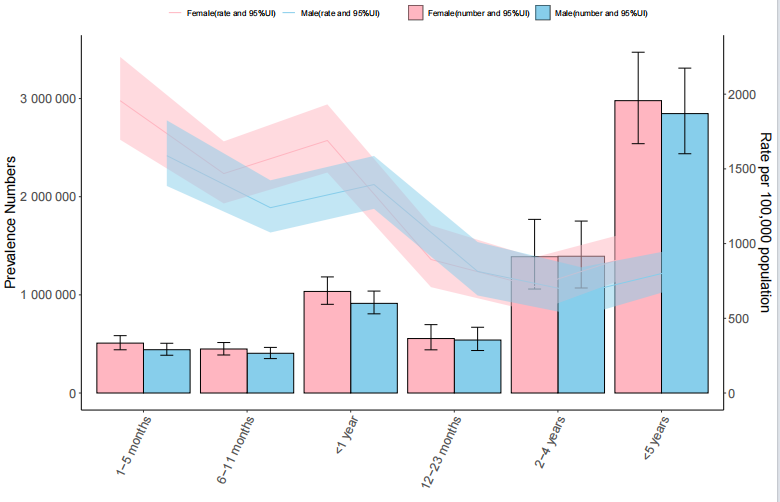


B


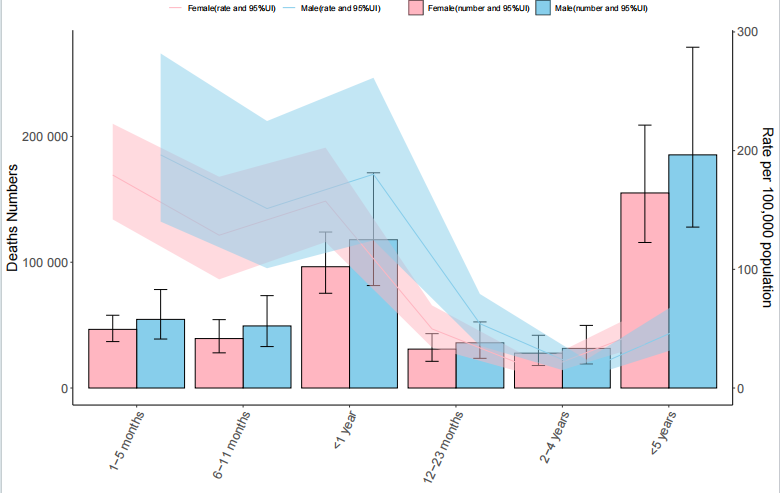


C


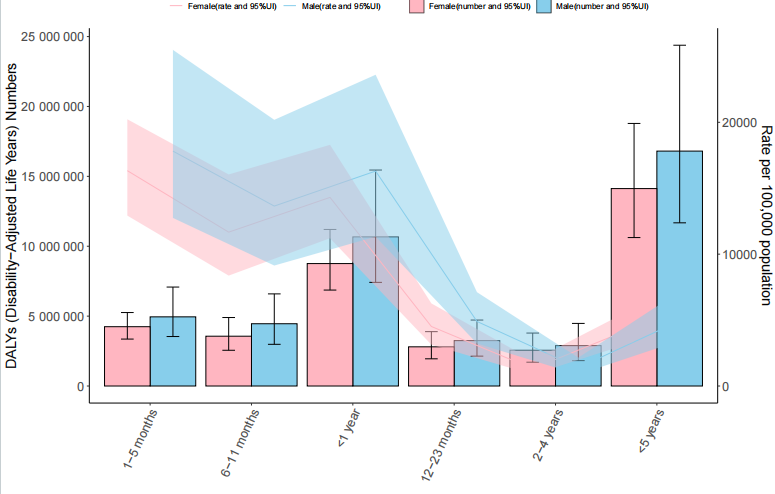


D

Figure S3. The specific rate of diarrheal diseases showed no notable differences across age and gender distributions in children under five years in 2021 year ( A: Incidence, B: Prevalence. C: Mortality. D: DALYs. Abbreviations: DALYs=disability-adjusted life years. UI: Uncertainty interval).


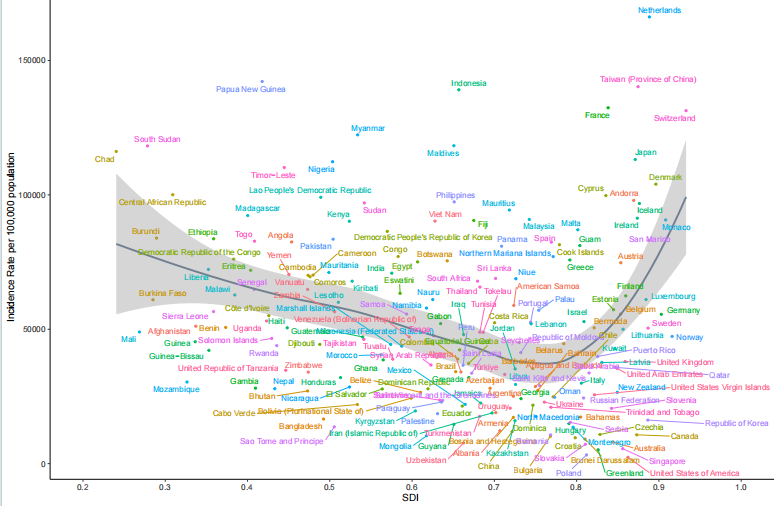


A


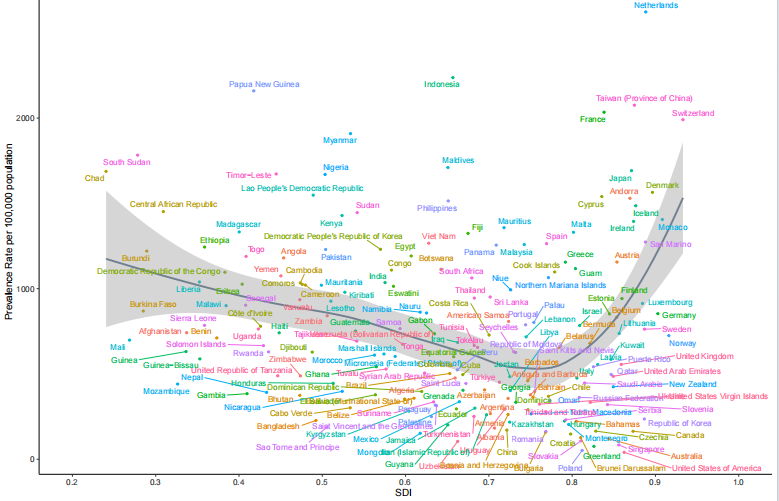


B


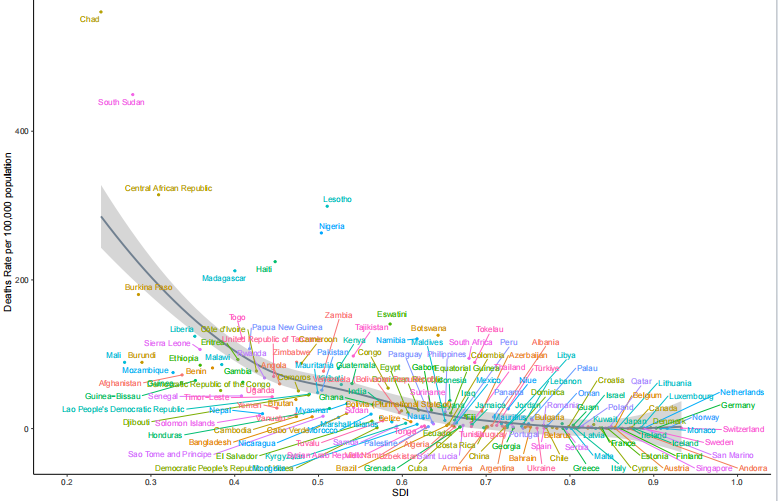


C


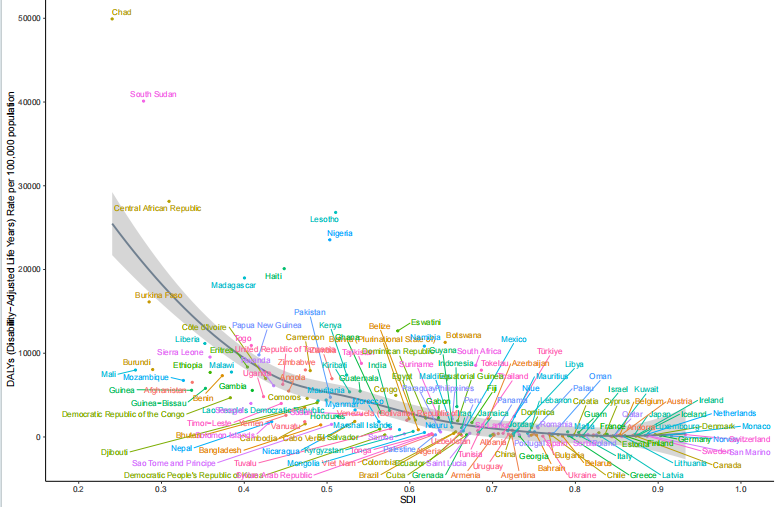


D

Figure S4. The association between the SDI and the rate of diarrhea illness across 204 countries and territories in 2021 year (A: incidence rate. B: prevalence rate. C: mortality rate. D: DALY rate). (Abbreviations: DALYs=disability-adjusted life years. SDI=Sociodemographic Index).


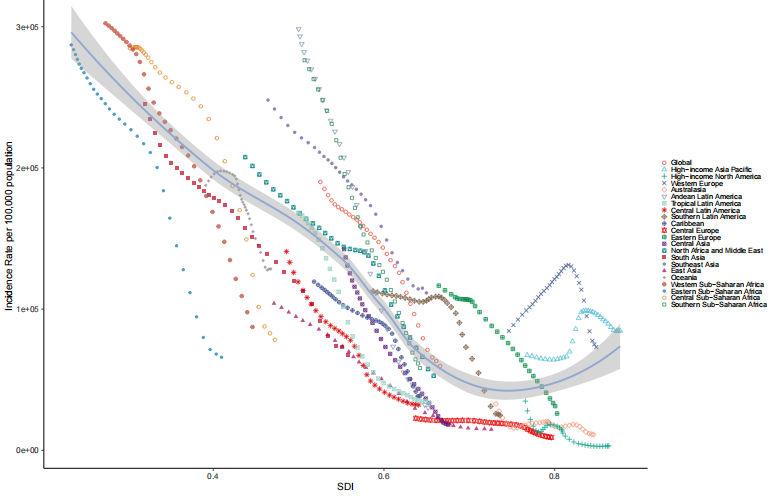


A


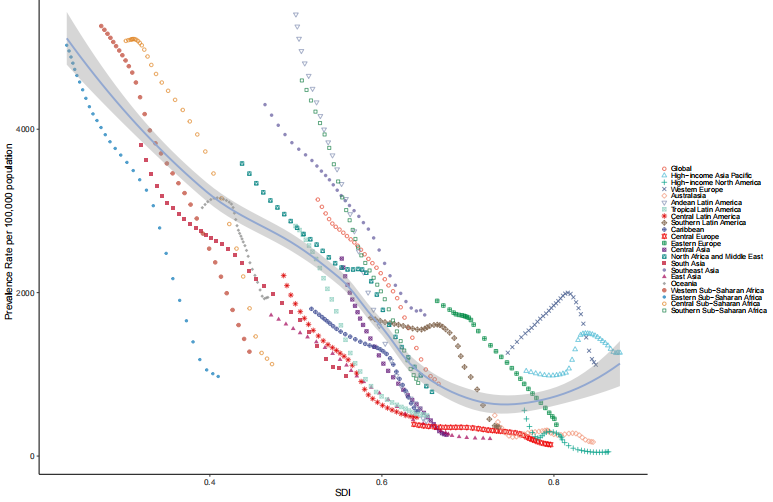


B


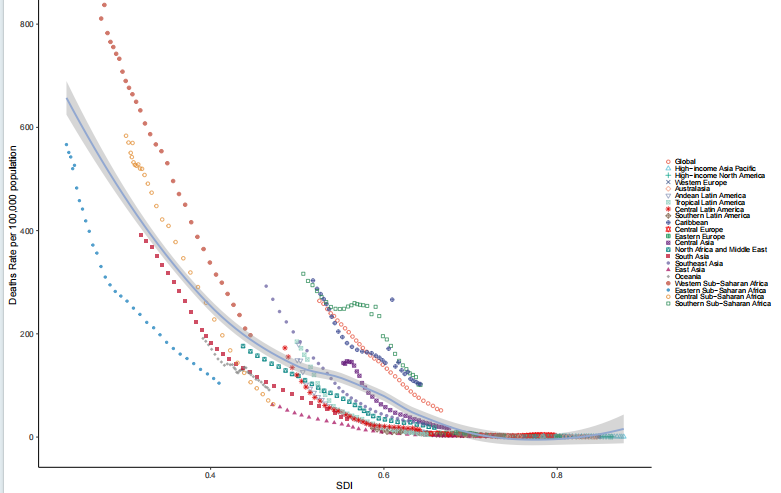


C


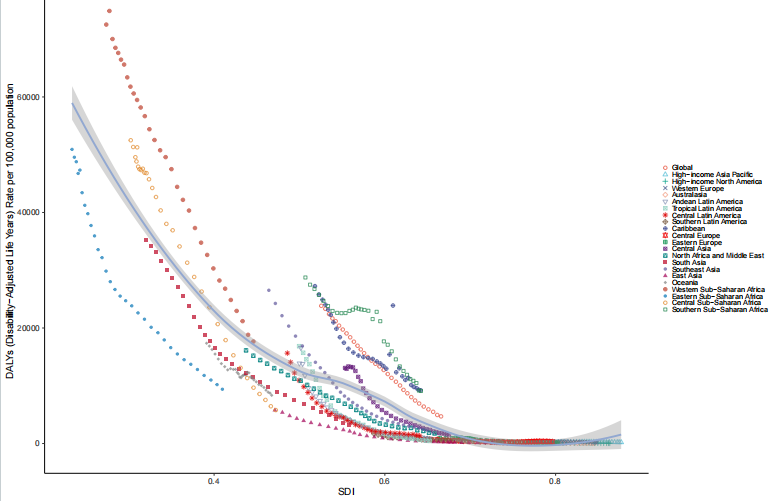


D

Figure S5. The association between the SDI and the rate of diarrhea disease from 1990 to 2021 year (A: incidence rate. B: prevalence rate. C: mortality rate. D: DALY rate). (Abbreviations: DALYs=disability-adjusted life years. SDI=Sociodemographic Index).

Table S9: The change trend of mortality number of children under five years old attributed to specific diarrhea pathogens in global from 1990 to 2021.

| Age-group | Pathogens | Mortality number  (thousands)  (95% UI).  1990 year. | Mortality number  (thousands)  (95% UI).  2021 year. | AAPC of mortality number (95%CI).  1990－2021 year. |
| --- | --- | --- | --- | --- |
| < 5 years | Cholera | 110.11(91.44, 132.85) | 25.78(18.51, 34.92) | -2865.20(-2978.54,-2751.86) |
|  | Non-typhoidal Salmonella | 50.59(7.44, 110.16) | 11.83(1.58, 27.44) | -1259.26(-1275.50,-1243.01) |
|  | Shigella | 299.74(181.25, 484.07) | 81.77(47.92, 137.95) | -7278.54(-7369.29,-7187.78) |
|  | Enteropathogenic E coli | 166.32(95.85, 261.21) | 46.37(25.98, 76.26) | -3960.48(-4055.51,-3865.46) |
|  | Enterotoxigenic E coli | 184.25(107.39, 291.93) | 45.8(25.21, 77.14) | -4501.16(-4602.92,-4399.41) |
|  | Campylobacter | 142.46(63.06, 262.88) | 25.31(10.26, 48.75) | -3844.34(-3882.49,-3806.19) |
|  | Entamoeba | 76.16(36.45, 142.77) | 16.09(7.06, 32.34) | -1937.21(-1962.32,-1912.10) |
|  | Cryptosporidium | 237.55(146.64, 371.65) | 68.48(38.91, 109.57) | -5541.39(-5639.37,-5443.41) |
|  | Rotavirus | 621.83(460.07, 788.8) | 119.86(83.14, 169.41) | -16426.89(-16576.75,-16277.03) |
|  | Aeromonas | 51.49(22.46, 93.91) | 10.74(4.8, 20.18) | -1330.81(-1340.97,-1320.65) |
|  | Clostridium difficile | 0.1(0.05, 0.16) | 0.1(0.05, 0.17) | 0.29(0.18,0.39) |
|  | Norovirus | 138.53(47.38, 248.34) | 29.56(9.65, 55.02) | -3540.77(-3591.12,-3490.42) |
|  | Adenovirus | 352(202.7, 559.7) | 81.08(44.88, 133.04) | -8942.07(-9041.94,-8842.21) |
| < 1 years | Cholera | 55.65(44.05, 68.89) | 12.54(8.44, 17.5) | -1492.54(-1562.55,-1422.53) |
|  | Non-typhoidal Salmonella | 29.52(4.95, 66.03) | 6.68(1.1, 15.48) | -744.16(-753.79,-734.53) |
|  | Shigella | 171.63(96.98, 290.45) | 46.46(25.31, 81.2) | -4139.49(-4189.62,-4089.37) |
|  | Enteropathogenic E coli | 115.09(60.67, 195.15) | 31.89(15.72, 56.37) | -2747.03(-2809.77,-2684.30) |
|  | Enterotoxigenic E coli | 113.88(63.15, 192.68) | 27.18(14.04, 46.77) | -2812.51(-2862.69,-2762.33) |
|  | Campylobacter | 103.16(42.58, 196.17) | 18.2(7.1, 36.25) | -2787.30(-2813.26,-2761.35) |
|  | Entamoeba | 45.17(20.37, 88.85) | 9.59(3.96, 19.4) | -1155.58(-1169.70,-1141.47) |
|  | Cryptosporidium | 144.59(86.57, 231.15) | 42.28(23.94, 69.09) | -3358.28(-3410.68,-3305.88) |
|  | Rotavirus | 394.93(294.45, 499.47) | 77.61(54.14, 108.89) | -10426.42(-10524.67,-10328.17) |
|  | Aeromonas | 21.78(5.41, 52.32) | 4.49(1.13, 11.34) | -567.49(-572.75,-562.23) |
|  | Clostridium difficile | 0.03(0.01, 0.05) | 0.02(0.01, 0.04) | -0.08(-0.12,-0.05) |
|  | Norovirus | 54.61(18.1, 114.57) | 11.66(3.83, 24.29) | -1396.87(-1413.98,-1379.76) |
|  | Adenovirus | 232.95(133.31, 368.07) | 54.6(31.01, 91.19) | -5836.01(-5938.64,-5733.39) |
| 1-2 years | Cholera | 27.48(22.48, 33.73) | 6.33(4.38, 8.92) | -711.42(-741.78,-681.06) |
|  | Non-typhoidal Salmonella | 11.48(0, 32.92) | 2.75(0, 8.35) | -281.98(-286.25,-277.71) |
|  | Shigella | 66.29(34.24, 110.02) | 18.38(9.21, 31.41) | -1587.89(-1608.38,-1567.39) |
|  | Enteropathogenic E coli | 30.26(10.73, 57.69) | 8.54(3.23, 16.2) | -717.92(-732.56,-703.29) |
|  | Enterotoxigenic E coli | 37.55(18.34, 67.4) | 9.82(4.67, 19.27) | -909.24(-921.13,-897.36) |
|  | Campylobacter | 20.32(3.35, 51.87) | 3.8(0.61, 9.23) | -544.39(-552.78,-536.00) |
|  | Entamoeba | 15.88(6.79, 31.32) | 3.36(1.4, 6.94) | -407.52(-411.91,-403.12) |
|  | Cryptosporidium | 49.74(22.83, 91.99) | 13.89(6.35, 25.84) | -1169.15(-1188.22,-1150.09) |
|  | Rotavirus | 121.63(83.64, 167.89) | 23.54(14.84, 35.09) | -3178.71(-3212.99,-3144.42) |
|  | Aeromonas | 15.9(6.06, 31.8) | 3.37(1.24, 6.91) | -407.29(-410.80,-403.78) |
|  | Clostridium difficile | 0.02(0.01, 0.04) | 0.03(0.01, 0.05) | 0.03(-0.00,0.07) |
|  | Norovirus | 44.13(6.34, 82.95) | 9.59(1.4, 18.27) | -1124.64(-1134.31,-1114.96) |
|  | Adenovirus | 63.57(29.96, 120.22) | 14.88(6.92, 26.59) | -1585.50(-1613.36,-1557.64) |
| 2-4 years | Cholera | 26.99(20.54, 34.34) | 6.9(4.49, 9.87) | -658.35(-679.35,-637.36) |
|  | Non-typhoidal Salmonella | 9.6(0, 28.07) | 2.41(0, 7.55) | -233.41(-236.80,-230.03) |
|  | Shigella | 61.82(29.06, 106.25) | 16.93(8.07, 32.09) | -1488.24(-1506.63,-1469.85) |
|  | Enteropathogenic E coli | 20.97(6.78, 41.66) | 5.94(1.98, 12.03) | -490.71(-500.02,-481.39) |
|  | Enterotoxigenic E coli | 32.83(15.95, 56.87) | 8.81(4.05, 16.66) | -790.37(-798.52,-782.21) |
|  | Campylobacter | 18.97(3.13, 48.56) | 3.31(0.53, 8.27) | -508.52(-513.31,-503.74) |
|  | Entamoeba | 15.1(6.86, 29.86) | 3.14(1.32, 6.49) | -384.42(-389.07,-379.77) |
|  | Cryptosporidium | 43.22(21.36, 77.1) | 12.31(5.56, 22.61) | -1007.74(-1022.67,-992.81) |
|  | Rotavirus | 105.27(68.87, 146.9) | 18.71(11.05, 29.68) | -2804.36(-2832.50,-2776.21) |
|  | Aeromonas | 13.8(5.58, 26.26) | 2.88(1.13, 5.57) | -351.55(-356.73,-346.38) |
|  | Clostridium difficile | 0.04(0.02, 0.08) | 0.05(0.03, 0.09) | 0.26(0.20,0.32) |
|  | Norovirus | 39.8(5.86, 76.33) | 8.31(1.14, 16.59) | -1011.63(-1025.64,-997.61) |
|  | Adenovirus | 55.48(29.06, 105.11) | 11.6(5.41, 22.89) | -1406.09(-1426.89,-1385.30) |

Notes: AAPC=Average Annual Percent Change. CI=Confidence interval. DALY=disability-adjusted life years. UI=Uncertainty interval.

Table S10: The change trend of DALY number of children under five years old attributed to specific diarrhea pathogens in global from 1990 to 2021.

| Age-  group | Pathogens | DALY number  (thousands)  (95% UI).  1990 year. | DALY number  (thousands)  (95% UI).  2021 year. | AAPC of DALYs number  (95% *CI*).  1990－2021. |
| --- | --- | --- | --- | --- |
| < 5 years | Cholera | 9769.38(8111.56, 11796.11) | 2283.18(1638.81, 3093.65) | -2865.20(-2978.54,-2751.86) |
|  | Non-typhoidal Salmonella | 4531.75(701.87, 9812.96) | 1061.46(155.45, 2447.92) | -1259.26(-1275.50,-1243.01) |
|  | Shigella | 26879.73(16303.33, 43357.45) | 7344.39(4318.67, 12373.13) | -7278.54(-7369.29,-7187.78) |
|  | Enteropathogenic E coli | 14928.96(8645.58, 23431.44) | 4169.5(2340.82, 6842.4) | -3960.48(-4055.51,-3865.46) |
|  | Enterotoxigenic E coli | 16567.59(9684.53, 26227.23) | 4129.55(2281.5, 6940.5) | -4501.16(-4602.92,-4399.41) |
|  | Campylobacter | 12890.43(5698.83, 23647.18) | 2315.12(978.99, 4411.46) | -3844.34(-3882.49,-3806.19) |
|  | Entamoeba | 6811.3(3300.2, 12738.81) | 1441.16(636.07, 2883.09) | -1937.21(-1962.32,-1912.10) |
|  | Cryptosporidium | 21230.45(13120.04, 33181.11) | 6123.83(3495.92, 9775.96) | -5541.39(-5639.37,-5443.41) |
|  | Rotavirus | 55840.43(41403.64, 70696.02) | 10788.94(7518.47, 15202.23) | -16426.89(-16576.75,-16277.03) |
|  | Aeromonas | 4596.53(2006.35, 8406.55) | 960.83(432.38, 1806.73) | -1330.81(-1340.97,-1320.65) |
|  | Clostridium difficile | 8.42(4.87, 13.89) | 8.78(4.66, 15.21) | 0.29(0.18,0.39) |
|  | Norovirus | 12488.3(4394.59, 22222.42) | 2684.02(921.2, 4941.65) | -3540.77(-3591.12,-3490.42) |
|  | Adenovirus | 31594.54(18205.5, 50373.02) | 7315.65(4046.72, 11970.48) | -8942.07(-9041.94,-8842.21) |
| < 1 years | Cholera | 4987.39(3948.08, 6173.8) | 1123.78(756.59, 1567.9) | -1492.54(-1562.55,-1422.53) |
|  | Non-typhoidal Salmonella | 2661.01(452.37, 5947.32) | 603.46(102.69, 1392.75) | -744.16(-753.79,-734.53) |
|  | Shigella | 15454.28(8753.26, 26153.23) | 4191.71(2297.63, 7310.53) | -4139.49(-4189.62,-4089.37) |
|  | Enteropathogenic E coli | 10370.81(5479.82, 17582.85) | 2881.2(1425.65, 5085.72) | -2747.03(-2809.77,-2684.30) |
|  | Enterotoxigenic E coli | 10266.37(5690.42, 17359.93) | 2460.25(1272.24, 4234.33) | -2812.51(-2862.69,-2762.33) |
|  | Campylobacter | 9338.97(3889.77, 17704.54) | 1663.98(658.01, 3291.53) | -2787.30(-2813.26,-2761.35) |
|  | Entamoeba | 4063.85(1835.5, 7983.22) | 863.64(361.59, 1747.12) | -1155.58(-1169.70,-1141.47) |
|  | Cryptosporidium | 13006.7(7795.21, 20794.7) | 3808.31(2159.05, 6219.35) | -3358.28(-3410.68,-3305.88) |
|  | Rotavirus | 35557.67(26540.35, 44948.1) | 7000.1(4886.13, 9806.35) | -10426.42(-10524.67,-10328.17) |
|  | Aeromonas | 1966.43(495.21, 4708.68) | 407.03(103.59, 1021.44) | -567.49(-572.75,-562.23) |
|  | Clostridium difficile | 2.39(1.25, 4.18) | 2.11(0.98, 4.01) | -0.08(-0.12,-0.05) |
|  | Norovirus | 4981.49(1696.55, 10360.84) | 1074.95(373.94, 2214.72) | -1396.87(-1413.98,-1379.76) |
|  | Adenovirus | 20984.58(12016.63, 33129.86) | 4940.61(2808.82, 8228.12) | -5836.01(-5938.64,-5733.39) |
| 1-2 years | Cholera | 2435.11(1992.84, 2989) | 561.22(388.05, 790.33) | -711.42(-741.78,-681.06) |
|  | Non-typhoidal Salmonella | 1023.72(2.83, 2924.15) | 245.31(0.67, 741.85) | -281.98(-286.25,-277.71) |
|  | Shigella | 5931.12(3058.38, 9857.73) | 1645.32(825.05, 2808.81) | -1587.89(-1608.38,-1567.39) |
|  | Enteropathogenic E coli | 2702.6(968.46, 5153.2) | 763.02(289.27, 1442.21) | -717.92(-732.56,-703.29) |
|  | Enterotoxigenic E coli | 3370.22(1652.37, 6060.95) | 881.77(420.67, 1719.09) | -909.24(-921.13,-897.36) |
|  | Campylobacter | 1832.82(328.51, 4630.88) | 344.41(62.94, 825.52) | -544.39(-552.78,-536.00) |
|  | Entamoeba | 1415.97(611, 2788.23) | 300(126.07, 615.53) | -407.52(-411.91,-403.12) |
|  | Cryptosporidium | 4427.54(2035.44, 8178.77) | 1236(564.93, 2300.86) | -1169.15(-1188.22,-1150.09) |
|  | Rotavirus | 10895.25(7501.97, 15015.84) | 2109.66(1333.66, 3134.65) | -3178.71(-3212.99,-3144.42) |
|  | Aeromonas | 1416(548.07, 2834.23) | 300.07(110.22, 614.05) | -407.29(-410.80,-403.78) |
|  | Clostridium difficile | 2.18(1.26, 3.59) | 2.24(1.11, 4) | 0.03(-0.00,0.07) |
|  | Norovirus | 3954.55(611.64, 7399.41) | 860.81(135.39, 1640.71) | -1124.64(-1134.31,-1114.96) |
|  | Adenovirus | 5685.09(2691.7, 10684.29) | 1334.91(620.27, 2378.61) | -1585.50(-1613.36,-1557.64) |
| 2-4 years | Cholera | 2346.88(1787.49, 2986.29) | 598.19(389.03, 855.5) | -658.35(-679.35,-637.36) |
|  | Non-typhoidal Salmonella | 847.02(5.33, 2451.92) | 212.69(1.61, 660.53) | -233.41(-236.80,-230.03) |
|  | Shigella | 5494.33(2577.03, 9486.28) | 1507.36(716.69, 2859.01) | -1488.24(-1506.63,-1469.85) |
|  | Enteropathogenic E coli | 1855.55(603.22, 3663.92) | 525.28(178.36, 1055.75) | -490.71(-500.02,-481.39) |
|  | Enterotoxigenic E coli | 2931(1435.91, 5040.85) | 787.53(370.74, 1486.42) | -790.37(-798.52,-782.21) |
|  | Campylobacter | 1718.65(325.63, 4304.35) | 306.74(64.37, 736.97) | -508.52(-513.31,-503.74) |
|  | Entamoeba | 1331.48(609.48, 2607.43) | 277.52(119.91, 568.97) | -384.42(-389.07,-379.77) |
|  | Cryptosporidium | 3796.21(1880.1, 6764.28) | 1079.53(491.89, 1975.41) | -1007.74(-1022.67,-992.81) |
|  | Rotavirus | 9387.51(6193.9, 13045.95) | 1679.17(1009.03, 2628.56) | -2804.36(-2832.50,-2776.21) |
|  | Aeromonas | 1214.1(492.68, 2291) | 253.73(103.1, 488.33) | -351.55(-356.73,-346.38) |
|  | Clostridium difficile | 3.84(1.98, 6.89) | 4.43(2.24, 7.81) | 0.26(0.20,0.32) |
|  | Norovirus | 3552.25(600.46, 6736.94) | 748.26(124.52, 1457.91) | -1011.63(-1025.64,-997.61) |
|  | Adenovirus | 4924.88(2587.59, 9308.97) | 1040.12(493.05, 2028.62) | -1406.09(-1426.89,-1385.30) |

Notes: AAPC=Average Annual Percent Change. CI=Confidence interval. DALY=disability-adjusted life years. UI=Uncertainty interval.
